# DIVE: Diversity-Informed Valuation of Ecosystem Functioning

**DOI:** 10.3390/biology15212047

**Published:** 2026-11-09

**Authors:** Asala Mahajna, Ranko Gacesa, Karel J. Keesman, Gert-Jan W. Euverink, Bayu Jayawardhana

**Affiliations:** 1 Wetsus—European Centre of Excellence for Sustainable Water Technology, Oostergoweg 9, 8911 MA Leeuwarden, The Netherlands; 2 Engineering and Technology Institute Groningen, Faculty of Science and Engineering, University of Groningen, Nijenborgh 4, 9747 AG Groningen, The Netherlands; 3 Department of Genetics, University of Groningen and University Medical Center Groningen, Antonius Deusinglaan 1, 9713 AV Groningen, The Netherlands; 4 Department of Gastroenterology and Hepatology, University of Groningen and University Medical Center Groningen, Antonius Deusinglaan 1, 9713 AV Groningen, The Netherlands; 5 Mathematical and Statistical Methods—Biometris, Wageningen University, Radix, Droevendaalsesteeg 1, 6708 PB Wageningen, The Netherlands

**Keywords:** activated sludge, biodiversity-ecosystem functioning, curse of dimensionality, meta-omics, microbiome

## Abstract

**Simple Summary:**

Microorganisms play an important role in wastewater treatment, but the large amount of biological information generated by modern molecular methods can be difficult to interpret and use for managing treatment systems. This study presents a structured approach called Diversity-Informed Valuation of Ecosystem Functioning (DIVE), which simplifies complex biological data into meaningful measures of microbial community diversity and links these measures to treatment performance over time. The approach was tested using several years of data from a full-scale wastewater treatment plant. The results showed that a consistent pattern occurred approximately three weeks before treatment-performance measurements: greater richness, diversity, and evenness were associated with higher subsequent ecosystem functioning, whereas greater dominance showed the opposite pattern. This suggests that changes in the microbial community may provide an early indication of future treatment performance. By providing a consistent and repeatable way to connect microbial diversity with ecosystem functioning, DIVE can help researchers and wastewater treatment professionals better interpret complex biological data and identify microbial-community patterns and time windows that may warrant further investigation.

**Abstract:**

Meta-omics datasets are inherently high-dimensional, complicating their interpretation and limiting their application to ecological and ecosystem-level analyses. We present DIVE (Diversity-Informed Valuation of Ecosystem Functioning), a structured and reproducible workflow that aggregates high-dimensional meta-omics data into ecologically meaningful community-level descriptors for investigating biodiversity–ecosystem functioning (BEF) relationships. Rather than introducing new diversity metrics or statistical estimators, DIVE integrates established alpha-diversity measures with sampling-design-aware temporal association analysis into a unified framework for relating microbial community structure to ecosystem function. The workflow is demonstrated using a multi-year metatranscriptomics dataset from a full-scale activated sludge wastewater treatment plant. DIVE identified a coherent temporal pattern approximately 23 days before ecosystem-functioning measurements, with richness-, diversity-, and evenness-related descriptors showing positive associations and dominance-related descriptors showing associations in the opposite direction. These relationships are interpreted as exploratory biodiversity–function associations rather than evidence of causation or validated prediction. By formalizing the integration of established analytical approaches into a temporally explicit workflow, DIVE supports reproducible and ecologically interpretable BEF analyses of longitudinal meta-omics data and prioritization of candidate relationships for further mechanistic investigation.

## 1. Introduction

Next-generation sequencing (NGS) technologies have emerged as transformative tools in microbiome research, substantially advancing the characterization of microbiome diversity across all domains of life [1,2,3,4,5,6,7]. These technologies facilitate the generation of extensive meta-omics datasets, which encompass a wealth of genetic, transcriptomic, proteomic, and metabolomic information from microbial communities [8]. However, the complexity inherent in these datasets is marked by high dimensionality, stemming from the multitude of species and features that can be detected within a single sample [9]. This phenomenon, often referred to in data science as “the curse of dimensionality,” presents substantial challenges for engineers, scientists, and process operators. The highly dimensional nature of meta-omics data complicates its interpretation, rendering it difficult to extract actionable insights and effectively leverage this information for practical applications [10].

To address the challenges associated with the inherent complexity of meta-omics datasets, especially in contexts where sample sizes may be limited, several methodological approaches can be employed [11]. One strategy involves the application of feature selection and dimensionality reduction techniques derived from machine learning. These methods aim to reduce dataset dimensionality by identifying informative features or transforming high-dimensional data into lower-dimensional representations, thereby facilitating downstream modelling [12]. Common approaches include regularized regression techniques such as LASSO and Elastic Net, as well as dimensionality reduction methods such as principal component analysis (PCA) and autoencoders, which are widely used for handling high-dimensional microbiome data [13]. Importantly, these methods do not necessarily discard data arbitrarily, but rather redistribute, penalize, or project information in ways that can preserve predictive signal while reducing complexity.

However, the choice of method influences how information is represented and interpreted. In some feature selection approaches, particularly those that enforce sparsity, certain variables may be excluded from the final model, which can affect interpretability depending on the ecological question. Moreover, different methods may emphasize prediction performance over ecological interpretability, or vice versa, and therefore introduce different analytical trade-offs rather than universal limitations [14,15,16]. Consequently, while machine learning approaches are powerful tools for managing high-dimensional meta-omics datasets, their application should be aligned with the specific analytical objective. Another alternative approach involves the aggregation of microorganisms into higher taxonomic categories, which serves to directly decrease the dimensionality of the dataset [17,18,19,20]. While this method effectively mitigates the curse of dimensionality, it carries the drawback of potentially compromising data resolution and limiting the capacity for comprehensive cross-microbiome analyses. Community-level diversity descriptors offer an alternative means of reducing complex meta-omics data into ecologically interpretable properties without requiring individual features to serve directly as predictors.

A related challenge arises when microbial-community data are analyzed longitudinally in relation to ecosystem functioning. Existing ecological and microbiome time-series analyses commonly focus on whether two measured signals covary, synchronize, or predict one another over time. For example, ecological studies may examine whether increases in prey abundance precede increases in predator abundance, whether seasonal changes in temperature are followed by changes in phytoplankton biomass, or whether fluctuations in microbial taxa are temporally associated with nutrient concentrations or other environmental variables [21]. Such approaches are useful for characterizing temporal dependence, but they do not necessarily incorporate prior mechanistic knowledge about which biological component generates the ecosystem process of interest.

This distinction is particularly important in systems where the biological driver of ecosystem functioning is known a priori. In activated-sludge wastewater treatment, for example, microbial communities constitute the biological machinery responsible for organic-matter degradation and nutrient transformations. The unresolved question is therefore not whether microorganisms contribute to treatment performance, but which properties of microbial-community structure are most informative about variation in ecosystem functioning. This motivates a biodiversity–ecosystem functioning perspective in which prior mechanistic knowledge defines the direction of inquiry, while complementary diversity descriptors are used to explore which dimensions of community organization are most closely associated with subsequent functional variation.

Therefore, we present DIVE; a reproducible and mechanistically informed workflow integrating complementary alpha-diversity descriptors with sampling-design-aware temporal association analysis to link meta-omics-derived microbial-community structure to ecosystem functioning. Rather than introducing a new diversity estimator or statistical correlation method, DIVE formalizes a biodiversity–ecosystem functioning framework in which established ecological mechanisms constrain the analytical direction, multiple diversity descriptors represent alternative dimensions of community structure, and temporally resolved associations are used to prioritize candidate biodiversity–function relationships for subsequent investigation. Demonstrated using wastewater-treatment data, DIVE provides an ecologically interpretable and reproducible approach for exploratory biodiversity–ecosystem functioning analyses.

## 2. Materials and Methods

### 2.1. DIVE Framework

In our deductive research, we employed biodiversity–ecosystem functioning (BEF) theory as the conceptual framework for constructing an abstraction approach that links microbial-community structure to ecosystem functioning. BEF provides an established ecological basis for examining relationships between biodiversity and ecosystem processes [22]. The novelty of DIVE lies not in proposing a new diversity index or statistical estimator, but in formalizing a mechanistically constrained BEF workflow in which prior knowledge identifies the biological driver and direction of inquiry, complementary diversity descriptors represent alternative dimensions of community structure, and temporally resolved associations are used to prioritize candidate biodiversity–function relationships for subsequent mechanistic investigation [23].

DIVE is designed as a mechanistically informed, exploratory and hypothesis-generating framework rather than as a generic correlation, causal-inference, significance-testing, or forecasting procedure. Its analytical direction is specified before temporal-association analysis on the basis of established ecological or mechanistic knowledge of the studied system.

In the present application, microbial communities constitute the biological machinery responsible for organic-matter degradation and nutrient transformations in activated-sludge treatment. Microbial-community descriptors are therefore treated as candidate representations of the biological community state, whereas BOD, nitrogen, and phosphorus removal represent ecosystem-functioning outcomes. This prior biological knowledge establishes the direction of inquiry but does not imply that an individual diversity descriptor is itself a causal determinant or validated predictor of treatment performance.

DIVE evaluates multiple complementary dimensions of community structure rather than relying on a single diversity index. Richness, diversity, evenness, dominance, and rarity descriptors characterize different ecological properties of the community and may therefore provide different representations of biodiversity–function relationships. Because some diversity descriptors are mathematically related or deterministic transformations of the same underlying property, concordant results among such metrics are not interpreted as independent replication. Interpretation is therefore made primarily at the level of ecological descriptor families rather than by counting individual metrics as separate evidence.

### 2.2. DIVE Input Structure and Analysis Parameters

DIVE is implemented in R using a TreeSummarizedExperiment (tse) object as the primary microbial-community input. The tse object contains the feature-abundance matrix together with the corresponding sample metadata, including the sampling dates required for temporal alignment. Ecosystem functioning is supplied separately through the functionality argument as a numeric vector corresponding to the samples represented in the tse object.

The principal analysis parameters are supplied directly through the dive() function and define how the diversity and temporal-association analyses are performed. The sampling_even argument specifies whether the observations are treated as regularly or irregularly sampled and therefore determines which temporal-association method is applied: lag-specific Pearson correlation for regular sampling or the exact-lag DCF for irregular sampling. The rarefaction_iterations argument controls the number of repeated rarefaction runs used to stabilize the estimation of alpha-diversity descriptors, while rarefaction_depth determines the common sequencing depth to which samples are subsampled before diversity calculation. The max_lag argument defines the maximum temporal separation considered when evaluating associations between microbial-community descriptors and ecosystem functioning and therefore sets the temporal window of the analysis. The seed argument fixes the random-number generator used during stochastic rarefaction, allowing repeated analyses to reproduce the same rarefaction sequence and resulting diversity estimates. The functionality_name argument provides the label of the ecosystem-functioning variable being analyzed and is propagated to the generated tables and figures, whereas output_dir specifies the location in which the corresponding analytical outputs are stored. Together, these parameters make the sampling assumptions, sequencing-depth standardization, temporal search window, and output organization explicit and reproducible for each DIVE analysis.

Before temporal analysis, DIVE preserves the correspondence among the abundance data, sample metadata, and ecosystem-functioning measurements and orders observations chronologically according to the sampling dates stored within the TreeSummarizedExperiment. Sampling dates are therefore obtained directly from the tse sample metadata rather than supplied as an independent date vector.

### 2.3. Rarefraction and Alpha-Diversity Calculation

Sampling effort is standardized through repeated rarefaction before alpha-diversity descriptors are calculated. The user may define the rarefaction depth through the rarefaction_depth argument. When rarefaction_depth = NULL, DIVE automatically sets the rarefaction depth to the smallest total read count observed across all samples in the input dataset. This ensures that every sample can be retained in the rarefaction procedure while standardizing all samples to a common sequencing depth.

For each rarefaction iteration, alpha-diversity descriptors are calculated independently and subsequently averaged to obtain one sample-level estimate for each descriptor. The number of iterations is controlled by rarefaction_iterations, and a fixed seed ensures that the stochastic rarefaction procedure can be reproduced.

Rarefaction standardizes the total abundance used for diversity estimation and thereby facilitates comparison among samples with different sequencing depths. DIVE does not impose an additional global prevalence or abundance filter before diversity calculation. Zero feature counts are retained as absences in the abundance matrix unless their treatment is inherent to the definition or implementation of a specific diversity metric.

Alpha-diversity descriptors are calculated using the mia R package (version 1.20.0) [24]. The metrics used in DIVE and their definitions are provided in Table S1.

### 2.4. Diversity Descriptors and Ecological Interpretation

DIVE characterizes community structure using complementary alpha-diversity descriptors representing richness, diversity, evenness, dominance, and rarity. Richness describes the total number of distinct taxa present, providing a basic measure of community size, although it does not capture abundance distribution structure [25]. Diversity reflects the distribution of abundances across taxa and is commonly associated with ecological complexity and stability [26]. Evenness quantifies how uniformly individuals are distributed among taxa, where higher values indicate more balanced communities [27]. In contrast, dominance captures the extent to which a small number of taxa prevail within the community [28]. Rarity describes the presence of low-abundance taxa that occur infrequently across samples [26].

The term “feature” is used because DIVE can be applied to either taxonomic abundance data or annotated functional-transcript abundance data. When DIVE is applied to functional transcript profiles, the diversity descriptors characterize the overall structure of the annotated functional profile rather than the abundance of only the most dominant transcripts or pathways.

This distinction is important because wastewater-treatment functions emerge from interacting microbial populations and metabolic processes rather than from the action of a single transcript or pathway. Metabolic intermediates generated through one process may serve as substrates for another, and multiple microbial populations may contribute sequentially or simultaneously to aggregate ecosystem functioning. DIVE therefore evaluates community-level functional organization rather than attempting to assign BOD, nitrogen, or phosphorus removal to an individual transcript.

The mechanistic knowledge establishes the direction of inquiry; DIVE evaluates which community-level descriptors show the strongest and most consistent temporal associations with ecosystem functioning.

### 2.5. Assessment of Temporal Sampling Design

Before temporal-association analysis, DIVE inspects and summarizes the intervals between consecutive sampling dates.

The temporal-analysis procedure is selected according to the sampling design using the sampling_even argument:

sampling_even = 1 for regularly sampled observations; and
sampling_even = 0 for irregularly sampled observations.

DIVE does not interpolate irregular observations onto an artificial regular time grid. Instead, regular and irregular datasets are analyzed using different temporal-association estimators.

Regularly sampled observations are evaluated using lag-specific Pearson correlations calculated from overlapping observations available at each lag. Irregularly sampled observations are evaluated using an exact-lag discrete correlation function (DCF) based directly on the observed calendar dates.

The sampling-design specification is therefore an explicit component of the analysis and is retained among the output settings.

### 2.6. Definition and Direction of Temporal Lag

Let $A_{i}$ denote an alpha-diversity descriptor observed at sampling date $t_{i}$, and let $F_{j}$ denote an ecosystem-functioning measurement observed at date $t_{j}$.

Throughout DIVE, temporal lag is defined as:

$$\tau_{i,j}=t_{i}-t_{j}$$

Thus, a negative lag indicates that the microbial-community observation precedes the ecosystem-functioning observation, whereas a lag of zero represents contemporaneous observations.

The current implementation evaluates only negative and zero lags. This directional restriction reflects the a priori ecological question addressed by DIVE: whether a microbial-community state precedes or coincides with subsequent ecosystem functioning. It does not constitute evidence that diversity causally determines later ecosystem functioning and does not exclude feedbacks from environmental or operational conditions to the microbial community.

The maximum temporal window is specified by the user through the max_lag argument.

### 2.7. Temporal Associations for Regularly Sampled Observations

For a shift of k sampling intervals, diversity observations $A_{i}$ are paired with subsequent functioning observations $F_{i+k}$. The correlation is

(1) $$r\left(-k\right)=\frac{\sum_{i\in I_{k}}\left(A_{i}-{\bar{A}}_{k})(F_{i+k}-{\bar{F}}_{k}\right)}{\sqrt{\sum_{i\in I_{k}}({A_{i}-{\bar{A}}_{k})}^{2}\sum_{i\in I_{k}}{\left(F_{i+k}-{\bar{F}}_{k}\right)}^{2}}},$$

where $I_{k}$ contains the complete observation pairs available at shift k, and ${\bar{A}}_{k}$ and ${\bar{F}}_{k}\;$ are the means of the corresponding paired subsets. The means and variances are therefore recalculated using only the observations contributing to each lag.

The resulting values range from −1 to 1, with positive values signifying a positive correlation and negative values indicating a negative correlation [29]. The number of complete observation pairs contributing to each lag-specific estimate is calculated and reported alongside the correlation coefficient.

For regularly sampled observations, lag-specific Pearson correlations were calculated in R using stats::cor (…, method = “pearson”) from the base stats package (version 4.6.1), applied separately to the complete overlapping diversity and ecosystem-functioning observations available at each lag.

### 2.8. Temporal Associations for Irregularly Sampled Observations

For irregularly sampled observations, DIVE uses an exact-lag discrete correlation function, based on the pairwise-product approach of Edelson and Krolik [30]. This approach uses observed dates directly and avoids interpolation onto an artificial regular time grid. Each descriptor series and the functioning series are standardized using their respective overall means and standard deviations:

(2) $$z_{A,i}=\frac{A_{i}\;-\;\bar{A}}{s_{A}},\;z_{F,j}=\frac{F_{j}\;-\;\bar{F}}{s_{F}}.$$

For each observation pair, the unbinned discrete correlation product and its lag are

(3) $$U_{ij}=z_{A,i}z_{F,j},\;\tau_{i,j}=t_{i}-t_{j}.$$

Pairs are retained when $-L\;\leq \;\tau_{i,j}\;\leq \;0$, where L is the maximum lag in days. Non-finite products are excluded. For each observed exact lag τ, define

(4) $$P_{\tau }=\left\{\left(i,j\right):\;\tau_{i,j}=\tau ,U_{ij}\;is\;finite\right\},\;M_{\tau }=\left|P_{\tau }\right|.$$

The exact-lag DCF is then

(5) $$DCF\left(\tau \right)=\frac{1}{M_{\tau }}\sum_{(i,j)\in P_{\tau }}U_{ij}.$$

Unlike a conventional binned DCF, this implementation groups pairs only when their observed day differences are identical. It therefore preserves the exact temporal resolution of the observations, while potentially producing lag estimates supported by few pairs. The number of contributing pairs, $M_{\tau }$, is reported alongside every estimate.

For lags containing at least two pairs, DIVE calculates the sample standard deviation of the pairwise products and its associated standard-error summary:

(6) $$s_{U}\left(\tau \right)=\sqrt{\frac{\sum_{\left(i,j\right)\in P_{\tau }}{\left[U_{ij}\;-\;DCF\left(\tau \right)\right]}^{2}}{M_{\tau }\;-\;1}},\;{\;SE}_{descriptive}\left(\tau \right)=\frac{s_{U}\left(\tau \right)}{\sqrt{M_{\tau }}}.$$

For a single contributing pair, the DCF is retained but its standard deviation and standard error are reported as unavailable. These summaries describe variation among pairwise products.

The exact-lag DCF uses overall-series standardization rather than normalization within each lag-specific subset. Consequently, it is not constrained to the interval −1 to +1 and is not directly equivalent to the lag-specific Pearson correlation. Positive DCF values indicate a predominance of same-sign standardized deviations among the contributing pairs; negative values indicate a predominance of opposite-sign deviations. No measurement-error variance correction is applied.

For irregularly sampled observations, the exact-lag discrete correlation function was implemented directly within DIVE in R following the pairwise-product formulation of Edelson and Krolik, with observation pairs grouped according to their exact calendar-day differences rather than predefined lag bins. Thus, no external R DCF function or package was used for the irregular-sampling analysis. The equations reported in the revised Methods correspond directly to these implemented calculations.

### 2.9. Inferential Scope and Treatment of Temporal Dependence

DIVE is intended as an exploratory temporal-association framework. Accordingly, temporal associations identified by DIVE are not interpreted as evidence of causation, validated prediction, or an independent mechanistic effect of an individual diversity descriptor. Observed associations may reflect microbial-community organization, temporal persistence, shared seasonality, environmental conditions, operational factors, or combinations of these processes.

### 2.10. Interpretation of Multiple Diversity Descriptors

The descriptors evaluated by DIVE represent complementary, but not necessarily statistically independent, dimensions of community organization. Some metrics are mathematically related or deterministic transformations of the same underlying quantity. Consequently, DIVE does not treat the evaluated metric–lag combinations as independent confirmatory hypothesis tests. Concordant associations among mathematically dependent indices are not counted as independent replication.

Interpretation instead considers the magnitude and direction of associations, temporal pattern across related lags, the number of contributing observation pairs, concordance or disagreement among ecological descriptor families, and the biological plausibility of the observed relationship.

A descriptor exhibiting the largest absolute association is therefore regarded only as an exploratory candidate for further investigation and is not interpreted as the “most influential” ecological determinant of ecosystem functioning.

### 2.11. DIVE Outputs

DIVE returns an R object of class dive_result that contains the complete results of the analysis. The returned object includes the calculated alpha-diversity descriptors (alpha), the chronologically aligned ecosystem-functioning measurements (functionality), the corresponding sampling dates (dates), the temporal-association results (correlation), a summary of the sampling structure (sampling), the temporal-association method applied (method), and the analysis settings used for the run (settings).

For reproducibility and downstream inspection, DIVE also writes the principal analytical results to the user-specified output_dir. These include a dated table of the calculated alpha-diversity descriptors (alpha_diversity.csv), the aligned ecosystem-functioning series (functionality.csv), a summary of the temporal sampling structure (sampling_summary.csv), and a table recording the analysis settings (analysis_settings.csv). The latter records the ecosystem-function label, sampling-design specification, maximum lag, number of rarefaction iterations, resolved rarefaction depth, and random seed used in the analysis.

The temporal-association results are written separately according to the sampling design. For irregularly sampled data analyzed using the exact-lag discrete correlation function, results are written to dcf_exact_lag_results.csv. For regularly sampled data analyzed using lag-specific Pearson correlations, results are written to ccf_results.csv. These tables provide the lag-specific association estimates and the corresponding information generated by the selected temporal-association procedure.

DIVE additionally generates graphical summaries of the temporal associations. For each alpha-diversity descriptor, an individual association plot is saved in the correlation_plots subdirectory, with versions both with and without figure titles. For irregular sampling these plots display exact-lag DCF values against lag in days, whereas for regular sampling they display lag-specific Pearson correlation coefficients. Two summary heatmaps representing temporal associations across all alpha-diversity descriptors and lags are also generated, again with and without a title.

The returned dive_result object can additionally be supplied to the plot_dive_timeseries() function to generate time-series visualizations of selected alpha-diversity descriptors together with the ecosystem-functioning variable. This separation between machine-readable output tables, retained R objects, and graphical summaries allows the complete analytical result to be inspected, reused, and reproduced without relying solely on the figures presented in the manuscript.

### 2.12. Computational Reproducibility

The reusable DIVE implementation is separated from the dataset-specific code used for the activated-sludge case study. The general package defines the required input structure, rarefaction procedure, alpha-diversity calculations, sampling-design selection, temporal-association estimators, and output generation, whereas the case-study script supplies the specific TreeSummarizedExperiment, ecosystem-functioning vector, and analysis parameters.

DIVE (v0.1.0) is distributed as an R package and can be installed directly from GitHub using remotes::install_github (“AMahajna/dive”). The repository contains the package source code, documentation, unit tests, and an examples/directory containing example code that demonstrates how to structure the inputs and run the workflow.

For reproducibility, the manuscript analysis records the principal DIVE parameters used for each run, including the sampling-design specification, rarefaction iterations and depth, maximum lag, and random seed. The package-generated outputs additionally retain the aligned data, sampling summaries, analysis settings, temporal-association tables, and graphical summaries required to inspect the analysis.

The availability of an executable example within the package is intended to separate the general DIVE workflow from the Oosterhorn application and to provide users with a template for applying the framework to independent datasets. Package installation instructions, function documentation, example usage, and the analysis source code are provided in the public repository.

### 2.13. Application to the Oosterhorn Activated-Sludge Dataset

The Oosterhorn activated-sludge dataset was used as a case-study application of the general DIVE framework. The analysis follows the same computational structure provided in the executable example distributed with the DIVE package, while supplying the Oosterhorn-specific microbial-community data, ecosystem-functioning variables, sampling design, and temporal parameters.

The microbial-community data were represented as TreeSummarizedExperiment objects containing the feature-abundance matrices and corresponding sample metadata, including the original sampling dates. For the functional analysis, the resulting profiles comprised 4589 annotated functional transcripts across the 32 saline activated-sludge samples. Alpha-diversity descriptors were calculated from the complete annotated functional profile rather than only from the most abundant annotations shown in descriptive tables.

BOD, nitrogen, and phosphorus removal were analyzed separately as ecosystem-functioning variables. Each variable was supplied independently through the functionality argument and evaluated in a separate DIVE run.

Because the Oosterhorn observations were irregularly sampled, the analysis used sampling_even = 0 and therefore applied the exact-lag DCF based on the observed calendar dates. Alpha-diversity values were estimated using 100 rarefaction iterations (rarefaction_iterations = 100), with rarefaction_depth = NULL, a maximum lag of 63 days, and a fixed random seed of 123.

The Oosterhorn analysis therefore represents one dataset-specific implementation of the general workflow demonstrated in the DIVE package example. The example provides users with a reusable template for adapting the same procedure to other TreeSummarizedExperiment objects and ecosystem-functioning variables while modifying only the study-specific inputs and parameter settings.

### 2.14. DIVE Workflow

**Figure 1:**
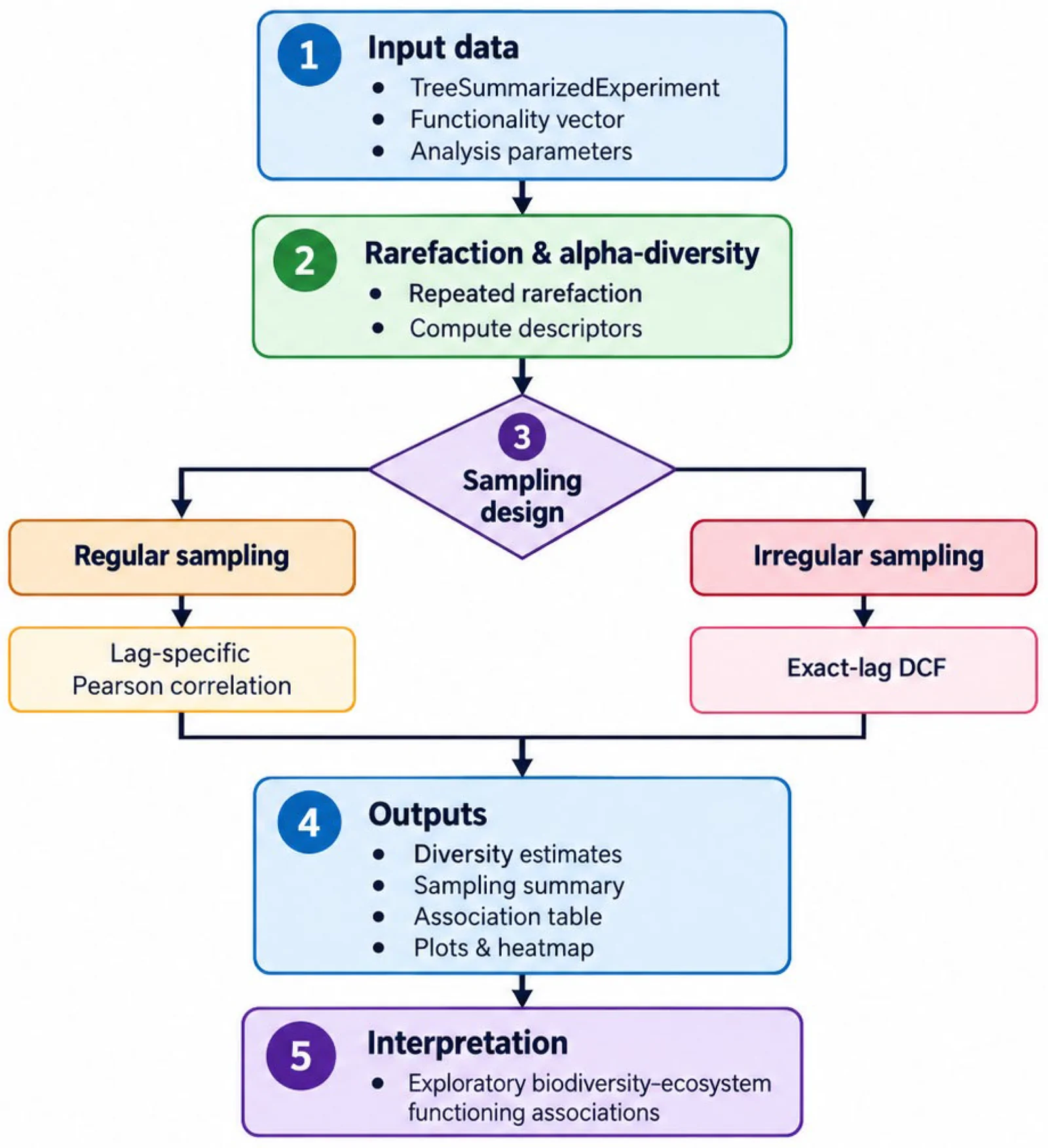
DIVE workflow for exploratory temporal analysis of biodiversity–ecosystem functioning relationships.

Figure 1 summarizes the DIVE analytical workflow. The workflow begins with a TreeSummarizedExperiment containing microbial abundance data and sampling metadata, together with an ecosystem-functioning vector and user-defined analysis parameters. DIVE performs rarefaction and alpha-diversity calculation, evaluates the temporal sampling design, and then selects the appropriate temporal-association method. Regular observations are analyzed using lag-specific Pearson correlations, whereas irregular observations are analyzed using the exact-lag DCF based on actual calendar-day differences. DIVE subsequently reports association estimates, pair support, analysis settings, tabular outputs, and graphical summaries. The resulting relationships are interpreted as exploratory biodiversity–ecosystem functioning associations intended to prioritize candidates for subsequent mechanistic investigation.

## 3. Results

**Figure 2:**
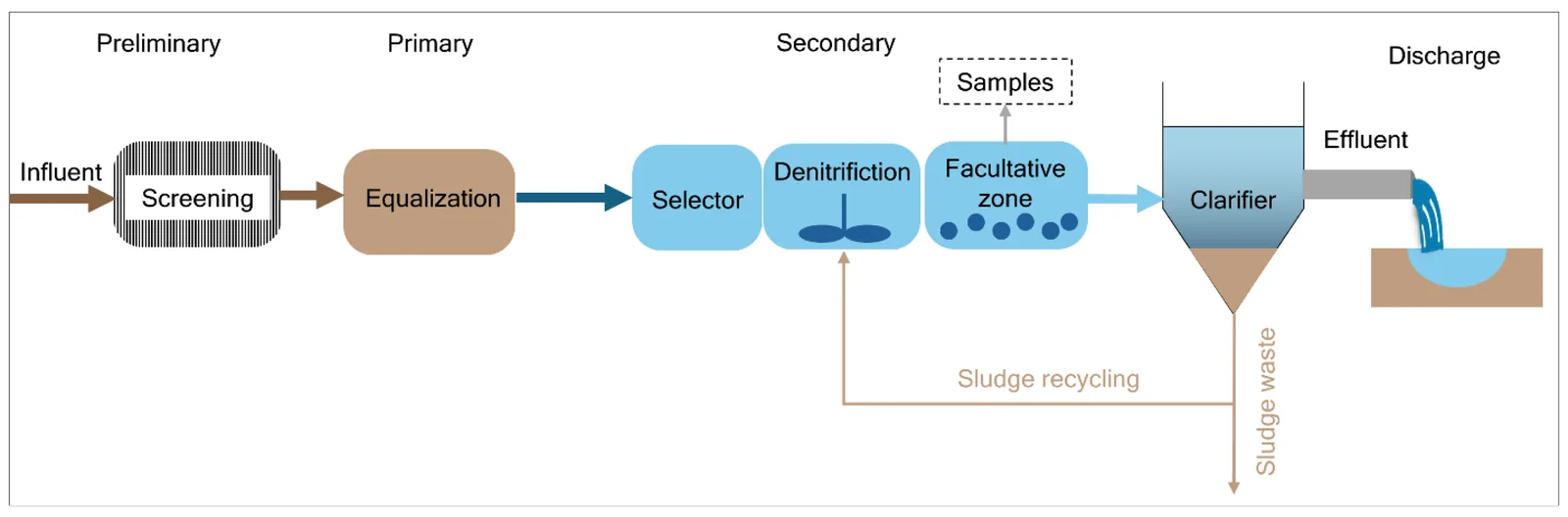
Schematic representation of the treatment process at Oosterhorn SWWTP (adapted from [31]).

To demonstrate the application of the DIVE framework, a publicly available dataset from an activated sludge reactor at an industrial wastewater treatment plant (SWWTP) is utilized [31]. The SWWTP, located at the Oosterhorn industrial park near the harbor district of Delfzijl in the Netherlands, is operated by North Water; a joint venture between Evides Industrial Water and Waterbedrijf Groningen. The plant is managed under a Design, Build, Finance, and Operate (DBFO) agreement. It processes industrial wastewater with a pollution load equivalent to 35,000 PE (PE: Population equivalent is a parameter for characterizing industrial wastewaters), with peaks reaching up to 60,000 PE daily. The plant’s hydraulic capacity is 250 $\frac{m^{3}}{hour}$. Treatment at the SWWTP consists of a preliminary screening phase, followed by primary treatment in an equalization tank, and secondary treatment involving an activated sludge tank and a settling tank. The activated sludge tank is subdivided into three zones: selector, anoxic (denitrification), and facultative zones. The facultative zone’s aeration volume can vary from 0 and 3500 $\frac{{Nm}^{3}}{h}$. The plant also includes a 2500 $m^{3}$ equalization tank and a 6700 $m^{3}$ sludge tank, with a sludge age of 3–4 weeks. Following treatment, the effluent is discharged into the Eems River via the Zeehavenkanaal, eventually reaching the Wadden Sea [32,33]. A schematic process flow diagram is presented in Figure 2.

The published sampling campaign and its respective dataset provide a comprehensive dataset covering a period of two years and four months, with monthly samplings conducted from 2014 to 2017, focusing on the facultative zone of the activated sludge process at a SWWTP. In total, 32 activated sludge samples were sequenced. The collection of meta-transcriptomic data from activated sludge in saline treatment environments is particularly significant due to its ability to shed light on the metabolic activities and gene expression profiles of microbial communities in situ.

Additionally, this genetic dataset is complemented by physical, chemical, and biological measurements taken from various locations within the SWWTP, providing a multi-dimensional view of the treatment process. Similarly, ex situ analyses of wastewater samples from both the influent and effluent streams were performed. In total, 70 other process parameters were monitored. The details of the sampling campaign and lab analysis can be found in the publication of the sampling campaign [31].

### 3.1. Metatranscriptomes Sequencing

The 32 activated sludge samples (8 mL each) were collected in 18 mL scintillation vials, containing a stabilizing solution of 9.5 mL of 96% ethanol and 0.5 mL of 0.1 M sodium citrate (pH 4.2), to ensure RNA integrity during storage at −20 °C until analysis. For sample pre-treatment, 1 mL of each sample was transferred into Lysing Matrix E vials (MP Biomedicals, Solon, OH, USA) and centrifuged at 10,000× *g* for 10 min using an Eppendorf 5424 R centrifuge (Eppendorf AG, Hamburg, Germany) to concentrate microbial cells before RNA extraction.

RNA extraction was performed using a modified protocol from the FastRNA ProSoil Direct Kit (MP Biomedicals, Solon, OH, USA; Cat. No. 116070050). Initially, 700 µL of RNApro Soil Lysis Solution was added to the sample pellet, followed by the addition of 350 µL of acidified phenol/chloroform. The mixture was homogenized for 45 s using a FastPrep-24™ 5G bead-beating grinder (MP Biomedicals, Solon, OH, USA). Afterward, 600 µL of acidified phenol/chloroform was added, and the sample was centrifuged at 10,000× *g* for 3 min at 4 °C to separate the aqueous phase containing RNA, which was then transferred to a new tube. RNA was purified using a spin column, and to ensure the removal of contaminating DNA, the extracted RNA underwent DNase treatment using RQ1 RNase-Free DNase (Promega, Madison, WI, USA; Cat. No. M6101). Further purification was conducted using the Zymo RNA Clean & Concentrator-5 Kit (Zymo Research, Irvine, CA, USA; Cat. No. R1015), with the final RNA eluted in 30 µL of nuclease-free water. Concentration was measured using Qubit™ RNA High Sensitivity (HS), Broad Range (BR), and Extended Range (XR) Assay Kit (Thermo Fisher Scientific, Waltham, MA, USA; Cat. No. Q32852).

The purified RNA was then used for cDNA synthesis with the SensiFAST cDNA Synthesis Kit (Bioline, London, UK; Cat. No. BIO-65053), employing random hexamers for priming. Following first-strand synthesis, the second cDNA strand was synthesized using DNA Polymerase I Large (Klenow) Fragment (Promega Corporation, Madison, WI, USA; Cat. No. M2201). Concentration and quality were measured using a Qubit Fluorometer (Thermo Fisher Scientific, Waltham, MA, USA) with the Quant-iT™ DNA High Sensitivity Assay Kit (Thermo Fisher Scientific; Cat. No. Q32851). Finally, libraries were prepared using the Nextera XT DNA Sample Preparation Kit (Illumina, San Diego, CA, USA; Cat. No. FC-131-1024), and sequencing was performed on the MiSeq platform. Across the 32 paired-end samples, the median GC content was 48.25% (range, 41–53%), the median mean read length was 138.80 bp (range, 88.79–146.82 bp), and the median proportion of duplicated reads was 70.25% (range, 52.34–83.20%). Sample-specific sequencing quality metrics are provided in Table S2. In addition, the detailed outputs from FastQC and MultiQC are also made available online [34].

### 3.2. Bioinformatics Pipeline

Quality assessment of the sequence data was conducted using FastQC [35] v0.12.126 and MultiQC [36] v1.22.2, with default parameters and comprehensive technical validation of the sampling effort. The quality and representativeness of the underlying metatranscriptomic dataset were comprehensively evaluated in the previously published scientific data descriptor [31]. This technical validation included quality assurance of extracted nucleic acids and sequencing libraries, in silico assessment of sequencing-read quality, evaluation of sequencing effort, and ecological validation using rarefaction and feature-accumulation analyses. These assessments demonstrated adequate sequencing effort and sampling coverage for characterizing the taxonomic and functional features represented in the dataset. Full details of the sampling campaign, laboratory procedures, sequencing quality control, bioinformatics processing, and technical validation are provided in [31].

Community profiling was performed on the total RNA-Seq dataset using the Galaxy platform [37]. Raw reads were pre-processed with Trimmomatic v0.39 [38] to remove adapter sequences and trim low-quality ends using a sliding-window approach with a quality threshold of 30 averaged over four bases, while reads failing overall quality requirements were discarded. Taxonomic classification was performed using Kraken2 v2.1.3 [39] with a minimum base quality of 30, a minimum of two hit groups, and a confidence score threshold of 0.1, against the RefSeq PlusPF database [40] downloaded on 15 July 2024. Species-level abundance estimates were then re-estimated using Bracken v3.0 [41]. Total RNA-Seq data were used for community profiling to provide a broad representation of the microbial community, including taxa with high ribosomal RNA (rRNA) abundance that might otherwise be underrepresented.

Figure S1 contains Krona visualizations for all analyzed samples, providing an interactive representation of the taxonomic composition of each microbial community. The hierarchical structure of the Krona plots allows users to explore the data across multiple taxonomic levels, from broad classifications to progressively more specific groups, by zooming into successive taxonomic branches. These visualizations provide a complementary overview of community composition and facilitate inspection of dominant and less abundant taxa within individual samples. Because the plots are provided for every sample, they also allow qualitative comparison of taxonomic patterns across the full sampling series and improve transparency of the taxonomic-classification results underlying the community profiling analysis. In Figure S1, the upper 32 Krona charts display abundance estimates after species-level refinement using Bracken v3.0, whereas the lower 32 charts display the corresponding Kraken2 classifications before Bracken refinement. The repeated sample identifiers refer to the same samples represented at these two processing stages, rather than duplicated biological samples.

**Figure 3:**
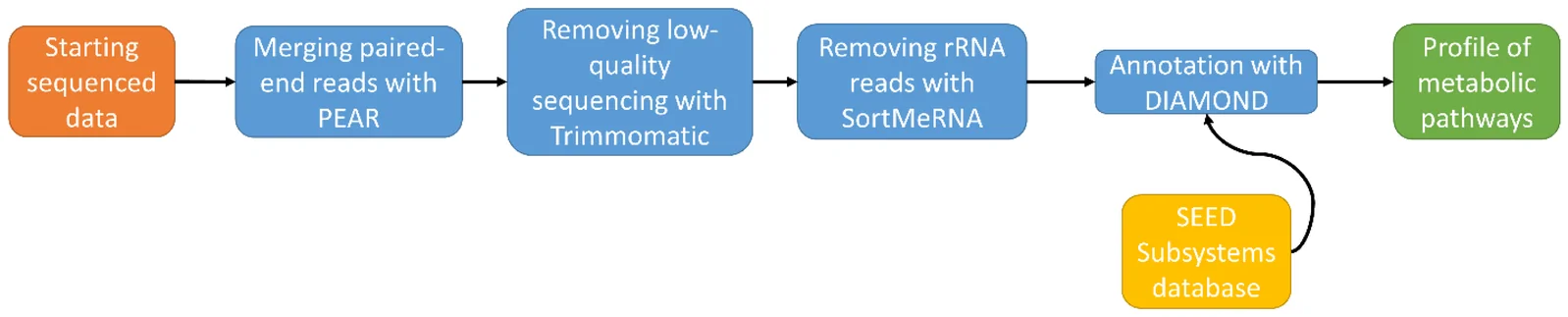
Workflow of the bioinformatics pipeline applied in this study.

For functional profiling, raw sequence reads underwent pre-processing and analysis using SAMSA2 [42] (Simple Analysis of Metatranscriptomes through Sequence Annotation, version 2). The workflow of the bioinformatics pipeline implemented in SAMSA2 is illustrated in Figure 3. Initially, SAMSA2 merges paired-end reads from high-throughput sequencing data using PEAR (Paired-End reAd mergeR) [43]. The pipeline subsequently removes adapter sequences and trims low-quality ends utilizing Trimmomatic [38]. To focus on functional analysis, rRNA sequences were excluded from the total RNA-seq data using SortMeRNA [44]. Then, SAMSA2 characterizes the functional transcript profile by assigning non-rRNA sequencing reads to functional annotations through alignment against the SEED reference database [45] using DIAMOND [46]. The resulting read-derived abundance matrix represents the distribution of annotated functional transcripts across samples and forms the input for the diversity metrics evaluated in DIVE.

The outputs from SAMSA2 provide quantitative information on the abundance and distribution of annotated functional transcripts across the 32 saline-activated sludge transcriptomes. Functional annotations are assigned according to the SEED Subsystems framework, a curated database that organizes functional roles into a hierarchical structure of subsystems and functional categories. The resulting read-derived abundance matrix therefore represents the relative distribution of annotated functional transcripts across samples. The detailed output of SAMSA2 is made available online [47].

Functional diversity descriptors were calculated from the complete annotated transcript profile. This community-level representation was used because wastewater-treatment performance emerges from interacting metabolic pathways and functional guilds rather than from the activity of a single transcript or pathway.

The resulting functional profiles comprised 4589 annotated functional transcripts across the 32 saline-activated sludge samples. These annotated functional transcripts were primarily associated with a core bacterial community belonging to the phyla Bacillota, Bacteroidota, Campylobacterota, Pseudomonadota, and Thermodesulfobacteriota. The top 10 most abundant annotated functional transcripts are found in Table 1. The hierarchal grouping of these genes is based on SEED subsystems.

To interpret the roles of the top 10 most abundant annotated functional transcripts in activated sludge, drawing upon established scientific knowledge is possible. Functional categories such as RNA metabolism, carbohydrate metabolism, respiration, protein metabolism, and membrane transport are essential in supporting microbial activity and promoting effective contaminant removal. For instance, RNA metabolism, involving genes like Retron-type reverse transcriptase and DNA-directed RNA polymerase beta subunit, supports transcription and RNA processing, which are vital for microbial growth and adaptation to environmental conditions [48]. In carbohydrate metabolism, enzymes such as Particulate methane monooxygenase C-subunit and Methanol methyltransferase drive carbon cycling through methane oxidation and methanogenesis, both central to biodegradation [49,50]. Respiratory processes, crucial for energy generation, are supported by Methanol dehydrogenase in electron donation and Cytochrome c oxidase in terminal electron acceptance, with ATP synthase alpha chain ensuring ATP production for microbial survival [51,52]. Protein metabolism, sustained by Translation elongation factor 1 alpha subunit, maintains efficient protein synthesis, while TonB-dependent receptors facilitate nutrient uptake through membrane transport [53,54]. In addition, there was a significant amount of unclassified sequences grouped under “no hierarchy” annotated functional transcripts which accounts for what is known as metatranscriptomic dark matter [55].

The interpretation of the functional roles of the thousands of annotated functional transcripts within the complex activated sludge process using 32 samples poses significant challenges. The sheer volume of this dataset features-wise, coupled with limited samples, is neither practically informative for operational applications nor statistically robust. Therefore, DIVE offers an actionable and statistically reliable framework for understanding microbial community dynamics is this case. The resulting read-derived abundance matrix therefore represents the relative distribution of annotated functional transcripts across samples and serves as the input for the diversity, evenness, dominance, and related metrics evaluated in DIVE. DIVE was applied to the read-derived abundance matrix of SEED functional annotations generated using the SAMSA2 workflow.

### 3.3. Rarefaction and Alpha-Diversity Calculation

To standardize sampling effort before diversity estimation, the functional count matrix was subjected to repeated rarefaction to a common depth of 3366 functional counts per sample, corresponding to the smallest library size in the input matrix. Diversity descriptors were calculated for each of 100 rarefaction iterations and averaged across iterations, using random seed 123. No additional global prevalence or abundance filter was imposed before diversity calculation; metric-specific default settings were retained.

The associated data descriptor evaluated sequencing coverage using taxonomic and functional rarefaction curves and feature-accumulation analyses [31]. These assessments supported adequate recovery of the represented features. In the present analysis, rarefaction additionally served to standardize the functional count total used for diversity estimation.

### 3.4. Definition and Estimation of Ecosystem Functioning

After generating the abundance data of the community in the ecosystem, ecosystem functioning is defined in terms of removal efficiency within the SWWTP, which is attributed to the activities of the activated sludge microbiome. DIVE uses the provided abundance data and ecosystem functioning measure (which is defined as the removal efficiency in the SWWTP) to assess the relationship between both. DIVE calculates all alpha diversity metrics and computes DCF for each and every alpha diversity metric in respect to the removal efficiency. The ecosystem functioning measure is user-defined in DIVE and is case-specific. In our case study, as metatranscriptomes provide insights into the overall functional activity of microbial communities, ecosystem multifunctionality is quantified by calculating removal efficiency (RE) across multiple water quality parameters. Specifically, RE was calculated using the weekly averaged measures of biological Oxygen demand (BOD) as follows:

(7) $$RE\left(t\right)=\frac{C_{in}\left(t\right)-C_{out}\left(t\right)}{C_{in}\left(t\right)}$$

where $C_{in,}\left(t\right)$ is the weekly averaged value of the BOD in the influent at time t and $C_{in}\left(t\right)$ is the weekly averaged value of the BOD in the effluent at time t.

Since this sampling campaign was conducted primarily for microbiome analysis rather than specifically for diversity-informed valuation of ecosystem functioning, some discrepancies in the data were noted. In particular, temporal discrepancies were observed between the water quality measurements and the microbiome data. To address this temporal misalignment and establish proxy synchrony, subsequent water quality measures that closely correspond to the microbiome measurements were utilized. Table 2 displays the timing of the microbiome measurements alongside their respective water quality metrics.

**Figure 4:**
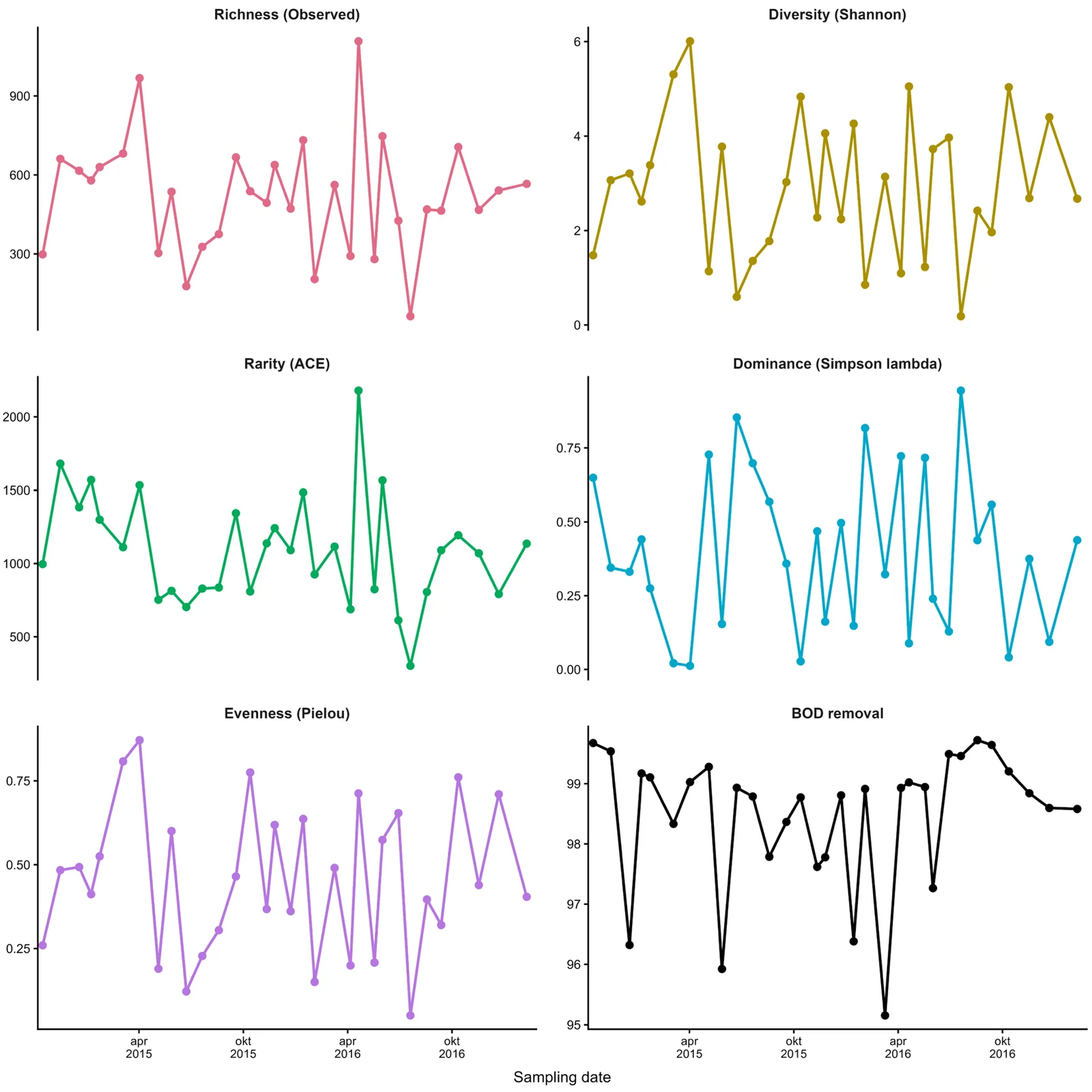
Temporal dynamics of representative microbiome alpha-diversity descriptors and ecosystem functioning. The six time-series panels show representative descriptors of microbiome structure, including richness, diversity, rarity, dominance, and evenness, together with the corresponding removal-efficiency measure over the sampling period. The selected descriptors represent distinct ecological dimensions of community organization and are shown to facilitate visual comparison between temporal changes in microbiome structure and treatment performance.

Figure 4 comprises six time-series plots illustrating the temporal dynamics of representative alpha-diversity descriptors of microbiome structure, including richness, diversity, rarity, dominance, and evenness, together with the corresponding ecosystem-functioning measure (removal efficiency). These representative descriptors were selected to illustrate distinct ecological dimensions of community organization and to visualize how changes in microbial-community structure co-occur with or precede variation in treatment performance over the sampling period.

### 3.5. DIVE: Temporal Associations Between Microbial-Community Structure and Ecosystem Functioning

**Figure 5:**
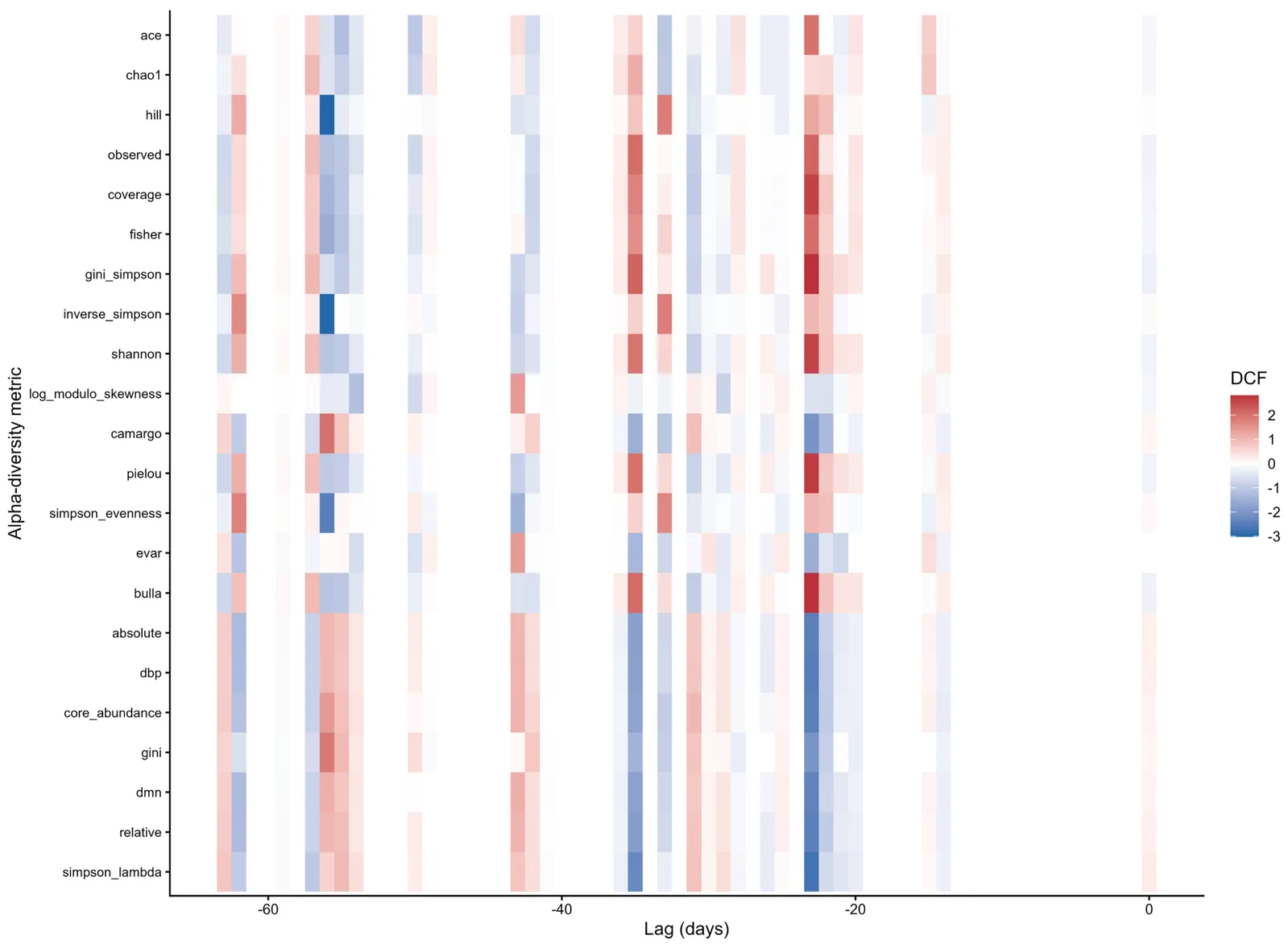
Temporal associations between microbial-community alpha-diversity descriptors and ecosystem functioning. The heatmap shows exact-lag discrete correlation function (DCF) estimates for each alpha-diversity descriptor across the observed negative and zero calendar-day lags. Negative lags indicate that the microbial-community descriptor preceded the ecosystem-functioning measurement, whereas lag 0 represents contemporaneous observations. Positive and negative DCF values indicate corresponding directions of temporal association. Because the number of contributing observation pairs varies among exact lags, DCF magnitude should be interpreted together with pair support rather than as an independent measure of statistical evidence. The heatmap is intended to visualize temporal patterns and concordance among ecologically related descriptor groups.

The exact-lag DCF analysis identified substantial variation in both the direction and magnitude of temporal associations among the different alpha-diversity descriptors and evaluated time lags. Figure 5 summarizes these relationships as a heatmap, providing an overview of the temporal association structure across all descriptors.

The heatmap revealed that temporal relationships were not uniform across diversity descriptors. Instead, groups of ecologically related metrics frequently showed similar directional patterns at particular lags, while several individual metric–lag combinations exhibited comparatively large DCF values.

The most coherent cross-metric pattern occurred at approximately −23 days, where multiple descriptors representing different dimensions of community organization showed large associations in a consistent direction. Richness-, diversity-, and evenness-related descriptors were predominantly positive at this lag, including observed richness (DCF = 2.17), coverage (DCF = 2.61), Fisher richness (DCF = 2.06), Gini–Simpson diversity (DCF = 2.81), Shannon diversity (DCF = 2.59), and Pielou evenness (DCF = 2.70). In contrast, several dominance-related descriptors showed large negative associations at the same temporal separation, including absolute dominance (DCF = −2.46), DBP (DCF = −2.53), core abundance (DCF = −2.46), and Gini dominance (DCF = −2.04). This opposing direction across descriptor families indicates that the −23-day pattern was characterized by greater richness, diversity, and evenness together with lower dominance.

The opposing behavior of these descriptor families is ecologically coherent because richness, diversity, and evenness describe complementary aspects of community composition and abundance distribution, whereas dominance-related descriptors characterize the concentration of abundance among fewer community components. Thus, the −23-day pattern suggests that a community state characterized by greater richness, diversity, and evenness, together with lower dominance, was associated with ecosystem functioning measured approximately three weeks later.

Importantly, this pattern should be interpreted at the level of broader ecological dimensions rather than as independent confirmation from each individual index. Several of the descriptors are mathematically related, and concordance among them therefore reflects a consistent underlying community-structure signal rather than multiple statistically independent observations.

The approximately three-week temporal separation is also ecologically relevant in the context of activated-sludge systems. Rather than implying that a single transcriptional or community snapshot persists directly for 23 days, the observed lag may reflect a broader microbial-community state or ecological reorganization that develops together with system conditions and is subsequently associated with treatment functioning. Changes in richness, evenness, diversity, and dominance may therefore reflect shifts in the representation of microbial populations or functional capacities that collectively participate in organic-matter degradation and nutrient transformations.

**Figure 6:**
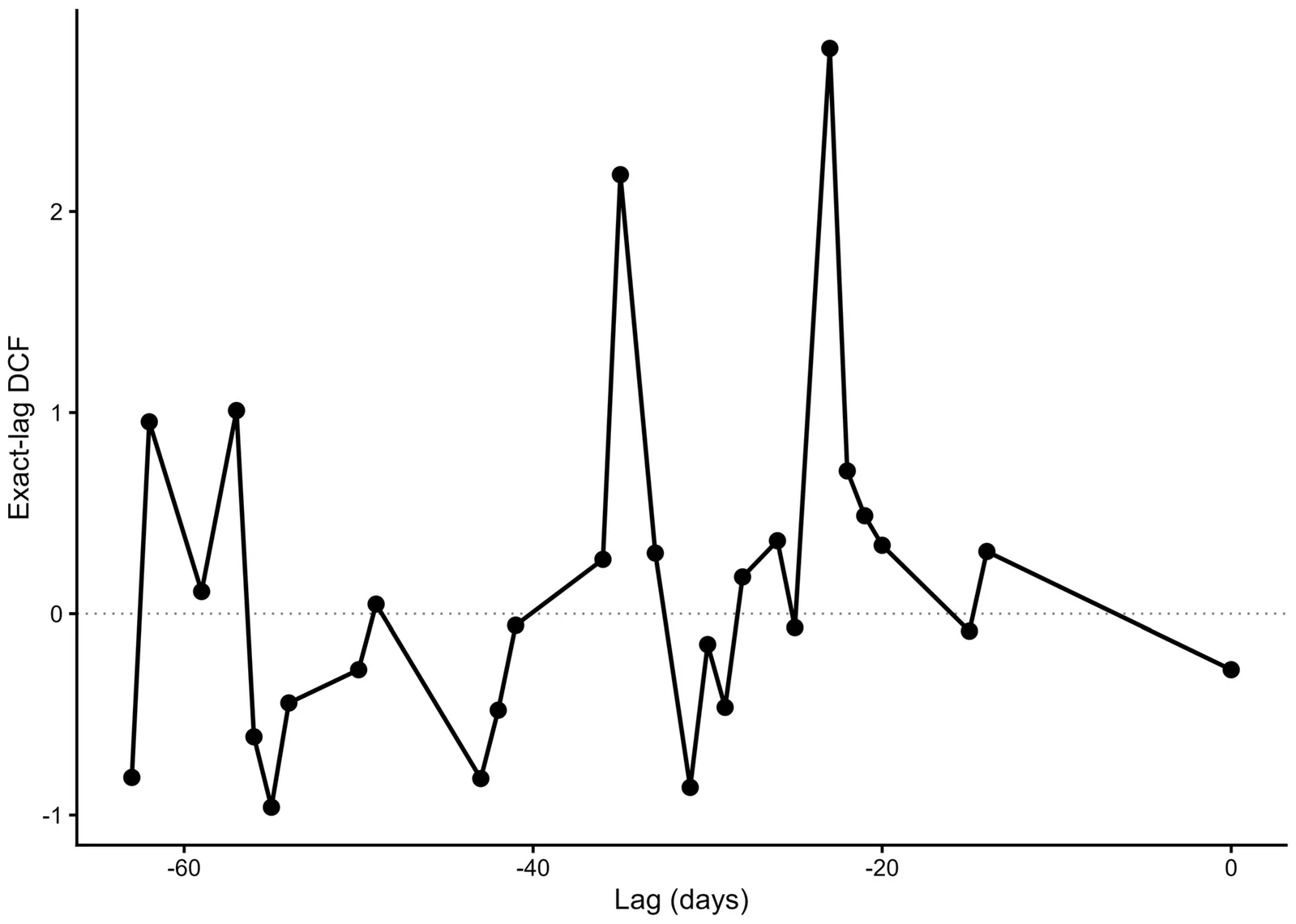
Exact-lag temporal association between Gini–Simpson diversity and ecosystem functioning. The plot shows the discrete correlation function (DCF) between Gini–Simpson diversity and ecosystem functioning across the observed negative and zero and calendar-day lags. Negative lags indicate that the microbial-community diversity observation preceded the ecosystem-functioning measurement. The largest absolute DCF for Gini–Simpson diversity occurred at −23 days (DCF = 2.81), coinciding with the broader cross-metric pattern of positive richness-, diversity-, and evenness-related associations and negative dominance-related associations at this temporal separation. The −23-day estimate was supported by one exact observation pair and is therefore interpreted together with the complete lag profile and concordance across descriptor families rather than as an isolated measure of association strength. Pair support varies among exact lags and should be considered when interpreting DCF magnitude.

However, the −23-day exact lag was represented by only one observation pair in the present irregularly sampled dataset as illustrated in Figure 6. The magnitude of these DCF values therefore cannot by itself establish the robustness of the association, despite the pronounced concordance across descriptor families. The value of this observation lies primarily in the consistency of the ecological pattern across complementary representations of community structure, identifying the approximately three-week temporal window as a candidate for further investigation.

Accordingly, the −23-day pattern is interpreted as an exploratory biodiversity–ecosystem functioning signal rather than as evidence of causation or validated prediction. Future analyses with denser temporal sampling and greater pair support will be required to determine whether this cross-metric pattern recurs consistently and whether it remains associated with ecosystem functioning after accounting for operational and environmental covariates.

The relationship between biodiversity and ecosystem functioning has been extensively investigated, with a substantial body of ecological research demonstrating that variation in community diversity can be associated with changes in ecosystem processes [56,57]. More recent work has further emphasized that biodiversity effects cannot necessarily be reduced to species richness alone, because the distribution of abundance and the functional characteristics of community members may also influence ecosystem functioning [58,59,60]. Evenness, in particular, has been proposed as an important component of biodiversity–ecosystem functioning relationships because a more balanced representation of functionally differentiated community members may influence the breadth and distribution of ecological processes performed by multispecies assemblages [61]. The temporal patterns observed here are consistent with this broader ecological perspective. The concordance of positive richness-, diversity-, and evenness-related associations and negative dominance-related associations at approximately −23 days suggests that the organization of the microbial community, not simply the number of community components present, may be relevant to subsequent variation in ecosystem functioning. 

This distinction is particularly relevant in activated sludge, where ecosystem processes emerge collectively from a complex microbial assemblage rather than from a single taxon or metabolic pathway. The observed relationships therefore support considering multiple dimensions of community organization when investigating biodiversity–ecosystem functioning relationships in engineered microbial ecosystems. Nevertheless, the present temporal associations do not establish direct mechanistic effects of richness, evenness, diversity, or dominance.

More broadly, the value of the DIVE framework lies in enabling high-dimensional meta-omics information to be represented through ecologically interpretable community-level descriptors and related temporally to ecosystem functioning. In this way, the framework provides a means of extending biodiversity–ecosystem functioning concepts commonly developed in macroecological systems to microecological systems. Rather than establishing universal biodiversity effects, DIVE provides a mechanistically informed and reproducible approach for identifying candidate community-level biodiversity–function relationships that can subsequently be examined through targeted mechanistic or confirmatory studies.

## 4. Discussion

This study presents DIVE as a structured and reproducible framework for translating high-dimensional meta-omics data into ecologically interpretable community descriptors that can be related to ecosystem functioning over time. The principal contribution of DIVE is methodological and conceptual integration rather than the introduction of a new diversity index or statistical estimator. DIVE combines established biodiversity descriptors with sampling-design-aware temporal association analysis within a biodiversity–ecosystem functioning (BEF) framework. In doing so, it provides a transparent means of reducing complex molecular profiles to community-level ecological properties and evaluating whether those properties are associated with contemporaneous or subsequent variation in ecosystem functioning.

A central feature of DIVE is that temporal analysis is embedded within a biologically constrained ecological question rather than applied as a generic search for correlations between time series. Prior mechanistic knowledge identifies the biological component responsible for the ecosystem process and therefore establishes the direction of inquiry, while complementary diversity descriptors represent alternative dimensions of community structure. Temporal analysis is then used to identify which of these dimensions show the strongest and most coherent associations with ecosystem functioning. This distinction is important because temporal precedence alone does not establish causation or prediction. Instead, DIVE uses prior ecological knowledge to constrain the analytical direction and treats the resulting temporal relationships as candidate biodiversity–function associations for subsequent mechanistic investigation.

The activated-sludge case study illustrates this logic. Microbial communities constitute the biological machinery responsible for organic-matter degradation and nutrient transformations in wastewater treatment. The relevant question is therefore not whether microorganisms contribute to treatment functioning, but which properties of microbial-community organization are associated with later variation in ecosystem functioning. The exact-lag DCF analysis revealed that these relationships were not uniform across diversity descriptors or temporal separations. Instead, ecologically related groups of descriptors frequently exhibited concordant patterns at particular lags.

The clearest cross-descriptor pattern occurred at approximately −23 days. At this temporal separation, richness-, diversity-, and evenness-related descriptors showed positive associations with subsequent ecosystem functioning, including observed richness, coverage, Fisher richness, Gini–Simpson diversity, Shannon diversity, and Pielou evenness. Conversely, several descriptors reflecting dominance or concentration of abundance, including absolute dominance, DBP, core abundance, and Gini dominance, showed associations in the opposite direction. This opposing behavior across descriptor families is ecologically coherent because richness, diversity, and evenness characterize broader or more balanced representations of community abundance, whereas dominance-related descriptors quantify the concentration of abundance among fewer community components.

The approximately three-week pattern therefore suggests that ecosystem functioning may be related not only to how many community features are represented, but also to how their abundance is distributed. A community state characterized by greater richness, diversity, and evenness together with lower dominance was associated with ecosystem functioning observed approximately 23 days later. This should not be interpreted to mean that increasing biodiversity necessarily improves treatment performance. Rather, the result indicates that the organization of the microbial community may contain information about subsequent variation in functioning and that different dimensions of biodiversity may contribute complementary ecological information.

This interpretation is consistent with broader BEF theory. Biodiversity–function relationships cannot necessarily be reduced to species richness alone, because the relative abundance, functional characteristics, and distribution of community members can influence how ecological processes are performed [50,51,52,53,54,55]. Evenness may be particularly relevant because a more balanced representation of community members can influence the distribution of ecological functions across an assemblage. In microbial ecosystems, this perspective is potentially important because ecosystem processes emerge from interacting metabolic pathways, functional guilds, and intermediate transformations rather than from a single organism or transcript.

At the same time, concordance among multiple diversity indices should not be interpreted as independent statistical replication. Several of the descriptors included in DIVE are mathematically related or emphasize overlapping properties of community structure. Agreement among Shannon diversity, Gini–Simpson diversity, Pielou evenness, and related measures therefore indicates a coherent ecological dimension of community organization rather than multiple independent confirmations of the same relationship. DIVE is consequently most informative when patterns are interpreted at the level of broader descriptor families, such as richness, diversity, evenness, rarity, or dominance, rather than by counting individual metrics that show similar behavior.

The −23-day result also requires particular caution because the exact lag was represented by a single observation pair in the present irregularly sampled dataset. The ecological interest of the −23-day pattern therefore derives primarily from the concordant direction observed across complementary descriptor families rather than from the magnitude of any single DCF estimate. More replicates and denser temporal sampling will be required to determine whether this approximately three-week pattern recurs with greater observational support.

The present findings nevertheless illustrate why community-level abstraction can be useful for high-dimensional meta-omics data. Rather than requiring individual taxa, genes, or transcripts to serve directly as predictors, DIVE summarizes the underlying profile using descriptors with established ecological interpretations. This allows high-dimensional molecular data to be examined in terms of ecological properties such as richness, evenness, dominance, and rarity. Such abstraction does not replace feature-level analysis; instead, it provides a complementary level of organization that can help identify which broad dimensions of community structure warrant subsequent mechanistic investigation.

This framework is not limited to activated-sludge systems but is applicable wherever prior knowledge links a biological community to a measurable ecosystem process. In anaerobic digesters, DIVE could relate microbial diversity and community organization to later variation in methane production, volatile fatty acid accumulation, or organic-matter conversion; in soils, longitudinal microbial profiles could be linked to decomposition, nutrient mineralization, nitrification, denitrification, carbon cycling, or greenhouse-gas production, particularly under seasonal change, drought, fertilization, or other disturbances. In plant–microbiome systems, rhizosphere and root-associated community structure could be related to subsequent nutrient uptake, biomass, yield, or stress responses, while in aquatic ecosystems microbial and phytoplankton diversity could be examined in relation to primary production, oxygen dynamics, carbon fixation, and nutrient cycling. DIVE may also be applied to host-associated microbiomes, where longitudinal community profiles could be related to clearly defined functional outputs such as substrate degradation or microbial metabolite production. Across these systems, prior biological knowledge defines the direction of inquiry, while DIVE evaluates which dimensions of community organization show temporally structured associations with the corresponding ecosystem function.

Across these applications, the value of DIVE lies in separating the general analytical framework from system-specific biological interpretation. The same workflow can be used to calculate complementary diversity descriptors, characterize the temporal sampling structure, select an appropriate association method, and evaluate candidate lags. However, the ecological meaning of the resulting relationships remains specific to the system, measured community, ecosystem function, and temporal scale. An association between diversity and functioning in wastewater treatment should therefore not be assumed to generalize automatically to soils, plants, aquatic communities, or host-associated microbiomes.

DIVE should consequently be viewed as a hypothesis-generating framework rather than a universally validated predictive model. Its purpose is to narrow a large space of possible community descriptors and temporal relationships into a smaller set of ecologically interpretable candidates. Temporal precedence in DIVE is therefore interpreted as an exploratory lead–response relationship rather than as demonstrated predictive utility or causation.

A further consideration is the influence of sequencing effort on diversity estimates derived from abundance data. Although rarefaction is incorporated into the current DIVE workflow to standardize sequencing depth before diversity calculation, different diversity descriptors retain different sensitivities to abundance structure and detection of low-abundance features. Richness-oriented measures may respond strongly to the detection of rare features, whereas proportional diversity and evenness descriptors emphasize the relative distribution of abundance. Examining concordance across these ecological dimensions therefore provides useful context for interpretation, although it does not eliminate all effects of sequencing depth, feature detection, or preprocessing choices.

The irregular sampling structure of longitudinal ecological datasets represents another important methodological consideration. Rather than treating sampling dates as equally spaced, the current DIVE implementation uses an exact-lag discrete correlation function for irregularly sampled observations. Associations are calculated from the actual calendar-day differences between microbial-community and ecosystem-functioning measurements, without interpolation or lag binning. This preserves the original temporal information in the data but results in unequal numbers of contributing observation pairs across lags. Reporting pair support alongside every DCF estimate is therefore essential, particularly for sparsely represented temporal separations such as the −23-day pattern observed in the present case study.

Future work should evaluate DIVE across independent ecosystems, treatment configurations, sequencing approaches, temporal resolutions, and sampling designs. Application to denser longitudinal datasets will be particularly valuable for assessing whether candidate temporal windows recur consistently. Comparisons across systems may further reveal whether particular biodiversity–function relationships are context dependent or whether broader patterns emerge across microbial ecosystems.

Overall, DIVE provides a reproducible framework for moving from high-dimensional meta-omics profiles toward ecologically interpretable biodiversity–ecosystem functioning analyses. The activated-sludge case study demonstrates how complementary descriptors of richness, diversity, evenness, and dominance can reveal distinct temporal patterns in relation to ecosystem functioning, while simultaneously illustrating the need to account for lag-specific pair support and the limitations of observational associations. The broader contribution of DIVE is therefore not the identification of a universally optimal diversity descriptor, but the formalization of a mechanistically informed workflow for generating and prioritizing testable biodiversity–function hypotheses from complex longitudinal meta-omics data.

## 5. Conclusions

DIVE provides a reproducible and ecologically interpretable framework for linking longitudinal meta-omics-derived community structure with ecosystem functioning. By integrating complementary diversity descriptors with sampling-design-aware temporal analysis, DIVE identifies candidate biodiversity–function relationships without implying causation or validated prediction. The activated-sludge case study demonstrates its utility for prioritizing ecologically meaningful temporal patterns and highlights its potential for broader application across microbial ecosystems.

## Figures and Tables

**Table 1:** The top 10 most abundant annotated functional transcripts found across the 32 activated sludge samples.

SEED Subsystem Functional Category	Description	SEED Subsystem	Annotated Functional Transcript
RNA Metabolism	RNA Metabolism	Group_II intron-associated genes	Retron-type reverse transcriptase
			NO HIERARCHY
Carbohydrates	Central carbohydrate metabolism	Particulate methane monooxygenase (pMMO)	Particulate methane monooxygenase C-subunit (EC 1.14.13.25)
Respiration	Electron donating reactions	Respiratory dehydrogenases_1	Methanol dehydrogenase large subunit protein (EC 1.1.99.8)
RNA Metabolism	Transcription	RNA polymerase bacterial	DNA-directed RNA polymerase beta-subunit (EC 2.7.7.6)
Protein Metabolism	Protein biosynthesis	Translation elongation factors eukaryotic and archaeal	Translation elongation factor-1 alpha-subunit
Carbohydrates	One-carbon Metabolism	Methanogenesis from methylated compounds	Methanol:corrinoid methyltransferase
Membrane Transport	Membrane Transport	Ton and Tol transport systems	TonB-dependent receptor
Respiration	Electron accepting reactions	Terminal cytochrome_C oxidases	Cytochrome c oxidase polypeptide I (EC 1.9.3.1)
Respiration	ATP synthases	F0F1-type ATP synthase	ATP synthase alpha chain (EC 3.6.3.14)

**Table 2:** Timing of the microbiome measurements alongside their respective water quality metrics.

	Microbiome Measures		Water Quality Measures
Date	Code	Week Number	Week Number
**14-October-14**	4717_003	**42**	**43**
14-November-14	4717_008	46	46
17-December-14	4717_010	51	51
07-January-15	4717_012	2	2
22-January-15	4717_013	4	4
04-March-15	4717_014	10	10
02-April-15	4717_016	14	14
05-May-15	4717_018	19	19
28-May-15	4717_020	22	22
23-June-15	4717_022	26	26
21-July-15	4717_024	30	30
19-August-15	4717_026	34	34
**18-September-15**	4717_028	**38**	**39**
13-October-15	4717_030	42	42
11-November-15	4717_032	46	46
**25-November-15**	4717_033	**48**	**49**
23-December-15	4717_035	52	52
14-January-16	4717_036	3	3
03-February-16	4717_037	6	6
**09-March-16**	4717_038	**11**	**12**
**06-April-16**	4717_040	**15**	**16**
20-April-16	4717_041	17	17
**18-May-16**	4717_042	**21**	**22**
**01-June-16**	4717_043	**23**	**24**
29-June-16	4717_044	27	27
20-July-16	4717_047	30	30
18-August-16	4717_051	34	34
**12-September-16**	4717_054	**38**	**39**
12-October-16	4717_058	42	42
17-November-16	4717_081	47	47
22-December-16	4717_082	52	52
09-February-17	4717_083	6	6

## Data Availability

The 64 raw, unprocessed Illumina sequencing reads (fastq files) for all metatranscriptomes from the pair-end sequencing of the 32 samples have been deposited in the National Center for Biotechnology Information (NCBI) database under the Bioproject ID PRJNA1122484 [62] (http://identifiers.org/ncbi/BioProject:PRJNA1122484 (accessed on 25 October 2026)) and Sequence Read Archive (SRA) project accession number SRP513109 [63] (http://identifiers.org/ncbi/insdc.sra:SRP513109 (accessed on 25 October 2026)). The process data was uploaded to figshare [64] (https://doi.org/10.6084/m9.figshare.27073612.v1). The detailed outputs from FastQC and MultiQC are also made available online [34]. The detailed output of SAMSA2 is made available online [47]. The data descriptor paper, published in Scientific Data (Nature), provides a comprehensive account of the sampling campaign and the technical validation of the dataset. It is available at https://doi.org/10.1038/s41597-025-04682-w. FastQC [35] (v0.12.1, https://github.com/s-andrews/FastQC (accessed on 25 October 2026)) was used to conduct a quality assessment of the raw data. MultiQC [36] (v1.22.2, https://multiqc.info/ (accessed on 25 October 2026)) was used to aggregate and summarize results from FastQC. Groningen Microbiome Hub data analysis pipeline (https://github.com/GRONINGEN-MICROBIOME-CENTRE/GMH_MGS_pipeline (accessed on 25 October 2026)) can be used for total community profiling. SAMSA2 [42] (v.2.2.0, https://github.com/transcript/samsa2 (accessed on 25 October 2026)) pipeline was used to predict microbial functions encoded in the meta-transcriptomes. The data analysis was performed using SAMSA2 master analysis script modified to use UMCG HPC cluster (https://github.com/GRONINGEN-MICROBIOME-CENTRE/GMH_MGS_pipeline/blob/main/utils/metatranscriptomics/SAMSA2_master_script_RUG_habrok.sh (accessed on 25 October 2026)). DIVE package can be found at: https://github.com/AMahajna/dive (accessed on 25 October 2026).

## Supplementary Materials

The following supporting information can be downloaded at: https://www.mdpi.com/article/10.3390/biology15212047/s1, Table S1: Alpha Diversity Metrics; Table S2: Sequence Quality Control Summary; Figure S1: Krona Interactive Plots.
