# Supplementary material for: DIVE: Diversity-Informed Valuation of Ecosystem Functioning
Source: Biology (Basel). 2026 Nov 9;15(21):2047. doi: 10.3390/biology15212047 (PMC13762180; doi:10.3390/biology15212047)
Supplement: Supplementary file 1 [file biology-15-02047-s001.zip › Figure S1_Krona_pie_chart_on_Kraken2_and_Bracken_output__html.html]

Javascript must be enabled to view this page.

magnitude
magnitudeUnassigned

4717\_003\_03\_L001\_001
4717\_008\_S1\_L001\_001
4717\_010\_S2\_L001\_001
4717\_012\_S3\_L001\_001
4717\_013\_01\_S1\_L001\_001
4717\_014\_2\_S1\_L001\_001
4717\_016\_S4\_L001\_001
4717\_018\_03\_L001\_001
4717\_020\_S5\_L001\_001
4717\_022\_S6\_L001\_001
4717\_024\_S7\_L001\_001
4717\_026\_S8\_L001\_001
4717\_028\_01\_S2\_L001\_001
4717\_030\_3\_S2\_L001\_001
4717\_032\_S9\_L001\_001
4717\_033\_S10\_L001\_001
4717\_035\_03\_L001\_001
4717\_036\_S11\_L001\_001
4717\_037\_S12\_L001\_001
4717\_038\_01\_S3\_L001\_001
4717\_040\_2RNA\_S1\_L001\_001
4717\_041\_S14\_L001\_001
4717\_042\_03\_L001\_001
4717\_043\_S15\_L001\_001
4717\_044\_2\_S3\_L001\_001
4717\_047\_S16\_L001\_001
4717\_051\_S17\_L001\_001
4717\_054\_01\_S4\_L001\_001
4717\_058\_1\_S4\_L001\_001
4717\_081\_S18\_L001\_001
4717\_082\_01\_S9\_L001\_001
4717\_083\_S19\_L001\_001
4717\_003\_03\_L001\_001
4717\_008\_S1\_L001\_001
4717\_010\_S2\_L001\_001
4717\_012\_S3\_L001\_001
4717\_013\_01\_S1\_L001\_001
4717\_014\_2\_S1\_L001\_001
4717\_016\_S4\_L001\_001
4717\_018\_03\_L001\_001
4717\_020\_S5\_L001\_001
4717\_022\_S6\_L001\_001
4717\_024\_S7\_L001\_001
4717\_026\_S8\_L001\_001
4717\_028\_01\_S2\_L001\_001
4717\_030\_3\_S2\_L001\_001
4717\_032\_S9\_L001\_001
4717\_033\_S10\_L001\_001
4717\_035\_03\_L001\_001
4717\_036\_S11\_L001\_001
4717\_037\_S12\_L001\_001
4717\_038\_01\_S3\_L001\_001
4717\_040\_2RNA\_S1\_L001\_001
4717\_041\_S14\_L001\_001
4717\_042\_03\_L001\_001
4717\_043\_S15\_L001\_001
4717\_044\_2\_S3\_L001\_001
4717\_047\_S16\_L001\_001
4717\_051\_S17\_L001\_001
4717\_054\_01\_S4\_L001\_001
4717\_058\_1\_S4\_L001\_001
4717\_081\_S18\_L001\_001
4717\_082\_01\_S9\_L001\_001
4717\_083\_S19\_L001\_001

532906652066784630489437185417402072152522897821327541419580500473355900190302263147741933693813690642624508643045281132643876407042995349488006239159791175146556219528147689569734449418483690768304939435105056273121730265470688026578310501684619725509466435554600392418354587539390939788168339628724627742693212947210585767971277833744662410936989161199874281840265916761863581199575835

0000001000000000000000000000
02244113013111151125330310430101323

0
0

0
0

0
0

0

01751
0000

0000
01751

0000
01751

0000
01751

0711
01751

24

8

000
210

210
000

21
00

1
0

1
0

1

0
2

0
2

2

0

2

0000100
12151300

00010
121530

00000
121430

21430
01030

1

01
23

22

0
1

1
0

1

0
0

0
0

0

74217136113120133103101123
00000000000000000000000

02000000000000000000000
74217136113120133103101123

41211231011
21010020000

21120011
22121111

111

3
0

3

1000
1111

11

1

11
00

01
11

1

0
2

0
2

2

21
00

1
0

1

0
2

2

111
000

101
111

1

1

3
0

3
0

3

2
0

2

31111321112
31111321110

2

1

00
22

22

000000
810102

810102
000000

8112

2
0

2
0

0
2

2
0

2

014
00

00
014

14

1
0

1
0

1
0

0
1

0
1

1

200100
000000

200100
000000

200100
000000

000000
200100

000000
200100

21

499066015475905331264942000774751697472864511318416514541511343540041713483329174486577356814258529591943777518626732145004560289759463316650406118495873491177912519584738545431186875169815306444616310644289349992927848773737281441853157194577758364403262
00000000000000000000000000000832837331810836313124933531874771009929472513653133841

000000000000110050000000000001000000
2401701332634953155513776313221510226133149321331353

00
11

11

00000000000001000010000000000000000
240170133263495315543726313221510226133149311221353

011371111573211312
0000000000000

0
1

0
1

1

00
61

61
00

61

0113111573211312
0010047308710

00000000
011311002262

1131102262

0
1

1

2401720262785314542715312221510224136301021353
0000001000210002225920000811100001

00000000000000
240024221132110314

00000000000000
240024221132110314

2424221132110314

000000000010000200000021000001
1720263551332713110156722411816921212

11311
00000

11311

000000000000000000000000000000
172026245133261317126722411515921211

172026245133261317126722411515921211

0
1

0
1

1

00000000000000000000000000004433864562239020100111814845464674112174941416656137443527340057525167816453133746
498826015475905331264942000774751697472864509618416514541511343540417134700291484786373568142585295919437775186267321449899602227590133118499549183706833791789351903473374535618653516111529744379910614286347192921247994736631437553091194387753964293258

016232193351211133143051136
00000000000000000000000

016232193351211133143051136
00000000000000000000000

00000000000000000000000
016232193351211133143051136

016232193351211133143051136
00200000000000000000000

16212193351211133143051136

73
00

73
00

73
00

73
00

73

49622601547580633126494200077205169747214450261841651454151134354041713439329148477617356814258529591943777518626732144924559745753673286148792192201526338773851283467984483818341510611510643451010234203341522890247490730001410852359192377697859563204
0000000000000000000000000000118894810144821910020160149167011389322491343297992784101950272312074772299482132920760

000000000000120000200100300100200100020
197127190106331614759186160183108881217251461539394221226312911530100806471314610

112453723211121
00000000000000

112453723211121
10215230024011

13131271

1111

117112223
000000000

323
523

2

1121122
0000000

1121122

00000000000100000000000111000298953602100
19712719010633161475918616018310687118714126234323155193271002710077647121429

201310101301122
511461511511263

21531111

11111131

00000000000000000000000002000000
1971271901061475918616018310086118694811421127521843963541369

81111211

19712719010614759186160183100861186944227501713963541369

111212913
000000000

111212913

1111
1101

1

21421
00100

11

1

231

33162916114314
0000000000

33162916114314

0000
1222

1222
0112

11

1

3137310002514870150846749232603238312441412102118912654414416457695274121742410
000000000000000000000000000000000000000000000000

3137310002514870150846749232603238312441412102118912654414416457695274121742410
000000000000000000000000000000000000000000000000

3137310002514870150846749232603238312441412102118912654414416457695274121742410
0000000000000000000324001013000001000001140302000

1

1467431116112311910313

3

313731251487158453492326232825173131961518812654212416144593873521442110

1122
0000

1122
0000

0000
1122

122

1

0000000000000000000000000000000000000000000000000000000000
493945999075585330204942076955166447200449391841651423151134354041713416229022477617333314258527761943777309626732144791758670742003228346792052039221475624956545644438191803649678148054227310094103329492831846658716721362349994187387547957493124

493945999075585330204942076955166447200449391841651423151134354041713416229022477617333314258527761943777309626732144791758670742003228346792052039221475624956545644438191803649678148054227310094103329492831846658716721362349994187387547957493124
000000000000000000000000007576961013986564911394138165256013100778411939418610217469114440140996671267819049206573728111722785152631627589

0000064241185207951194852237452143
15526622219420394442111024013915761712108389572243

1552251791942031211717112316679346

41431611221412171261

1251111

1481224136781817373108221827841121651414212962125951611445437416957824110336
000000000191420721815611136117611059315104182615495158167016

111151

111

788432131111364820211

11

1481227312382328151231115

12

1121

1

731081111822251

41369712111132123116973

1752444622855693864434204515428651929830053016411311218014236122637526323416221439726927216426173481
0000000000000000400101210202018111011023142216336190

70418382111149316314115221

175244462285569386443420451542164782983053041379139351202348249223161111480341981611514337

214161132241
000000000000

214161132241

111101222
114111322

3

11

1886981154919826718066337338943199110914026303131
00000000020000010201002111

188698115491982671806613733894319901071392628302

4935859969755413300149420752050929461904253917798504951464439340417134025288144708272407139605253619298766436267321440261490166012926606353015316582748523598737151304691338438820976926052865309826086213693707550516965538581158415973441092529
000000000000000000000000003241138568451362134627518213552539242727124944245931006728823755922391667230620779172532323142448741629370101284648229441889

365014820554773261033753631345347413743751116387453621326733483891224801675810248359421183439579130266428345602656650990081219744956552126328177826108045283304291212033325186731403628304102491519837618435411685943563

438871758462321221312184089444790924947346423766623576130812173736180374347185113314589652731743847571329179159143180762901844841216

11352812017156733810041975755781066782211117626204945335014270669111731061

12419108931989764361157108537422856396396436571128586280107341058836400300441813120905964110761670522115310642428579112517935641230097398973192910429859529230416912861172107320460

36214419511850235728271635132151114361341154401215
000000000000000000000000000000

36214419511850235728271635132151114361341154401215

12
00

00
12

00
12

0
2

2

1

91
00

91
00

91
00

00
91

31

6

6143251021531485114499258
10010000101000000112810

000
121

0
1

0
1

1

00
21

21

3131145121474111111157
000000000000000000000

3131145121474111111157
000000000000000000000

312114512147411111157

1
0

1

1
0

1

000102100100
111115411226

2
0

2
0

2

0
1

0
1

1

2112
2100

0
2

2

0
1

1

0
1

0
1

1

0000000
1112114

21
01

2

00000
11114

1114

1

111411212121
000000000000

111411212121
000410002110

11
01

1
0

1

1
0

1

11121
10000

0
1

1

112
100

1

2

000000000000000000000000000000000000000000
26099277270001451020192907628132113534552871575810394483926242489632978616422428728

000000000010000000000000002000000000010000
26099277270001451020192907628132113534552871575810394483926242489632978616422428728

0000000000342215310043103817424245127110116513184114841
26099277270001451020191907628132113533552471555810394463926242489529978516412427728

462111624322122181364163
14110031322202202342140

1111

11133

61

1121

11

21

2112131

570281514108121159132910321883977311134225111712454
002498789113813289231563864388141615611254

11

3331311131113

111

11133122

572123212861117

21111

14

100
111

11

0000000007331338110101116626111620351274731388722611117
26099277270001454512547471338119253085825414552302381464601129361118977

2229972145253571134658187197911104521511093335

38277020179322313962147618876213721252451

0000000
1421311

0000000
1421311

1421311

7161462123311
000000000

7161462123311
000000000

7161462123311
000000000

000000000
7161462123311

000000000
7161462123311

7161462123311

0010100
1114212

1
0

1

11411
00000

0000
1411

0000
1411

141
000

1

14

1

1
0

1
0

0
1

1

0
1

1

0000000000000000
782115612131974683

782115612131974683
0000000000000000

782115612131974683
0000000000000000

0000000000000000
782115612131974683

0000000000000000
782115612131974683

782115612131974683

000000000000000
2428226361130225132

2428226361130225132
1820032000000000

015
00

15

1422
0000

1422

000000000000
23201341131112

23201341131112
16190231111001

711211
000000

711211

1

1
0

1

0
1

1

3221
0000

3221
0000

0000
3221

3221

00000000000000000000000000000000187156377260819481145684456031827710116610791041565314551210479461428312136107564797481683330211648633853399418848220378379143196722959
55839264452202878318479122840036511617386162133481132342186844815483136561698925916412222115501406425199248576043627533157034278747484954482722957638711639742525159691760836467273321087255975215389816322988289121123432332912542650049540288605612175394720059170344059517968715421294834636219975153194042510224

000000000
213164111

213164111
000000000

213143110
213164111

11

2

00000000000000000000000000000000114120004524644253895997218783553101263391875937141344306516534521516756813925492466191597371290105818461159
719537528842379011084705122315578540107136813267934523201541577864368921528338071866160831617176979534324411560436112959208527853213744773346931970324536425685462457765842354136325225269345812115628791304105121072111811339322914902247457781662

607417664872084289036043723141209283201095605352413783278460520071480832132123589621245112040723168093364110778149676115962114780630831566272013356234227988191503623320190346210514282522372512990814949162083212
00000000000000000000000000000000000000000000000000000000000000

00000000000000000000000000000016131371913133130111242480415300154421
607417664872084289036043723141209283201095605352413783278460520071480832132123589621245112040723168093364110778149676115962114780630831566272013356234227988191503623320190346210514282522372512990814949162083212

247753719015762963922814753181455444121046346766235723622017382386022351941569525961910559301361315523514719109719156013835530100128361317920525115966780127073229115
0000000000000000000000000000000000000000000000000000000000

247753719015762963922814753181455444121046346766235723622017382386022351941569525961910559301361315523514719109719156013835530100128361317920525115966780127073229115
0000000000000000000000000000000000000000000000000000000000

247753719015762963922814753181455444121046346766235723622017382386022351941569525961910559301361315523514719109719156013835530100128361317920525115966780127073229115

35971229297192665940512092312645610175551193247810322512224817711260832132123572241007518172375239810458868937444122468555521599146485121114117388632159399493280169335195121822412216621232201801790196
000000000000000000000000000000132883486279208417013613956931197240359781122125600368715227410946449172

00000000000000000000000000000000000000000000000000000000000000
359712292971926659405120923126456101755511932457103225122248177112608321321235722410075181723752398104588689373093312769273138914231587528781730861155129197563139906181883514048108133133924

359712292971926659405120923126456101755511932457103225122248177112608321321235722410075181723752398104588689373093312769273138914231587528781730861155129197563139906181883514048108133133924

2131213145111211
00000000000000

2131213145111211

23411
00000

23411

000000000000000000000000000000155285605383733222191493314477487815750950531022761301598634829202328264610106
11211986397294821811007514585401434823118899963787932591682672167563171213726497912792834677202181589883105423019421349702117113719310363203170773458675158114855697114251851593284156910564621185001849291

000000000000000000000000000006110111183607602111179911766420521586102109947401045
7707782289941302148178131365122103198183303224445524633711823278234779906399745151275340510906224644441864051731126289186110137678180852302992951140345440035127773564

0000000000000000000000000000000000000000000000
2284644270991595716991129346302283266583221158121810340764422291506676165121231091418443533171485

2284644270991595716991129346302283266583221158121810340764422291506676165121231091418443533171485
00000000000000000012323130002211749435440007061059830

773121441

22846442709915957169911293462252832665832211571013101346344227331533110612388141234302091445

22

000000000000000000000000000000000000000000000000000000000000
5427781829501032138262231365651032992174189845524633711820255431579905739165159533403347239217333342625317257294327356049610242222766231112284026627122048354

000000000000000000000000000000451059530500413201220104440113218016255426
5427781829501032138262231365651032992174189845524633711820255431579905739165159533403347239217333342625317257294327356049610242222766231112284026627122048354

7477818291891313195763136565103299217418723362003371181776382957990573893483916340296722190212260180112162240151737373702422265281684263925725519440526

75796814412733332710101314

3933211963462640461811620233237291181660223206119431218141111210231120202

0000000000000000000000000000000000000000000000000000000000000
351120816919548798594677227784810985801454576101512274261338513479429030237289249320590624921867910811585326281954112739379474539270381394800174383635926322142276312231485159504121

351120816919548798594677227784810985801454576101512274261338513479429030237289249320590624921867910811585326281954112739379474539270381394800174383635926322142276312231485159504121
0000000000000000000000000000000000000000000000000000000000000

351120816919548798594677227784810985801454576101512274261338513479429030237289249320590624921867910811585326281954112739379474539270381394800174383635926322142276312231485159504121
00000000000000000000000000000130552779394065090641710011197615170121272258649152374329518321571635158010839441466

31141115

4422001274124624321152521712

1212

111522

16625844416272242326495311142302681871153010020321227028715925341133181245

5985130123321233281112117717214

2191229191103662935092712062882442491015677151253711330261116221912044

17478512029027149191412152159214217219

1242114

695873522713114713242113492

185601477774356525253227784810926459882831077184261338242232029018837821685205478710126751040502219101116256316626106187540581504412553395496

3886185511

55173053165171111522130011
00000000000000000000

55173053165171111522130011
00000000000000000000

55173053165171111522130011
00000000000000000000

55173053165171111522130011
00000000000000000000

5517305316517111152213011

0000000000000000000000000000000
40442012842763293143412531112261311165

40442012842763293143412531112261311165
0000000000000000000000000000000

40442012842763293143412531112261311165
0000000000000000000000000000000

4044212842763293143412531112261311165

886890329310375133811860112884529756210348221952981383284609115685537302519651268163271022153154510648810913026414511325288991910410218662103482071344351254353912927124161172441532121
0000000000000000000000000040931839803254038787267896194533101958182317800427710123313418843213

73261962937848116211705912868429721166891353431287128460911568553721081965124725328880138963762040255450255105196102594266711529314311434452538223273689811461111986
00000000000000000000000000000000000006401100000002409410

3048835098133512612352101043433331511265125930260247171514981391225184281271186153148830111314413
000000000000000000000000000000000000000000000

3048835098133512612352101043433331511265125930260247171514981391225184281271186153148830111314413
000000000000000000000000000000000000000000000

3048835098133512612352101043433331511265125930260247171514981391225184281271186153148830111314413

0000000000000000000000000000000000000000000000000000000
702219620543391080817059128649297855454114342085282516091153705379821964135026880138363521323104401247105183115711479872807196284510357519438591281061973

702219620543391080817059128649297855454114342085282516091153705379821964135026880138363521323104401247105183115711479872807196284510357519438591281061973
0000000000000000000000000000000000000000000000000000000

702219620543391080817059128649297855454114342085282516091153705379821964135026880138363521323104401247105183115711479872807196284510357519438591281061973

0000000000000000000000000000000000000000000
154270725271760154216135136598669865129172099991421419412693336121301271803737413161391233181410122

154270725271760154216135136598669865129172099991421419412693336121301271803737413161391233181410122
0000000000000000000000000000000000000000000

0000000000000000000000000000000000000000000
154270725271760154216135136598669865129172099991421419412693336121301271803737413161391233181410122

154270725271760154216135136598669865129172099991421419412693336121301271803737413161391233181410122

00000000000000000000000000000000000000000000000000000000
5006141211526191578377610310504624853719803310872576530712716712410035838786100182722652371441117872216133037907917211631537321146498741204483219525761305728687195101028768021967152581051941995499

00000000000000000000000000000000000000000000000000000000
5006141211526191578377610310504624853719803310872576530712716712410035838786100182722652371441117872216133037907917211631537321146498741204483219525761305728687195101028768021967152581051941995499

5006141211526191578377610310504624853719803310872576530712716712410035838786100182722652371441117872216133037907917211631537321146498741204483219525761305728687195101028768021967152581051941995499
00000000000000000000000000000000000000000000000000000000

00000000000000000000000016105983550169699413934910711148276017833590616597987926154684527705134355320063393303265489192
5006141211526191578377610310504624853719803310872576530712716712410035838786100182722652371441117872216133037907917211631537321146498741204483219525761305728687195101028768021967152581051941995499

41829659589722445208243866346168372404620922130821615218651953146331245327769172335631939602376553516874114126284363128301980616161124618123829411446038652284823651011321273
00000000000000000031166686887457193930836253351630381372600420312208187961543612271720370240314453794228432263941245259

1

2

72290511290131221411215

1

4182965958944520824386634616837240462001713082161521865195314633124516123345641425642715762210087091892127831570317769

8244471937191561331101023023821912966704148431763555190635163073228005822263501521004123704966710318510238788145325911674265211596854619561611582818534
00000000000000000000000000000000000000000000000000000000

8244471937191561331101023238219129667041484317635551906351630732285822263501521004123704966710318510238788145325911674265211596854619561611582818534

0000000000000000000
086429011239121812179415142

086429011239121812179415142
0000000000000000000

086429011239121812179415142
0000000000000000000

0000000000000000000
086429011239121812179415142

8642911239121812179415142

0000000000000000000000000000000000000000000000000000000000000000
19714148997511224901258841587619287173143192194948619164179618968149122459911549818355201102269119724220551570011266402167212868151641461622832131111016108910575503695825353610544982309360044601831720115715091092382061693061607771759371376411883201334386145621034702

0000000000000000000000000000000000000000000000000000000000000000
19714148997511224901258841587619287173143192194948619164179618968149122459911549818355201102269119724220551570011266402167212868151641461622832131111016108910575503695825353610544982309360044601831720115715091092382061693061607771759371376411883201334386145621034702

0000000000000000000000000000000000000000000000000000000000000000
19714148997511224901258841587619287173143192194948619164179618968149122459911549818355201102269119724220551570011266402167212868151641461622832131111016108910575503695825353610544982309360044601831720115715091092382061693061607771759371376411883201334386145621034702

19714148997511224901258841587619287173143192194948619164179618968149122459911549818355201102269119724220551570011266402167212868151641461622832131111016108910575503695825353610544982309360044601831720115715091092382061693061607771759371376411883201334386145621034702
0000000000000000000000000000000000000000000000000000000000000000

0000000000000000000000000000000000000000000000000000000000000000
19714148997511224901258841587619287173143192194948619164179618968149122459911549818355201102269119724220551570011266402167212868151641461622832131111016108910575503695825353610544982309360044601831720115715091092382061693061607771759371376411883201334386145621034702

19714148997511224901258841587619287173143192194948619164179618968149122459911549818355201102269119724220551570011266402167212868151641461622832131111016108910575503695825353610544982309360044601831720115715091092382061693061607771759371376411883201334386145621034702

1355158467112113827511139334591832963644211211104187174736
00000000000000000000000000000000

1355158467112113827511139334591832963644211211104187174736
00000000000000000000000000000000

00009942741112927947083296354421121197187164736
1355158467112113827511139334591832963644211211104187174736

135515846711214411055121171

183753748079714324949836246341230514694336307103233094331332512417521424213702374310915233443588771516546595547258581105224204291225742071047805158341198516483685931247538010774742150136
0000000000000000000000000327112802076017222624221215737671523720135932732251165641345135801123371901811332112

23037434102341716302903011431131961313517192
000000000000000000000000000000

23037434102341716302903011431131961313517192
0111010150101551682121100372100116000

24127152117571101113264
0000000000000000000

2412615211544131113254
24127152117571101113264

12171

12

0000000001100000120002000
23023213742123542211141225152

211
000

211

0110310203011301012040
23022137422254221121213152

11

1

5111

11111

11171

230142211112

0000000000000000000052451523131241114118952444795810647031328571270103421221
15372575291002418646412622389246344455005806366941427911337420497510657232445135674911162838143120198118882492145617349612332132156189641288

0000000000000000000153127253719012326230084482111825300065724
153725741797439774124017622396435227016418356714279113374204912574116941812701299137321027597081370139747365492362212444339977

11
00

00
11

11

1434417974379541165137923964352270164582521424211381651130370121313511857216931316119631212612320242597
000000000000000000000000000000000000000000

000000000000000000000000000000000000000000
1434417974379541165137923964352270164582521424211381651130370121313511857216921316119621211612320242597

1434417974379541165137923964352270164582521424211381651130370121313511857216921316119621211612320242597

000
111

111

25775383374204411912775616143214101421162295622133
0000000000000000000000000000000

00000264252001000032022900000000
25775383374204411912775616143214101421162295622133

113212111

11132

75204211213236141313

112

5111

13

71

1

4

2573833745102114612911752293182

00000
8911012

00000
8911012

8911012

103931253153740216423833666281141108315623
0000000000000000000000000000

103931253153740216423833665281141108315623
0000000000000000000000000000

103931253153740216423833665281141108315623

0
1

1

38149372111812261021168
0000000000000000

38149372111812261021168
0000000000000000

0000000000010000
38149372111812261021168

38149372111812261011158

1

000000000000000000000000000
704535111111624102699141411122022221

000000000000000000000000000
704535111111624102699141411122022221

704535111111624102699141411122022221
0000000010420050000011300010

704532825311371211

3511111121631141111111

000000000000100000000001001000110000000
11228209462251967933665416127103391179212126512303192812201731370134631

000000000000000000000000000000000000000
112282094622519679336654161279339117921212651130319261220170970134631

112282094622519679336654161279339117921212651130319261220170970134631
000000000000000000000000000000000000000

112282094622519679336654161279339117921212651130319261220170970134631

123
103

2
0

2

0000000000000000000
2763311483311151211437

2763311483311151211437
0000000000000000000

0000000000000000000
2763311483311151211437

2763311483311151211437
0000000000000000000

2763311483311151211437

13796650163185985024973454139883110529462213333181541
0000000000001110000261000210061110010

137966501631859850237221239584952711241151431
00000000000000000000000000000

137966501631859850237221239584952711241151431
00000000000000000000000000000

137966501631859850237221239584952711241151431
00000000000000000000000000000

137966501631859850237221239584952711241151431

1251323312431231131
0000000000000000000

1251323312431231131
1141020312400200021

0
1

1

331
000

331

000000
113311

113311

00000000000000000000000000000000000000000000000000000
1631844807442332290797624172524554517249589436331322312415834232262629117952014224393356502242208166019224284159104042304713513971595173782725574220360514

1631844807442332290797624172524554517249589436331322312415834232262629117952014224393356502242208166019224284159104042304713513971595173782725574220360514
00000000000000000000029577111115951216947919146703910877931310342632714431815216152703

40962036630511401941442317112812522733218271
0000000000000000000000000

0000000000000000000000000
40962036630511401941442317112812522733218271

40962036630511401941442317112812522733218271

448348411197651103231316231214144
00000000000000000000

00000000000000000000
448348411197651103231316231214144

448348411197651103231316231214144

831847157562500904926215347248731663137824981613315332321722757232559148156628343041241771646612164324853
00000000000000001241541215211491251531414913712289150232216611

000000000000
83154161115221122114

8315416111522122114

1

000110
111111

1

111

11
00

11

0000000000000000000000000000000000
18457562500904926215324816631334049811052241582726181082314517145536119132

18457562500904926215324816631334049811052241582726181082314517145536119132

47736221421279314613165
0000000000000000000

47736121179314512155

21141111

010000000000
71211121111111

711111111111

111

000
212

212

0
1

1

000203
112417

12

1

1

121

11

00
72

72

0000000
2881331116

2881331116

000000000000061448131070331113919963216821256420102481377711618039314500
80261205404751711075001977462412235662273371252227064574796328166225933872826489721041696911075171994261562396

0000000000000000000
2056761972231117126023116211124

2056761972231117126023116211124

000000000000000000000000000000000
80271651711075004262415661343351322916711325791272506311441

80271651711075004262415661343351322916711325791272506311441

2616553202273177411646047031563641181424762761572395
0000744637532543124249744017426251805

2612991378542041812351411155123

3563208955317318221122126135136

195264159321981191861954119121671125425211131117106112
0000000000000000000000000000000

0000000000000000000000000000000
195264159321981191861954119121671125425211131117106112

195264159321981191861954119121671125425211131117106112

54213111
00000000

00000000
54213111

54213111

618584415684421621212112
0022100100010000000

111
000

111

6181448142
0000000

6181448142

010201
121241

1114

00000000
5821211212

5821211212

0000000000000000000000000000000
936199265143851542104104103759112135621513231738

0000000000000000000000000000000
936199265143851542104104103759112135621513231738

0000000000000000000000000000000
936199265143851542104104103759112135621513231738

000720250040411111002063002231032
936199265143851542104104103759112135621513231738

9361582651145459145611171171474

41643542613122112223462

8069477113341015233862825072617071205119162479218389041997245295288453951128575725061262810794913610369251421177668778263712907897569319382494168483188753593401913633338860461162657726681252551157674086753521784621457610005834168
00000000000000000000000000000000271718133243335778169831732815941922564911831145192146033191048241953900310523297422167249316865094

5781633777147315131463598621594131810732553423572477935612739128553342407953615836532802364733603757313624446459182347244438021244246792134208136584281781145592130149228233211010392051038591498623
000000000000000000000000000000000000000000000000000000000000000

000000000000000000000000000000000000000000000000000000000000000
5781633777147315131463598621594131810732553423572477935612739128553342407953615836532802364733603757313624446459182347244438021244246792134208136584281781145592130149228233211010392051038591498623

5781633777147315131463598621594131810732553423572477935612739128553342407953615836532802364733603757313624446459182347244438021244246792134208136584281781145592130149228233211010392051038591498623
000000000000000000000000000000000000000000000000000000000000000

5781633777147315131463598621594131810732553423572477935612739128553342407953615836532802364733603757313624446459182347244438021244246792134208136584281781145592130149228233211010392051038591498623
000000000000000000000000000000000000000000000000000000000000000

5781633777147315131463598621594131810732553423572477935612739128553342407953615836532802364733603757313624446459182347244438021244246792134208136584281781145592130149228233211010392051038591498623

00000000000000000000000000000000000000000000000000000000000000
22884138557867918732678712810861146781621611116357761521217352841212520421648549543333504122778444650725518850109176733774866717027210333863316162467274147716594685272021111145323249104524119851

00000000000000000000000000000000000000000000000000000000000000
22884138557867918732678712810861146781621611116357761521217352841212520421648549543333504122778444650725518850109176733774866717027210333863316162467274147716594685272021111145323249104524119851

22884138557867918732678712810861146781621611116357761521217352841212520421648549543333504122778444650725518850109176733774866717027210333863316162467274147716594685272021111145323249104524119851
00000000000000000000000000000000000000000000000000000000000000

00000000000000000000000000000000000000000000000000000000000000
22884138557867918732678712810861146781621611116357761521217352841212520421648549543333504122778444650725518850109176733774866717027210333863316162467274147716594685272021111145323249104524119851

22884138557867918732678712810861146781621611116357761521217352841212520421648549543333504122778444650725518850109176733774866717027210333863316162467274147716594685272021111145323249104524119851

0000000000000000000000000000000000000000000000000000000000000
623002652122827292028269261221537903823001797191518910330013331860145696473711932974350558372024259123991123267196210163309265965914880412245219849441

0000000000000000000000000000000000000000000000000000000000000
623002652122827292028269261221537903823001797191518910330013331860145696473711932974350558372024259123991123267196210163309265965914880412245219849441

623002652122827292028269261221537903823001797191518910330013331860145696473711932974350558372024259123991123267196210163309265965914880412245219849441
0000000000000000000000000000000000000000000000000000000000000

0000000000000000000000000000000000000000000000000000000000000
623002652122827292028269261221537903823001797191518910330013331860145696473711932974350558372024259123991123267196210163309265965914880412245219849441

0000000000000000000000000000000000000000000000000000000000000
623002652122827292028269261221537903823001797191518910330013331860145696473711932974350558372024259123991123267196210163309265965914880412245219849441

623265212282729228269261221537938230017971915189103313331860145696473711932974350558372024259123991123267196210163309265965914880412245219849441

0000000000000000000000000001091362975232111073615257115524220554911710103633361
271317173014547511258545804687250583515610491305169968113313654109131035561072126244614667560271242081261084203382533762652386292487698190411528265471214901544831232131327257

180749583614788624024911960620115467977221271212531
0000000000000000000000000000000

0000000000000000000000000000000
180749583614788624024911960620115467977221271212531

180749583614788624024911960620115467977221271212531
0000000000000000100100000010100

180749583614788624024911960620115466976222612243
0000000000000000000000000000

180749583614788624024911960620115466976222612243

111
000

111

20368235220516106461011397072762953558113338460171393265951262264669918642648235171815404121328338529177105165396432813116724613032
000000000000000000000000000001101001513110010000100045300

694946294964912417960171118427321893851291511491315177152682391285595612881461
00000000000000000000000000000000000000000

694946294964912417960171118427321893851291511491315177152682391285595612881461
00000000000000000000000000000000000000000

00000000000000000000000000000000000000000
694946294964912417960171118427321893851291511491315177152682391285595612881461

694946294964912417960171118427321893851291511491315177152682391285595612881461

000000000000000000000200301020000040001010000700010240
134681862205161067290581522953558113320539208168941175666113630558114654626212312307921147757410931971341628431

1346818622051610672901522953558113320539208168941175665213626558042546231951230791814675716211971281605831
000000000000000000000000000000000000000000000000000

1346818622051610672901522953558113320539208168941175665213626558042546231951230791814675716211971281605831
000000000000000000000000000000000000000000000000000

1346818622051610672901522953558113320539208168941175665213626558042546231951230791814675716211971281605831

000000000000000
58711232142342522

000000000000000
58711232142342522

58711232142342522

00000000000000000000000000000000000000000000000
29139823515465323300734962710832935322302071821387419532114914317125168312425543243141421631037713431406420613

29139823515465323300734962710832935322302071821387419532114914317125168312425543243141421631037713431406420613
00000000000000000000000000000000000000000000000

00000000000000000000000000000000000000000000000
29139823515465323300734962710832935322302071821387419532114914317125168312425543243141421631037713431406420613

0000000000000001022310981100101891490110229307388110131735612911125317260
29139823515465323300734962710832935322302071821387419532114914317125168312425543243141421631037713431406420613

21

291398235154653233007349627108329353223020718236519441339133163619314261255632302215228113353

000000000000000030003010000000
293522105746211103616581219326122033471189944

279522104521131232510321162714
0000000000000000000000

279522104521131232510321162714
0000000000000000000000

0000000000000000000000
279522104521131232510321162714

279522104521131232510321162714

00000000000000000000000000
14122518936145510141931217312712723

00000000000000000000000000
14122518936145510141931217312712723

0225008000452914163007312711500
14122518936145510141931217312712723

11111

141018136213129213

1191031258138808784121556424383209600448350642708023184131321075451821411522346456906364911123576816114024116
00000000000000000000000000000000000000000000000000

00000000000000000000000000000000000000000000000000
1191031258138808784121556424383209600448350642708023184131321075451821411522346456906364911123576816114024116

00000000000000000000000000000000000000000000000000
1191031258138808784121556424383209600448350642708023184131321075451821411522346456906364911123576816114024116

000000000000000000201261442000391924528686500198512981260
1191031258138808784121556424383209600448350642708023184131321075451821411522346456906364911123576816114024116

111131523

103138121556424383209148448270911931365111421721182018610511298

119125880878445235064801272241071510214102164371494371431416

278427308013267692295319144664483543112811144622132361129105139359108142503522271812166463191471689492776191383923472496860175638314824260682177082190591507766416414845220773116015894070

00000000000000000000000000000000901891221001073256580720899124591207610800057169944771103786808229774256991252958920177116749558035028033386484687615073983129248362025393135341929157341020854198161335
42716156546768669737799313256311806987406880435311328416099487239326285134124161030660259605505580024487174623190277068618677378014900080431299174799713329124824158074834254692593792804640793972855551606893163662611256171145509317288964932135041919349578933186813609215089565158859092855928449347861925628246060307537983388740543673017789772271212984615815778032461217374174468276

10111210311
000000000

000000000
10111210311

10111210311

933711141624771632998116746146363451771209681249913459134326111315247415629778160461403434116511786611698134364
0000000000000000000000000000000000000000000000000

933711141624771632998116746146363451771209681249913459134326111315247415629778160461403434116511786611698134364
0000000000000000000000000000000000000000000000000

0000000000000000000000000000000000000000000000000
933711141624771632998116746146363451771209681249913459134326111315247415629778160461403434116511786611698134364

933711141624771632998116746146363451771209681249913459134326111315247415629778160461403434116511786611698134364
0000000000000000000000000000000000000000000000000

0000000000000000000000000000000000000000000000000
933711141624771632998116746146363451771209681249913459134326111315247415629778160461403434116511786611698134364

933711141624771632998116746146363451771209681249913459134326111315247415629778160461403434116511786611698134364

391597685087270331651859231194301779934079754973536814504415683255149522478843322679273285756251395894949881434424858645343153630270911577613078861235035157842366216240875975254521731228013966758893530149311418113785814917242228775972214315848517178935246105428443814421014297841769133302235741515411332789664838552203
0000000000000000000000000000000095328164037136777652144306812122377193912845216574597394115283876399611722048199320052245108979931151689314361115492482

00000000000000000000000
30917123134121111514311263

00000000000000000000000
30917123134121111514311263

30917123134121111514311263
00000000120000001000200

000000000000
30731232122262

30731232122262

2141221111323181
0000000000000000

2141221111323181

31377699667538728682161271022244947886255123743138634339999937209517710470004196037697771116190644285135462241883510939710135351208668002914664956328153641628922547746287821021143518762200460562198733108301152843493101966069348615881839167595631041786639368300112282529721357181135051759775497786596643140532819
0000000000000000000000000000000000000000000000000000000000000000

31377699667538728682161271022244947886255123743138634339999937209517710470004196037697771116190644285135462241883510939710135351208668002914664956328153641628922547746287821021143518762200460562198733108301152843493101966069348615881839167595631041786639368300112282529721357181135051759775497786596643140532819
00000000000000000000000000000000478117523251307749352616180814786969415054122269584245044733099226549561727097288704873165361240874281153564765510067901936159725692744315634

283365934660900221011288490515263434718396265493193528899795018148509524248213495439234450015026281952042316551220993498710728215122676780324665826960245622236333985338141165092756511057249381053215342162841723158471366431568272509971297602793711019191211351111833143782672053337908449465667289312071414460
0000000000000000000000000000000059701105012012511341593005931209361348470641458272058554419110159364840173781871452001378393705268923910989305184442149123401637892769236

000000000000000000000000000000000000000000000000000000000000000
321109114227306151802616181214771371882419408305019929682936307099512761465137616627338419893363161217612951702184397512221338780146162851144234024926110697205161142923574389473778585442222434321987805815559

321109114227306151802616181214771371882419408305019929682936307099512761465137616627338419893363161217612951702184397512221338780146162851144234024926110697205161142923574389473778585442222434321987805815559

0000000000000000000000000000000000000000000000000000000000000000
222444566636621116311697117749311664651819641226112594515493245481008437522894213093554745001151202164154285814731058261155314276195401527942161042683755563324707125983016433501942191860220208182671091352872067666065179718238754282409199

222444566636621116311697117749311664651819641226112594515493245481008437522894213093554745001151202164154285814731058261155314276195401527942161042683755563324707125983016433501942191860220208182671091352872067666065179718238754282409199

337047287131294947253583401452160566217398420352610863012639103744904104591253966966947590071956466161532530940931947295912515126278121116113562562886186712712558951647176911515751080381639253425200935403465271620511496305318937324690012818401125365655412217
000000000000000000000000000000002846171545518657366725978519856532703811031234020039211972209595113847911322103524864375657313337202419711264

71299357206184158527345416170106774665440736955272493507657324102624853613253448225255285334034064221232810910211012511981557017549411142164918159

329944296774274345413583401293657835763382319652504863011865970944649723115846424645453936538614216153242833845189429591190212374767385568796234011579155289575672047629366727771476132097814431251168461263515568094834241458251178415413489894

0000000000000000000000000000000000000000000000000000000000000000
899245630501987279252242617539734019301713427489607375446123386498873734495313407425244613126614131227854547972843911496124337546224774713441543518516376204213319983654578105799496657149566463940313913541591269981216592

899245630501987279252242617539734019301713427489607375446123386498873734495313407425244613126614131227854547972843911496124337546224774713441543518516376204213319983654578105799496657149566463940313913541591269981216592

15025405126764804305537869010104489183183922202915797635229562110742771965517865346514350862949828858156843920737041054296014101851692255291267298796711221361783391972160677645560433744539459714264623302312962218154217831816679662398674141114281234571657
0000000000000000000000000000000000000000000000000000000000000000

15025405126764804305537869010104489183183922202915797635229562110742771965517865346514350862949828858156843920737041054296014101851692255291267298796711221361783391972160677645560433744539459714264623302312962218154217831816679662398674141114281234571657

0000000000000000000000000000000019122917210337261806715647463124320301933613633637826785244522208841736239144
304110620144876581324311792313539711610882669951001987280326152275226108330542661640381465615039763713010472328069574412351114182985855118541778326639341622155064335633217612712160516191013152367887789280310738629719743711409235643808252250681723667522504132482725

00000000000000000000000000000000000000000000000000000
41111166753660922522312222617728756538694315467251141183326093343303352120136181135108234937382156256939205711047

41111166753660922522312222617728756538694315467251141183326093343303352120136181135108234937382156256939205711047

17921821720452511051052551327717622244031251434048172014525769528469917332631231033035441831197478482508878248107454212715818011124812413035157934469537136894985613842973
000000000000000000000000000000221418762029316912101626162513714519315221551356273

2111351112112131

1011351379710533644147734255872921461091611856062114250130154869111365319211411949323718581622212431411325198517064644222301573880185332021727637

788380107405726618459359513514816639015729664383116422154545864152875011140964422116211724492293722156788453614235532812733239442631605455305831026

154324261522172723214661111234310318397111261431017855292167

0000000000000000000000000000000000000000000000000000000000000000
274999641394261362665117923118826699103046184468917392490251572666754383233425714316414230145196620123353843250305208100497317514507511309140127903531139012275131450031518231113501352805127760217117236010065563814793170363228143315198640771420458433432226062357

274999641394261362665117923118826699103046184468917392490251572666754383233425714316414230145196620123353843250305208100497317514507511309140127903531139012275131450031518231113501352805127760217117236010065563814793170363228143315198640771420458433432226062357

11111111
00000000

11111111

72327162166535162522311311035091329366128305356154281225253348181115220683383672252793411260473237336958642433263132152791391291059879163129113333814921148164104
00000000000000000000000000000000000000000000000000000000000000

72327162166535162522311311035091329366128305356154281225253348181115220683383672252793411260473237336958642433263132152791391291059879163129113333814921148164104

0000000000000000000000000000000000000000000000000000000
995217120720146227756583843223225687240417901830103211957395291151337018054256521337441572362848269138734182189185654208616921343826114655047112693

995217120720146227756583843223225687240417901830103211957395291151337018054256521337441572362848269138734182189185654208616921343826114655047112693
0000000000000000000000022251124041049123113117913102310912923547553195163137511142114

425211116211
000000000000

000000000000
425211116211

425211116211

722111119
000000000

000000000
722111119

722111119

76274528258481436318359181461142412931185623241414127217872569353
000000000034200538400011000100202251201110

000000000000000000000000000000000000
51274528242481066318359951120121111411121411123410472448333

51274528242481066318359951120121111411121411123410472448333

251637653117242116321221162281101
00011101000002000000101021

252611141411113217

16114422121431112121018

000000000000000
13141135111013452

000000000000000
13141135111013452

13141135111013452

00000000000000000000225324008420323623132117720252753164134384231318108117714
635274917756583843223225660234617621518984994676298565021284651839125836684839866126671771545731970161610206358404632415768

0000000000000648112145261347131341143277958227120910862819213632531338
67281231243722138166089443963058916456651222316349141024181655206361048833117151428469223376314914751

11122256

6778308168698718926311223281121573342126251118

28123466445267370543963058916430111152161433553823153116421616112923728233175

6352742456583843100101288190413674491674954221113212114233510201210443718081208814263211233
0000000000000000000000000000000000000000000000

6352742456583843100101288190413674491674954221113212114233510201210443718081208814263211233

00000000000000000000000000
49186125796197388011354541142910347939832021

49186125796197388011354541142910347939832021

000000000000000000000000000000000000000
3697402014622545848318298141321016301172121418231361710526661144128

000000000640040620211010913111031413223172365
3697402014622545848318298141321016301172121418231361710526661144128

31133521432

11317

3697402014622545848214288926716271421231341522341761

111111

112121

16111211213132
000000000000

16111211213132
000000000000

000000000000
16111211213132

16111211213132
000000000000

16111211213132

3157225022487935541150134622407137831649612845103844535346711171463119391184229815825522823212667779927231009127154197230316542138391230222859311077309439107911361934910794128417962750438492811758630401663127719401299406766381107361222303645818413476182011443118559115
0000000000000000000000000000000000000000000000000000000000000000

000000000000000000000000000000001066950172611987135085711635466941638142920514211122013322360294659020972514920389472476588
3157225022487935541150134622407137831649612845103844535346711171463119391184229815825522823212667779927231009127154197230316542138391230222859311077309439107911361934910794128417962750438492811758630401663127719401299406766381107361222303645818413476182011443118559115

19301764192567038038193414114896592951018779143896288685173322921229661587312437057313322215295799179532551628135541152592921099473744170203303691109325932562152254802313821754858169666133232030217042231570217811077221592524781146837993066046
00000000000000000000000000000000114527115213214211277636727126363081667492334950412715935066123913336116023488868775710

11846542113262
00000000000000

11846542113262

111231111
000000000

111231111

13311123161112
000000000000

13311123161112

000000000000000000000000
122229111311731171754212222251

122229111311731171754212222251

44467321881235142236303452474364016833111811053924710541256254
000002521762331452831501143281112192302148934183191

11167111

444673218812338572713121322456139331666363

0000000000000000000000000000000488222180587819939468133762355729782739117467396130113783102165111714503596114573413415231412650512951583965
18131697148358438038193413964893192711011879143051191654312162547526587300736776985322213925648179531781486134081110191908463172408411925854596005122487643854048198610751287395189101130234140717844677146285416936812109908734081705263

1111212131

1813169714835843803076678529660072338728705126751710522620185474314911979207733321874848411914412550130912251823944973418851223041951637997979116711667023442927031812411351144041006713310554922820082323464

13152101301114010441312213221342121

1

242221261258915111192141871

2211212

9392521213211648362141024127

2674032178416801233808310122205144959688514472494119240912462743741771022262743131217387382452371781226924241789121511917638117235154346725151580728

1111212221

132

7410344033581575566621431158981561352838025413519228282324118279827533122118647135292120533185115674306169

181327222121142111

121389098222471962213156103057229634

1503424371328155137284420336577337112191457281052323412979711163361426331
00000000000000000000000000000000000000000

1503424371328155137284420336577337112191457281052323412979711163361426331

211251253142132
11115933130110

11122

112111

000000000000
121316111711

121316111711

032201009240110050
5322351213331132152

2143221

5142612

1211221444211434
0000000000000000

1211221444211434

0000252112213236762125865124763011360411
56241093354261743197581120525112911644811665158372891

12211

1916221311131

11111

2517331161111

37241093310532931924313322251121114

131511

2111132151611

211111

61674428669752911233297233722913511764142978845615941067566442141859347208622110871325157120241157158343142324125937937
000000000000000000494736351516233984830601712182874825810510320101684027113222094814822

616744286697116612332972337229135117641429767451011431641758104991232771135848163103176211430112014

2142311171314211111

41362111211131446731131111

0000000000000000000000000000221315151124015620569921413652728276147731731781571812836428737061
2867694128293481872012640247063948226541309101895516379952398452753544577119442929209203932938231430102616562467365735870653999605621802005548472229411398608341400864214038054425375110173512767777368255320712481

000000000
111112222

111112222

0000000000000000000000000000191575334546063005863337453611931178745843520401003677697121070217403209361830419433122634647337215718312117
286769412829348187201264024706394822654130910189551637995239845275354457711944292920920393293823143010238645945686423476824384258512111191353345922291112832807133981620673632424535949993384731733360248019992418

13835841227212889249111051207221105580830155872137912912274164519065211270278313136378411492444651840350311190856424780695069614818922819123921893231616561131

14841154711929465415351197387482155250168039791661611072144341223996223022642581393157515301861213256165562151281914634129633923961621341673112392016658451515399164

1015666314737109297272512623224961941617513713274428125751577102586

000000000000000000000000000
9414863231231741899222825081932422176137261113835618217392

9414863231231741899222825081932422176137261113835618217392
000000000000000000000000000

9414863231231741899222825081932422176137261113835618217392

12012121823162761332211117191181212418233415827766013335211111698192
00000000000000000000000000000000000000

12012121823162761332211117191181212418233415827766013335211111698192
00000000000000000000000000000000000000

00000000000000000000000000000000000000
12012121823162761332211117191181212418233415827766013335211111698192

00000000000000000000000000000000000000
12012121823162761332211117191181212418233415827766013335211111698192

12012121823162761332211117191181212418233415827766013335211111698192

000000000000000000000
1833221122111182914732511

1833221122111182914732511
000000000000000000000

000000000000000000000
1833221122111182914732511

000000000000000000000
1833221122111182914732511

1833221122111182914732511

4376457094523690189394750674701502102620406611162101969494208106081451312304445010353596258183274103458112467217989040963563912441773795777826961695786479716012659281615601113699961303573978912345984941898611498154452792912391121332094840375534287692
000000000000000000000000000000001473164231974529619760851624328224802052743034039828118120517923432191901922844229

421241959292350518939475067470150295319076121123101969494208105251451312304445010353596258183274103457182467214789040503563912439173339728024331640755473693611908171417538107596656283354947412029944240818326479852072600882344119301975816350633797452
0000000000000000000000000000000023031594330499752029610419073543234872114635282517169834334566326711362592230020201252432105582497257816149462179

000000000000000000000000000000000000000000000000000000100000000
15251127168855827481852124184381188220261196112071585178311036037569731168932469131749058325696545256459860283428618745195115912821012412012097050682689260738053341760336818055522631186488301342

15251127168855827481852124184381188220261196112071585178311036037569731168932469131749058325696545256459760283428618745195075912821012411911997050582588860638053241760236618055422331186488301342
0000000000000000000000000000000188442589209105251520032911468934126742963265224511884402502232062135113313419299100238

71323782714540141814661746561583926252143428330236941471372685719034125191112567138157998719818542182073105424162445951412

11510311111251110173118136149432122284

3541681437541115186812566112767749601069629277935127113924869228105134118218122132623

225937348425102141322119828331311312239

14052860928523481455124184300188133234103468958874275252653039655752346998224016917743945247336546526471316623233724407012798150116811414853154761411243253296817275

11221261124445

203194109128562719553137121898383132971645472791523467372223941281231510431354341991519532474113301739610

1411114111113
0000000000000

1411114111113

00000000000000000000000000000000000000000000000000000000000000
243121822025843332716891794491922763162865180119712375151349178911991440138816111928571200492180716249789477874233415061844075104219961421031153623972125383831757952669257658504338475104787104325

000000000000000000000000000000516496425221684122365073126519048730378467906623202494381349388272751762125449556245
243121822025843332716891794491922763162865180119712375151349178911991440138816111928571200492180716249789477874233415061844075104219961421031153623972125383831757952669257658504338475104787104325

588100108862921292551051228118399485390220114189484196249212249932931138613143221568614351319152514994922174201082324235581041612

1257162411825931932328818019581361783154588321560974563882616157634174228510131082581371581175138145543911242061441281607528306211175247542129211101724

586458735250531928950194876655724341703440410162180265318366367304209114501541261551111865922191141355232216192872781381187051631249051128576191412544

0000000000000000000000000000000000000000000000000000000000000000
25688655432104128786650657591289511107723262744221231200689710299962433198808372730299054042705112036414212512949803611736223878117764073304013696235493104341214979521420052914699221946141534182838720613005372104460720174592

0000000000000000000000000000000000000000000000000000000000000000
25688655432104128786650657591289511107723262744221231200689710299962433198808372730299054042705112036414212512949803611736223878117764073304013696235493104341214979521420052914699221946141534182838720613005372104460720174592

25688655432104128786650657591289511107723262744221231200689710299962433198808372730299054042705112036414212512949803611736223878117764073304013696235493104341214979521420052914699221946141534182838720613005372104460720174592

000000000000000000000000000000000000000000000
361437972566437631814950427275313571265627839196432731298216417627574101114

000000000000000000000000000000000000000000000
361437972566437631814950427275313571265627839196432731298216417627574101114

55735041221143296995212212014142

368879725664376310849272741115612516278361763421222031952155743310712

0000000000000000000000000000000000000000000
16437516018573133493983933246113140756610227152636203311221441134104233137381229521511

16437516018573133493983933246113140756610227152636203311221441134104233137381229521511
000000000000625332230159720322211304211611121415210

000000000130000000101
831132223109215246944211

111113

8311322139717424394411

0
1

1

1845312112125122516111
01100000000000000000

1821211

3431112512151611

0001001001
3121221321

3122232

000000000000020040000111000010310011000
16437516018573133492193324699108394167418331813110363121316226173141

1643751601857313349219332469510737405241833181293331218514214

411131311

1

1

11

41

632218122631411516492411
00000000000000000000000

632218122631411516492411
01010601211030004212300

0000000000
1121411716

1121411716
0000000000

1121411716

511211121114512111
000010000012000000

0
2

2

111
000

111

1121121131
0000000000

1121121131

11111211
00000000

111211

1

1

306149137116212211211119412272195
000000000000000000000000

000000000000000000000000
306149137116212211211119412272195

306149137116212211211119412272195
000000000000000000000000

000000000000000000000000
306149137116212211211119412272195

306149137116212211211119412272195

19251641361315111722172195214131
00000000000000000000000000

19251641361315111722172195214131
00000000000000000000000000

19251641361315111722172195214131
00000000000000000000000000

00000000000000000000000000
19251641361315111722172195214131

19251641361315111722172195214131

000000000000000000000000000000004949035539358472218467582483219520564114841363513428102503312564489471080322493988416938616813659113991070692518953819916172834824399116291191814276
83822538916264636462145372849382976210019358266332644219391986217987256164659291673290473352614837304472371221673143660192462326338558621109832249548236270068415253991628513633715456274738658628761961927013264501950110541182552525145746872712860032716154963048023737212467434341890322924449291669799221694815326764

549158813567215838511102001528349615103452168875227518311793583022163877631978553186869711451621671386230782765200356191719714444856778025557204503652931174452296383332565597477752811791406287161076799282
0000000000000000000000000000000000000000000000000000000000000

000000000000000000000000000002043221120111397265434669831645644839827678166126352252182130194322246744223165662189140639184162
549158813567215838511102001528349615103452168875227518311793583022163877631978553186869711451621671386230782765200356191719714444856778025557204503652931174452296383332565597477752811791406287161076799282

0000000000000000000009034752981033912162198895631278414519814213011338
608270249178264186646416512845212811015471131745781674873178424133188108463171354159612873594238300333439232671064106157331312298313745

39764601282122012521371783171315134510484413761511910231881745174
00000011519402221331111913111603151000073

6485653138910111215143214

6012818121352511252354276511

1772579622

2203120814125435371311112311231

813118221151

0000000000000000000003000000
13863110786100811727234331814171120310421345

2355131662213511383632241

13863110551495112121221331411117411104

7555203797815443494214273813111693291410511011958129118231671195485328
0000013341454011141587137228128391092284810417105719545627

13

11

1

1

1

1

75552037932522344129121124761344121191

121111

4621125

3

1

11

8111323

1

1

21121111

1

0000000000000000072020651310200000811110140131
211270249178511866416512827434611317457868487345352618772041811101629161113168811414631007292819

11891178167113802222312913315238371112754113139885

931792495118664165128274179943566848731521173410112277281511130449232477201713

212112
000000

212112

1947018784349151587661953961533122321180191413141163526061064913545144814225111
000000000031114140354226605930472447014144261111358

0000000000000000000000000000
37845876197532223112141463110241653907116

37845876197532223112141463110241653907116

000000000000000000000000000000
12270187349151422301227661231311323411263321211

12270187349151422301227661231311323411263321211

0000000000000000000
351325525132212381213

351325525132212381213

00000000000000000000000000
4001542072345379192016525122617115111322

00000000000000000000000000
4001542072345379192016525122617115111322

4001542072345379192016525122617115111322

000000000003537423248310181111125133971322112795221341321395
1661110200331341109234821812335139869559158321001302837391048112312849962225281731473249533210

4251124221111
0000000000000

4251124221111

00000
13111

13111

166123593733167186166261312127345142275
004128261371641431303129733501110

111

166185622125

1

1

11

1233111221212126

0000017394113191822111111101131820
11102002482032182551391746846873181713152653721

1065200248203218112560572495174515218

4571210312211

135213241512442182324283493563
52002001112231130224241481363

21311421111

2112

1

2111221

221111211

1

00000000000000000000000000000
8334110921453521165110310245532320631626122

8334110921453521165110310245532320631626122

30623436749684144582058630131625348456222502162229215735511
00000000000000000000000000000000000

00000000000000000000000000000000000
30623436749684144582058630131625348456222502162229215735511

30623436749684144582058630131625348456222502162229215735511

61246632412237002839627635165326203420221275341191115210
00000000100054001000110100600101000

0000
1131

1131

121000000200
129121211627

1

12

1

812111127

3253122121933
02200120020

113

322122191

032016201012011520002
23633262211117211561113

233131211523111

11

0000000000000
24664127003111319194151

24664127003111319194151

1657211
0000000

1657211

0000000000000
3111111114917

3111111114917

611111
0000

611111

0000000000001051500400000001310482300000000
19152013552327216356065352923614743833352031426172731215327551811103151441

02972231223371321430245231954226502121
3773977251253581923632246241954326613141

31

377233111111961

51122212112

3112

19152013552327216352295313567810121412217204151913169591441
0000000000000000000000000000000000

19152013552327216352295313567810121412217204151913169591441

1362102594898519112528118930573521076117359572416958442391344549358282552613740529231141481214051769
00000000000000011100000000000000000012000000000

197154499957301035818211612121715661315
000000000000000000000000000

197154499957301035818211612121715661315

3056456107127142221225103740513151811951616
00561071271022102259373941335701051616

304811110019

11

83311161

000000000000000000000000000000000000000
1165871948985191125281140573524131173591544847381311124545240721491361582634473

1165871948985191125281140573524131173591544847381311124545240721491361582634473

8301723969244052818543648418248331615551619321951182175011
000000000000000000000000000000000

000000000000000000000000000000000
8301723969244052818543648418248331615551619321951182175011

8301723969244052818543648418248331615551619321951182175011

00000000000000000000000000000000000000000000000
7148254493289196278593185123089389214492521397743435137826273240255212235141715141681030545263

7148254493289196278593185123089389214492521397743435137826273240255212235141715141681030545263
0000000000000001074833152740152749132010310006472811190380

36965934232827815546214767033246141158118313111114242412

345166107919659163512308934349692111410118541512192428194141931837731181

0000000000000001010000000011010000001021000000
29813217012048914841610081791532391869119516174733852391672046444593278483232403154241759488601301187198113931

00000000000000000000000000000000000
9113217012053234179382391791384343228337381386111324324832118449

9113217012053234179382391791384343228337381386111324324832118449

00000000102242711372612219413744339727015234304423163894014449375349827
20748995416774115169195123303015443616720433745542581742263930542417461453446976113713031

1113111

21142111221

207143232211212417121152

4899541677411516919552151421188926849116331723541272

000000000000000000000000000000000000
24463214872231219351851333417655185111637122173510613349583029365

24463214872231219351851333417655185111637122173510613349583029365
00000104904781122261432414001437112163210313331992355

11131111112

2332331

12111

2446321487223151413131718462127

2895335943131031142031061026404688810514939174511127777421843363730135058731642818145371365321537604
00000000000000000781011400102140110070118201001111

000000000000000000000000000000000000000000
229533594313103114203106102590468561051493917449505220122233201239513153313440121232181130

229533594313103114203106102590468561051493917449505220122233201239513153313440121232181130

17735411131135241
17735411121135241

1

345032162721121221126218234
000000000000000000000

345032162721121221126218234

001100000010000291
5121121611114211472

512211121

16142116

26211043113236342214101554234
001000821100100050020000

1

1

2331

2610311

614

111

111411134422

11

122

191211113

000000000000000000000000000000000000000000000000000000000
109666907054763957891961522171021471478124059185997345912712624011143941042143739890549254222044113627623610715023217280428786883225019801822059833239225128151162

0000000000000000000000000101276003505173014664400011422110110
109666907054763957891961522171021471478124059185997345912712624011143941042143739890549254222044113627623610715023217280428786883225019801822059833239225128151162

10811665705476395753196152217102147147812405918599234591271262179343941042051717875534246191741113326521610314522616273413736482224819321742019228221211119147155
0000000000000000000000000228294568389152580191912726872516811215931030831702035133101518

69651111111111961115
0000000000000000

69651111111111961115

001485312165649412993617911272331434121632380121172158121653109703483
9846275152932917474100641154018111672351734151702485131292861141654109734383

1112

3

1115

111

2

3

279127122131211

121

1

11

141

2221

2752212143711

9567298884111

2111111

735267023161591611021853111281581110281627
00010101112001300808046111020121

526415794421151

7370181714215233212426146

00000599135121192111415146556991719861213948221404171834
3265674470127123161910213362121415156656103919211161230048521416308935

22

5121121

11

2

1

11212

2

213

5

1

5674393127213126212025129411

3267757542521233231

007312622101232080001010
924723920126281313721222532221

9247145763728111421312

2111133411

1921397054765559382714781615911986553043522425101611258610181037264311649591431241431212
000000000000000000000000000000000000000000000000

1921397054765559382714781615911986553043522425101611258610181037264311649591431241431212

2823403631961521791021201051852252373711879394104722854298784131434348746385569612488139604981828133105016
00000000000000000131542136341211651011221032012614051110105

2

121

1

21

102128275621051852252373719410419116282865361012482838668874481044811

111

1

1

1

1

581115

21

12214129146112

18021288196152179102187933589951283132394611159329

11114113

24111

1

1

155253650231882108983253631556191123742441714837
00000000000000000000000000000000000

155253650231882108983253631556191123742441714837
00000000000000000000000000000000000

155253650231882108983253631556191123742441714837

63181386280618886506695136238983824853211131382072224164429634349552289412678710410779378864121544542381155162880585512772175252572477546654196824515522442945678254626602701125707145122738054326521481768
00000000000000000000000000000000000000000000000000000000000000

00000000000000000000000000000000000000000000000000000000000000
63181386280618886506695136238983824853211131382072224164429634349552289412678710410779378864121544542381155162880585512772175252572477546654196824515522442945678254626602701125707145122738054326521481768

000000000000000000000000000000155843062935426238355412283583831241394461185060591283181317465120491498193290161465102165230355
63181386280618886506695136238983824853211131382072224164429634349552289412678710410779378864121544542381155162880585512772175252572477546654196824515522442945678254626602701125707145122738054326521481768

0000000000000000
165373312134221536328

165373312134221536328

41112212119111
00000000102000

1112

41112125111

345828214481497412263513641781281319162373582585320530818225687225964513901512203661181081155292031512085211129137369355
00000001719276314159370582584820429817925583191904313761492203661171021063790429711177161129136369355

345814965653332221347251

2

282988912263513641281296131579415155837

144835891111266138

172

11

32

11

1111

8975118784719232212122392220833192
00003000002000000015120020

897511878171923212122392205713172

00000000000000000000
16318514941312311211111323

16318514941312311211111323

000000000000000000
992633112116212974241151

992633112116212974241151

0000000000
952575171111244

952575171111244

036107501100004151
4313636774123110626181

612622112

370212194223

0662058321021237010000321201314020
1264772563444149995813211237262111366121

9023423561411122242111

7062112111018941

46871119522131711

111

53724946411342916117343907567924826237348554121886326068878156258957322531673114725
000000000000000000100001370110000120301100000

368164331593231611032919527792222622512636385452212356471137166282219312313116

169851335410670147122926122221913543411113222142541327103451169

0000000000000000000000000000000000000000000000000000000000
5175913112366639836238943624827413849272164268461349552287469622541052967183918361387262183621841733147122712338382041729123577114615196672427

5175913112366639836238943624827413849272164268461349552287469622541052967183918361387262183621841733147122712338382041729123577114615196672427

000000000000
308158263512321318152

308158263512321318152

1411518212312
0000000000000

1411518212312

88251114122411813151
00000000000000000

88251114122411813151

8
0

8

11010374961
41115385161

1

21512

16431386221515771654025324131554337867960739255426163515145286303641119244726184316
000000000000000000000000000000000000

16431386221515771654025324131554337867960739255426163515145286303641119244726184316

0000000000000000000000000000000000000000000000000000
39153033488141291251911317725187247890611305934575601444362937201823471504148127281650179491879092561196837356224167355397447923542

39153033488141291251911317725187247890611305934575601444362937201823471504148127281650179491879092561196837356224167355397447923542
0000000000000000000036058355452230000620280000110001754118336610300

8578491868421812428885111157318335357082255175824287132747136416646242
0000191115322011424314026337143961152310201

8578491867781352176473914329783215648194251121432328753155311
00007701302146473814319742712557141041120631024650142241

11

85784918685310453914321813413137

443510211011820000131114036221
4535102110119217911344318198531

1141152111

13932291412

000000000000000000000000000000000000000000000000
3915303348814129125192817725187249012130593457374144436173512541551894601613913761214420136113524105083929727577430

000000000000000000000000000000000000000000000000
3915303348814129125192817725187249012130593457374144436173512541551894601613913761214420136113524105083929727577430

3915303348814129125192817725187249012130593457374144436173512541551894601613913761214420136113524105083929727577430

412193791138021174948481261767700468451527822095193681466670661080297873252277396190701817212162262311868282798285861309212737855387849011005426427966214172783915210717054422337757303430148959276778956514339730653152356052174126219454693013250899755795112621026556241001162715853173218544634
0000000000000000000000000000000000000000000000000000000000000000

000000000000000000000000000000003858241333641433490406393274974914301193589632739729248139131229117355115528478137193463963913932429510261417897
412193791138021174948481261767700468451527822095193681466670661080297873252277396190701817212162262311868282798285861309212737855387849011005426427966214172783915210717054422337757303430148959276778956514339730653152356052174126219454693013250899755795112621026556241001162715853173218544634

335553156906172959724614421771116261213021641061385116556
0000000000000000000000000000000000000

335553156906172959724614421771116261213021641061385116556

21053036205251766848811432241829838924024616783444143268476177579232618556543017133832614211563371921333782705781953020422433119612128261272507671421027328
000000000000000000000000000000000000000000000000000000000000

0000000000000000000000000000951015737800232155270132331901001212912026107101
21053036205251766848811432241829838924024616783444143268476177579232618556543017133832614211563371921333782705781953020422433119612128261272507671421027328

9

11

1784303620525176684881143224182983892402461678144414324499775792326185565430171338189395021231921011126405768722719401432256911685625827739128296823

321206826242274695131281429235224154

11

152742731311023476820561970514550425440717156421013399118246122981929196714551806250812166291484382139150541584772912352303151162741362393868912017957129195426741913950100166152548105
00000000000000000000004402942609694103335615917814714053467617727564107146491151735181270941751787122776

11791257503563192334653132112243291
0000000030000000010821020

125750355316232112211322241

1179114652113

1804311111110111
0000000000000

1804311111110111

1129537817341476818705042393634705649221787156192919671455180625089440381442724234351633172133215194135304927
0000000000000000000000000000000000000000000

1129537817341476818705042393634705649221787156192919671455180625089440381442724234351633172133215194135304927

3112321132522438568
0002211002422408420

3111113131

48

0
1

1

3066148835723366272418116111722112722631217182221
00011045252370050116126031120017171971

148835712181114421125

3066123231111214121

0610000001010000403101201
606112442415212111726311313

60123424311211132321112

12

11231111271
11001000000

2111

1

7

11

111

1207551182610415124132106141612294253383353
0003110501000000010010011011000

3

12075511823911111132961414128421162211

212121131142

2111212
0000000

2111212

000010000000000000000000
926100637120139562511214171228

9261242031132121371228

1005121108435111

85997641441192229512117
02521103103110501200

11

1

859117

72332116112431

1

0000914821514154412223106143441121030920742412696
91357615041612312913918173947182181013714846711223332024973715709

1

1

111

11

1

1

11111

1

5

1

1

11111

1

11

1

21

91316627121111

11

1

1

125

1

11

1111

1

1

576150416124421221110311221215112213

41

1

21

1

235323682434439037011108840344276115116141018216751352391886162
000001417130371204231316136421147321732111

199933682226177355524141839192413254111

18327331113684234141

33562084390192889206348221

144719885673264275122513939441315573793639420143182230180221503283239119104511579238611164589295217244186434033815183212093315191996101141182523219431
00000000000000000000000000067173112011510001020001497030160110

615102319213695926887732936301774084741041603459242038156929361851225423186128683541279
000000000000000000000000000000000000000000000

615102319213695926887732936301774084741041603459242038156929361851225423186128683541279

00000000000000000000000000000000000000000000000000000000
83296537519573751225139335145685036420143182230178451503243155455115797220401868283214152512134153215815189031317848873533111418431

83296537519573751225139335145685036420143182230178451503243155455115797220401868283214152512134153215815189031317848873533111418431

13211483816907996864662584856133836070569961850259883363687255542604374788175671488693431650114527543081655693418121855623617201823921553381083430109345416457122805514730193368071695988457731225962435120456467541116180415961111847891401668640857746987678627171602193903477
0000000000000000000000000000000060842601901546303223150394046243692793524947714067220944173232162353256681776102478090766707290486666596332265405995541176118942664

6561319591125150201852843113615137294275412147542745821243538296511133644486614179646522681386239341053519105401431723119259718226289246112317132138903023329810
000000000000000000000000000000000000000000000000000000000

6561319591125150201852843113615137294275412147542745821243538296511133644486614179646522681386239341053519105401431723119259718226289246112317132138903023329810

792931435259584215428157227311713152283231
000015210000110304370010101100

25911131235323131

111

792931435311150281561111118111

1

11

21

11

1

352642317116243261581540
362842317116254268581542

1

122

1151

222146287333145121841969297153533554911546669311263147151953159
00001451197716430502749325481154666921015410681851859

1307326179111732

92146287333212122633112115113

000000000000000000000
45511621540456251413752438171464310

45511621540456251413752438171464310

93306196471121578432222112
000000000000000000

93306196471121578432222112

13426112
13406012

2

1

14922411561351
00000000000

14922411561351

11015112
00000

11015112

000000000000000000000000000
62120529648164106271676141722271813224136314

62120529648164106271676141722271813224136314

7986553228839313621211128
0000000000000000010

58765531156343211

1111

2111132515111118

451815162212
0000000000

451815162212

0041133536105123331981263133103180322213252
165251511336481051233320211469133103188322213252

1652511112113258

1

68026167735214810225414212
00000000000000000

68026167735214810225414212

0000000000000
9269181111021115421

9269181111021115421

0000200000
1111521121

1121121

113

0000000000000000
802111131123421342

802111131123421342

397211233632782934541853411811816711317499653688971261133
00000011210110082009568922100013180012001

1213123

20212443

23

6242

121

7629123131172110

233113175121

3971611111

111

211631121112510672311342326171112

11

00000000000000
3565136711161721211125

3565136711161721211125

27317716232663311415142
00000000000000000

27317716232663311415142

1622231293
0000000000

1622231293

451211211
0000000

451211211

00000000000
14671874212151

14671874212151

000111000911210001200005100030113
4766587342172736412113911021311193251213

1623212111211

6621111110268211

4758171121972

1111511

1211

1

11

000000000000000000000000000172623191152621915198422268123195625437622362875671143462263135012252663357321686221
9886092316910196010874701179623075924687229405751095136395011043078234257521135296533312787231771260107478155476434928262544131783297566811973717138286025566537222112051171104579743775332

49118610864039493894421936230648174136227722022686235549416641675268437878817137721080351159148477646901514152081121656122294409668342211471371179757

3081096191854972228134540111728793747152811246172986233911574234771162127174134036115611716222177141141320121064551444140582629822215211114151129

189314611322514475214632010331379132697831279127614945231406321035117234415591036173623483211333914154324915221402837478125

000000000000000000000000000000
2792485553911321226610113955222191251218

2792485553911321226610113955222191251218

1111113211
1001000010

321

1111

624137281131102483115334211
62413627113191473113334201

1

111

1

11

1

000000000000000000000000000080000000003100200910000000
778622321912891001692577113710631343455181941372927372171925653798732293132268519372814103295168306696311915714933929214118467323332201012625169

23139117211216

7786223219128910016925779711063345518194137292737217192565379873229313226851937281210029505123398531186145330288139164571220302010125169

13415111

16692151122

231111

2296127774560387917241201241023062140129321572595943256244314141161381612382749154128913926847943158138487
00000000000000000000000000000000000000000000

2296127774560387917241201241023062140129321572595943256244314141161381612382749154128913926847943158138487

4664411623976131244212122869560719211017733262218712
044116139761312142116266955871921907723262218512

2

4661116221112

0000000000000000000070137432883314649432731435416330220533402613029595176992151145233014
4891160511414191427529752511261050490280448344247859613628560225895201284896336776049484663721653928267984401603770703105962623136337626

11111311313238411

4891160511414191427529752511261050490280448344205059613628560225823650158425933121219995814140142181114329311127114012

1111

11

2126125413

42811273926121129312

00000000000000000
4266637111131987312232311

4266637111131987312232311

8762351432422512121711117111534
00000000000000000000000

8762351432422512121711117111534

000000000000000000000000000000000
52210771106723297223276572755655865282214203631315227232454351

52210771106723297223276572755655865282214203631315227232454351

3217313184121
0000000000000

3217313184121

038124672445521711
71239125092651531721

11

71242261

3484452222474172258671767110062038717306436183049431819110112314980444148262736701111731567861618121192
0000000000000000000000000000000000000000000000

3484452222474172258671767110062038717306436183049431819110112314980444148262736701111731567861618121192

000001422120001431101751455148041620738152426248
101535643223003594744261459144882006155143021755213767359

533824111431854423312

1412111213121

16313

52629830018188323311416161

111

261241

111112221

10119324121

31

000004761034016514313879526113001601252110271
913765840936592651176421016111516151815642536344131377135183915

3449316152221312154227111321105

11

13

9132584012241211232115276261211616417241

23111231

21

411

14

2111132115217

1111

142171361

1

1

0000000000000
12117116110111

121731711

1111

1122

000000000000000000
69141315132152133213

69141315132152133213

131350318926286308979397270286335140343812672709120993913856834102253312886103688165928510833499412271281342981170
0000000000000005310773210846924542426122648147612877539325312251741071222967055

471111153210112

111

1311331111

11

521711

11

111

516741267211233211132361

1

11

1644281413138211

1483784253525117541817231

1

1

161221

3

11

35031877828630889139727028633511254381183270951652058522431211552811201441076431110813

00000
7315101

7315101

0620000190210103000110116102001100
102582132221916421046395461791046185183

11

122191

1266

212121

10251122614111636435489141223

111

14

1261111141112666111
00000000000000000

11151413111

15611196651

22410213132861
00000000000

22410213132861

265218361323532222326592144143223
000000000000000000000

265218361323532222326592144143223

21
00

21

893887102119165111172873200741327702724915422161022191106401332551149930212113410
000000000414351116210012912070521843110111121701001309

1137515204511511161327345106111

1

112331611

181211

10211985741212171411121113313

89388740760282889322326189443614

11314231

107211252431

11

36221112
00000000

11

3621112

010023402553716731930101110294
89211241366638172323272101111324

1

111

61

891112120313

1

122

11

11

1211

45781108145121793151036518527104162338434348484372168516141468107256972787355618244314653684528226315964581064551263463211042883316701781212670828316801410928913
0000000000000000000000311109283538238187109631679442016206018911671611491066817496022116313548823

43261026145121797902968205829642875343484808721685130511398246596448028918515212065288335535181332121081731421123148326631315

13016518417373861463519527415476223072491821884913302945151511111215263131471712824192

122821506248026611983631145593238641629144126181235621265111225102937287912

15416517618839623119229767238630248225117124122617409618147411745942221121
00000000000000000000000000000000000000000

15416517618839623119229767238630248225117124122617409618147411745942221121

0000200241
5112212252

5112111

11

000000000000000000000000000000000000000000000000000000000000
68147829301790473227173374716291651810122461284622241126991772666933091962227410611142594815661105139816598723673428133711275231864177162101041274112195931623559621618079

68147829301790473227173374716291651810122461284622241126991772666933091962227410611142594815661105139816598723673428133711275231864177162101041274112195931623559621618079

84841499655593236263134220548154191582017599755763533614133248296181582152454
00000000000000000020000000002000020000000010

84965559323626313422054815410331357965474251350413324629216138112444

841499155174112129422132

00000000
12811421

12811421

1962141153
0000000

1962141153

12553822572032177397516912523131430785524139330645625214466920873366326034522198106451302004945261338100473326774911027490827917915746711519562652196417262495974111
0000000000000000000000834366358016737348532867836237379362699439222515598293841179187127451211138536663

1

115166141

4115

11111111213613898

122511

31

122151

1

11

11

3822521112221221

20373987306283190330513251152027036976524751133663812567429

26312212121311161

11

66111432133211512

11

711

7515731428910137140269513965682091541334314555103158436311925208254

43

1

1311

1

11121211131

11

111111722

11

765231711861311301527

11213

2222171711

224121

12

5521172624111

2

1121171

110725718264464701299191317212116165642291

2

1

1

12122

1

1

192111

21719115982103931734801733457761414111161711011941252559113820

32

311

11

12

1

11

561321151213132111

122321121312353

86102131111

1111118

5

12

491401123111221131162112215

2

2

1

2331431

1

11

62135101121223834112111283

11212

112

623307810967821571115102122042208522

1

5434152233

21

1133312

1

11

2

4110111511812214212118202111624
09101407121141501720001600

1

4111181511711121

1

131113

1

000000000000000210010000101000008030110001
105211240888579986613210077299222439127281792011462355359311193295135661

11

1

2

1052112408885799866132100772992024391062817820114613443593111841652566

11181211
00000000

11181211

000000000000000000000000000000000000000000000000
107290885110914050332139813351529146065271512934365476196036472541812352146892111124544319228193698170129332222338

107290885110914050332139813351529146065271512934365476196036472541812352146892111124544319228193698170129332222338

0000000000000000000000000000000000000
207231145163234173471611705528111453153123122181917512824336112032

207231145163234173471611705528111453153123122181917512824336112032

0000000000000000000000000000
40310985336487155266621339854311327044151

40310985336487155266621339854311327044151

0000000000000000000000000000
3386082734034103417813144195312123121142316223

3386082734034103417813144195312123121142316223

11511391511
00000000000

11511391511

35598520504861538121315218122211472191
00000000000000000000000000

35598520504861538121315218122211472191

3111131117372
001021010351

3

211122

119121

0000000000000000000000000000
26871146221212155422115643171331

26871146221212155422115643171331
0000000000000000000000000000

26871146221212155422115643171331

201104112
0000000

201104112

1779270772311184463123086514349622979555313417955641770321434177868139273998310178279870333756413828432055910576721678117238
00000000000000000000000000000000000000000000000000000

00000000000000000000000000000000000000000000000000000
1779270772311184463123086514349622979555313417955641770321434177868139273998310178279870333756413828432055910576721678117238

1779270772311184463123086514349622979555313417955641770321434177868139273998310178279870333756413828432055910576721678117238

7068498978431501119718530652205514637301054253415105884598807861144413746596205911940768774720028451794481760823448432752596089591427176218144643061945228346863367624139196017123557199020423422157
0000000000000000000000000000005921221294355311211836744433514030532243219441031812387194161326555515825

62598642445229832381134025100915457314421166141071551216425231611891
0000000000000000000000000000000000000000

62598642445229832381134025100915457314421166141071551216425231611891

644349897745150111971853065220550823730105425101510588455357877814441374659620591170276876342002805176948176082344151144115132928214637130962924414816216137161231136206589889413481381715431
00000000000000000000000000000000000000000000000000000000000000

644349897745150111971853065220550823730105425101510588455357877814441374659620591170276876342002805176948176082344151144115132928214637130962924414816216137161231136206589889413481381715431

0000000000000000000000000000000000000000000000000000000000000000
3832150516611479332264133120425410227171038433013129343411289220725811259817010870118193139422148831367235131731824120325722717057468047243829519402547112147201349934125119

3832150516611479332264133120425410227171038433013129343411289220725811259817010870118193139422148831367235131731824120325722717057468047243829519402547112147201349934125119
0000000000000000000000000000000000000000000000000000000000000000

3832150516611479332264133120425410227171038433013129343411289220725811259817010870118193139422148831367235131731824120325722717057468047243829519402547112147201349934125119

120813485110058838416911153561163729693015378632141184506514939134681026389621379714623444383386173997846336709283724523695833019371166476479325639291991136551741342262046231830142253123535191011810783845451705292108027122034138113311172249021249193919024547116456621
000000000000000000000000000000001550610653388189273369033342532225187162109880577345563630886035922656421284488616672590692666

6841237742981599122025021646182551246434782456547300612908123717388620711641104229602952963035191281582814751380117348031343213936289017892645114367926026427011525157724541704634223880689279615539964106421160914016238194011003785101111501061349395685667
0000000000000000000000000000000031521429015867301429380194168599214867870967133127245145128464257872144310051382534406

0000000000000000000000000000024211159811244382974391313261117121683101129904153
415725398322064089177877784877337476796292116081123106919336986823230987923492104074055862908324375939583915259815670513120230218814880101662745349432344145624582460331

7892532972201134764951024428376551104417124216431098139401321471061272428111815816054304251723726184122661176521263613216611
00000000000000000000000000000000000000000000000000000000

7892532972201134764951024428376551104417124216431098139401321471061272428111815816054304251723726184122661176521263613216611

83131422722671461503742996063842645211130456518111734392251711572473173712276141164520121536597926259465326221190201418216316883139
00000000000000000000000000000000000000000000000000000000

83131422722671461503742996063842645211130456518111734392251711572473173712276141164520121536597926259465326221190201418216316883139

000000000000000000000000000000000000000000000000
328568639685745834044223151114815548632180364445129661065161913717542310102773129615817103216710445361260728

328568639685745834044223151114815548632180364445129661065161913717542310102773129615817103216710445361260728

37823224840617098550215129642068103757711051233488818356123216245978016248106119546224191965068431638469959166815975771656694021885288126058121592412136172939
000000000000000000000895675363094671839229252483155724682075392111656194378518382321841028803

3783321709816942647198610374343105107148872635612323714775514332269265151092418156685575103810983601015392651163043417239652532322702108
0000000000000000010723187195732481267835662172525372626154801942310126922131614841339

209332170981545516123201422251289212412338164702061342235149113341041211531527125

76313045773097072542171034054522755143211901891052741223229319361182243

169777134313547282024105497346284253699234147591261142410622325471135115424406517621433501

00000000000007000011011012002056216000100212
232248745504573178214281629216288326116181896102081161713669583110102921163010328111431928

23224874241237828191629216243916371816951589413334120651213815912641213415

55021680609444982219411151313617944616791217216411

230618923067137958016121624341604161626072082471166078367799419741013937239172717825582253283342471475117011333901324930122003143221778924921672161953131412592173157847314196129671437393008207724110351040489715597302946933935962293229571991
0000000000000000000000000000000011089161441597261981341006872878151593921452038434359154117321215150646634169994425916602932951231432830837

0000000000000000000000000000000000000000000000000000000000
54025962833912322914815027216084734474662102192611293151752607598284951179323622123255516163625183180926933264811651573511265403413

54025962833912322914815027216084734474662102192611293151752607598284951179323622123255516163625183180926933264811651573511265403413

194234277109774710031143197128221521192009755734144771924159665664235022214321792655717014161689917371911224469287
0000000000000000000000000000000000000000000000000000000

194234277109774710031143197128221521192009755734144771924159665664235022214321792655717014161689917371911224469287

63188418175691312121680914569412036141286425294340029601579808554406591504105836521334063672521941227955239198432120621352368340214526451473061113134653758227317122183553280195681128205133571106214
0000000000000000000000000000000000000000000000000000000000000000

63188418175691312121680914569412036141286425294340029601579808554406591504105836521334063672521941227955239198432120621352368340214526451473061113134653758227317122183553280195681128205133571106214

9415153454713261407762892522152707532974218514201360183518497161090459518879325823861975241520393438431212145121423914623810712413773571323153644514655
00000000000000000000000000000000000000000000000000000000000

9415153454713261407762892522152707532974218514201360183518497161090459518879325823861975241520393438431212145121423914623810712413773571323153644514655

51115919526612751018138689191431638426596072259889534038041246226719404292051711281031819390811177011712625678971411845404235617616924102712014984843436528061894915
00000000000000000000041920316512610118153227298599710622463960510034593615415014223022171763973893005616941592772

435195197975848110279891431638418976072259889534037381246221718413243427491881289771156048192151371989893276100283125

7615969202572009332421117115219114312471692332493517965

9811384438665099521291139182019554333837541531313

524011086707723929498651407019418551308758637205920311097287527552156420484881765436459335288113621072252235275939271120398576314604972301168351883162382985141119952699152671955035124327453087294946691526237694641385288
000000000000000000000000000000006675092451497816618244886048116811691682146596756938993170492361660328623461

2955464267926290084024230751222113262403192191141712827691088215045180474136100978211548921682236753165216218313178172453710848101234858389219601363237232017155112099664340554322349242
0000000000000000000000000000010100000000000000000000000000001

15904642679268768402423151222113262403151191141771576910882095237194136100724211548960554647988213128017513137155829922168273511654433482252779007075454038153920139219
0000000000000000000000000000000000000000000000000000000000000

15904642679268768402423151222113262403151191141771576910882095237194136100724211548960554647988213128017513137155829922168273511654433482252779007075454038153920139219

0000000000073591297880363204414617132638999481151312151638221207
1365202420764105671940949432802541563218210567133873334108225019918313854158151221131065150242132442210022

1112141173

1132131

81112211

513231214433111

1111

136520242076410567194094943280156321821122621140213115494746914501271737312

11

111

1111231

4111

25475112112115

3334614521164100401617881141182416111516535114510282327196
0000001132100301000001000002200000210

81052229142231423111233323111
8952224142231422101223221111

11

141112

000000000000000000000000000000000
3334614521164100315945119321310111322520525212275

3334614521164100315945119321310111322520525212275

1085382467525125375116343110207241317470131123751742310759105485
00000414164302053052892335143412514761202413

122323116114141112331941
12232310674141111120941

41211

1

000000000000
421425214461

421425214461

1085382467525152115161694644110683332411321
000000001420026164045131000000

10847420375161521111557383291321914111

644390131111051111122

3113122116213
1000001000001

0
1

1

000000000000
211312111522

211312111422

1

1

4191627460337472811259783497230361521442111123878221411118482
0000000000100010000110000511531420

41916274603374728112597729627242133823776178513432
00000000000000000000000000

41916274603374728112597729627242133823776178513432

1322315111011315141313
010220100110203040302

1211141911111111

111

83624385387269113903445292245631314201378717069871422453217468152482631039234313835
000000614301310183275631510185332291281014134013176

5132121115411017161433621311231234132
00021102319677312111682123271

1211111

1121

51311219842410214

141121

8362387238726911382191315213350108138715838435561636281434611235110735202610827
0000004810751211261344222327223631223271017656268141511207219

1

321

2305211115218211

1532578245167154112248192321

642691137161213414621111374614982214

1

11

12

13

49111142242121

8226119111

1111311211

11

2

71

1123111311

1111822216532

121231

1121191

83623713014312262755337120201

2

12211161

143312538115362830433543914846221062323841138873811361442526962331917262076111981496874271414741132
000000000000000000000000000000000000000000000000000

000000000000000000000000000000000000000000000000000
143312538115362830433543914846221062323841138873811361442526962331917262076111981496874271414741132

143312538115362830433543914846221062323841138873811361442526962331917262076111981496874271414741132

0000000000000000000010101001001000000000
130698107605126987717259151281213330141041914164362841337192145631532474

00000000000003000020025010036400000000
13069810727269769153571010712333013104962220254104156143631231362

5411111

211121

11511113513

31147112

4126

162981072726933413223320122310233874411191

11444351533458496123311211316721221

6024108192521119813215323335231112
0000000000000000000000000

6024108192521119813215323335231112

00000000000000000000000000000000000000000000000000000000000
25986724934725232919538839301741956756577142593101633500891559440722741738793171481322129486642892018667331614587272812171051711627481415930283

00000000000000000000000000000000000000000000000000000000000
25986724934725232919538839301741956756577142593101633500891559440722741738793171481322129486642892018667331614587272812171051711627481415930283

25986724934725232919538839301741956756577142593101633500891559440722741738793171481322129486642892018667331614587272812171051711627481415930283

00000000000000000
332419621412161191133

332419621412161191133

21155840181121675210172913227159334611072076936151843179929112417564173469335414439582940143132011
0000000000000051138742100902221141200014500111102

115119111
005116111

11

3

15931612461
0000000

1593162461

1

00
11

11

21155840112167521017291322734611071445219612221065151019103813474262622107311227341228167
0000000000003311125360030202022000015203910202

11114111

911111114

211

21

2115584085311526113129211

642

4131

11

293527124222030156216

53

3461

1203461121141352211241413132

213211

433

1181112

11247525744121251273622112361131

4

402121

1017110741122011121

33111111111715

020711101400139913001105101200
18729111341441331617211122110215431

32111

1212

18121

2144115871111412231

000000000
321121114

321121114

29193051221513225131622164221
00000000000000000000000

00000000000000000000000
29193051221513225131622164221

29193051221513225131622164221

000000000000000000020000000110300
6297631345911201471317455165211419152752893621114236

00020002121293311221510112
6297631345911201217323517129315423821214

23121

62976313820121

459121862225210

1212321

1112

11211

000000000000000000000000000
261310423134210101215115181196

1241213428111511517136

2513811292116

11

740781385311367013765021560503223282059765494148487571415511337993153589173688247777139636112845283859326946380648287
000000000000000000000121000000044720000000014002100000

5413852903670137629156605011267857465492148591413328799290348012962115566653417411111264956213876230296523
00000000000000000000000000000000000000000000000000

5413852903670137629156605011267857465492148591413328799290348012962115566653417411111264956213876230296523
0000000000000000003310320500174434318352818501000102522751820933001

0000000000000010100111532302110160280513030
541313707521323509602566521482411133317146451101234221071159415334871219182

000000000000000000000
2361351151261213212322712

2361351151261213212322712

5413137075213508992566521482151322112614663842083151312341421815
00000000000000000000000000000000000

5413137075213508992566521482151322112614663842083151312341421815

852902362416133601117183184922571828319934177114271565311110301825213597172
00000000000000000000000000000000000000

00000000000020000041211264533100531111012104380
852902362416133601117183184922571828319934177114271565311110301825213597172

8529023402801336011120122515718283195123310621522111297435493372

22136517934911032225320228516212

32125910711633041225415114
0000000000000000000

0000000000000000000
32125910711633041225415114

32125910711633041225415114
00010710000000501114

321259133041224914
000000000000

321259133041224914

0
6

6

0000000000000000000000000000000000000
686782121598323412768711840852594063799621626514121308449351364

686782121598323412768711840852594063799621626514121308449351364
0000000000000000000000000000000000000

686782121598323412768711840852594063799621626514121308449351364
00000000033456213004242335300200000701044500

26178597420611724131222
0000000000000000

26178597420611724131222

3994125339632451512192171122962336341
0018532632341312162171122542320111

1

20724113

19241404321623

41111

1

26212183232726372221211452615314822351726254
00000000000000000000000000000000

26212183232726372221211452615314822351726254

4141545444183071154157331052321201423362
00283251425201405510742111151220250

029110000000020000
2141331745322311142111

00000000000000
379333122122111

379333122122111
00000000000000

379333122122111
349230000012100

1

3121221111

00000000
21467721111

00000000
21467721111

21467721111
00000000

21467721111

000000000000000
20015362111412191122

20015362111412191122

21
00

21

63817375936665603667115166894673836256739388329473661852511063586471336210769102454744915993502614881475911017175744009218445631031519498449552257321843856994130109516586866432697956423829374075840610497517581379410442973746174170465406145165150124121578339482404438911738195444622
0000000000000000000000000000000038721600000002600001000130202102701

19193121221161152123
0000000000000000

19193121221161152123
0000000000000000

19193121221161152123
0000000000000000

19193121221161152123
0000000000000000

19193121221161152123

00000000000000000000000000000000631802761199923212332839535152386153165563213198170578449172439152153379754353291555176
63817375936665603667115166894673836256739388329473642852511063586471336210769102454744915993502614862475911017175744009218445631031519498449552197234841656934130109516586866432697954423829354057840410496517571378810441973746174170455405144985148124101578239462401438911708195444621

0024012402000100010937221100032
743138108321511221336421218156786651113126

231111121
000010021

111
000

111

1311
0101

21

1

00936507110001020321136214090032
74111410711449121354112912586454111393

112211
100010

221

1

01001100040010000007100011000
7412132173821252197120221411361

7421211734211521971131214361

63817375936665603667115166894673836256739388329473642852511063586471336210769102454744915993502614788475911017175744009218445631031519498449551537041813255644023107196460831532297919407528953994835010328511911356910239956545584165785218139924988122511539738662347433511414189774439
000000000000000000000000000000008261605252610109724099949317642953011113370750836148663381573160513445139481815323557159620922874706441569304173631497

000000000000000000000000000284771066183831484710365172719692412661601476171544888
69325525352422643645175299384567388433865517938333824211664845472290496378159010653442539750268419204792192432231429849158864698114199332429665247467953658198365435791464466350136128893923082939498

00000000000000000000000000055717503721651691831023638111683907323940260624554096676873147187560427491068390307128116282320742347451
69325525352422643645175299384567384033865517938333824211664845472290496378159010653442539750265618734702092352171229045142824538014151320328925037197733586195964134981276451340129127192422622846487

4242135610432131113221

5831217711125

61523967302648483692481129512972431110253172394

11111221

90391101512339252112432114317913686531131210478

106161169342225104240171501753787914520521120184975955066181041651882236193

14543194242955931168302871821457166906005021512202110115287225151713171033618151268911410

364175452633353512165048584161827124214321141522311824214317591061

2111

844023017524461

11122131151

15288144259252925187633042794962219730417299241611792032214623445922162953412

292312342993082044282402093571338131216580364383354915115720201085614627858410840592341332749358

2353331183291781113

1211111131231161

66926105837632132149393175615321231984

60180187254030621506410628841183161018645291312254131714

4422118122118943311528131253
0210140001653111927031202

441122122131

21221123151

00000000000000000000000000000000000000000
9852312625244130416691633449988355332671183290919297180228417432149186229838424039717827813171334520166852110931209153989311903072866

9852312625244130416691633449988355332671183290919297180228417432149186229838424039717827813171334520166852110931209153989311903072866
000000000348329668183289895297178928314831949183164137022738817827812831328517162351310871204141909011842915806

114116

36623662112

98550113041841233125521831352112539

487219021741

112

1421669175563311173126821372228233113

23111292441633090932227481236166112117946

00000000011666711811288631151286820202123231207546102284730
26792174215866972485931069339135222620249725208192045837326479733577785216173594107118138

00000
21111

21111

26792174215866972485931069223691515121367171450815330312444595112546641311274827971108
00000000002269141310136617450813129161723314112545458059322674852

86122134101

1

211221

111

26792174215697248593106910111542128616516815102255

17338331143299174323351622997225267133018830312024015311365658583313857195296119105429021612683040521113375007623611211741455732182212
000000000000000000000000000000000000000000

00000000003001732911162231500132446829311335517125711096225920312077339520310443987383481131371305612094204
17338331143299174323351622997225267133018830312024015311365658583313857195296119105429021612683040521113375007623611211741455732182212

1

1242

51

1

2

520181222213

11312211

1

31111111

163277291131142111744311265122

1733187462322016311194141822351

11121

1

1405891180416575121411

11431704516241121611216636241294314

111

2335612211712129108113126

11

1

3921722191712082183321114

151

136217106454711228323211271

83313904131271651

5

411

1

111

1

299645925385629534038434689452343511324138832418270473071795314412811980291973996813877437847261379105211625938463693498646759561755283828604005400022483924503623482678121923291940202261949404053710747742969373453776782219363883278301032527213132579412731331160
00000000000000000000000000000000194020992844282414262886299714392109816618651496133231225802674259505214475917190494614431978529570970961878177115330792005874

1182941026507910449289595208174113310218585031926327849352649731395412546111735295212835826157894590111395940172339512643
00000000000000626634220124345551321729384510657153613109784337410312513826410029

1186597721919658718523431910375992371123802555143344911128161

13512957123111107130416115123037317

1591125160531221813122213118410522153

487092602481956162213484517154871138421112394

367364486102342251402558178834661711222492

8053166111410322114

2833415851066295315080776800398032995241388324182469239194441540943893489197393841349539313909125986961582430843697175607297513848136361001107372994619567175374384013825741195491052432582083211316989255666512832291829306555683386843597184
000000000000000000000000000000002238912622417928642834780461118998641061442970298459610771419541188590709781530715926755

88140420111519111424279394731

1003123924

1

184631174721472721231101703154217314067815219153667032297216301313167131234212344398710742752582335118122104311

123111

1228

9999919441592571555343082312194174851911456554938325114903522142491140681202025815872391571052042154291140257304311322881021214855104041478399324874791634322412617

7311111

72511461407148189377355634711003241131182214826171194019002371128658070499139165214201061110161763630427124539133425111722359245714154156367921842148097504233171792266443539325228432111349

7321141

46923432892203614318644133214718174092935512985820846794433918861159323105831964821618137019311122974305467924138222320149629145599275401615336051716633053612336161205922915223202153840

6405708076504591286269219649068539550927567481098118243419122982188371365360613651987950895651844715548231254643591216314954372471211848563150113912

45631113927025372448215726842210257719311065872420143919186711931117289933182126283157273233010261235267922922224
0000000000000000011116287124570412111376071822362421030113712411265177212

62712211247537153

4563111259702471307268364102169801065712124338511114603016615217701584414101

9911111301

13325372201855857710369858733334243468522592433311144994303714011

3541114113115682
00000000000000

3541131111672

1112141

000000000000000000000002332188810520961171449276388143409812618161015216329
4342791902469496070016523884707263287273382447643120270231541191628435696815394827112507112042784313612645813159622593345441210158117533

12113221

22922117445423741011621232351

1111

8264195111111618182442

12

189279190960165842733824472671202315411090284518335258580371172101864744748691082963489520

11516112

24524694471123486263113376270313821311727121911553261213225352142594

26922313446233043820501501581432181114941310421749799614166131633748276201656614633
00000000000010000000201120000120330100112711

11514121322
00000000000

11514121322

26922313446233043820501501581432181113931310411247789412166131113705273191346311722
0000000000032002000305633220010151214061441

269223134462312220501501581099113102211223211313212132699715844

18238143218116597131651929916

1

2

1

1

11

11

1

91

00000000000000000000000000000
154188893903313221511233314254462948122435

000000100304010130221629112002232
154188893903313221511233314254462948122435

54931121241621122

1111

154183490213181123111312815111

0000000000000000
4211115423228715

4211115423228715
0000000000000000

0000000000000000
4211115423228715

4211115423228715
0000000000000000

0000000000000000
4211115423228715

4211115423228715

52101597118116769116112528569348321613381282383
0000000000000000000000000000000

0000000000000000000000000000000
52101597118116769116112528569348321613381282383

0000000000000000000000000000000
52101597118116769116112528569348321613381282383

0000000100000010110000000160010
52101597118116769116112528569348321613381282383

00000000000000000000000000
521015971181167611113285573483116103722373

521015971181167611113285573483116103722373

715111111312
401001011210

3141112

1

000000000000000000000
22147183121216613211733111

000000000000000000000
22147183121216613211733111

000000000000000000000
22147183121216613211733111

22147183121216613211733111
000000000000000000000

22147183121216613211733111
000000000000000000000

22147183121216613211733111

54279126750106279231120181311232612864129764491763080328367141388116288228125538442120923186437961409012359589634919
0000000000000000000236832630026341662000111000311371413030100

283026216352551626517415116174623822163818313
000000000000000000000000000000

283026216352551626517415116174623822163818313
0401016252437001141131001701192851216313

21123
00123

00
21

2

1

193229264322333129
000000000000000

193229263322329116
193229264322333129

11

13

3

28522111491142
000000000000

000000000000
28522111491142

28522111491142

8333112
001219

83213

00000000000
84313117421

00000000000
84313117421

84313117421
00000000000

84313117421

5427372675010627923112013112326128641258642117561717238589322105251206111214351534763337103381058414342155
00000000000000000015247810525003741688812100000002946145851720200

38106174100269264493136722172834351112331011461
00000000000000000000000000

00000000000000000000000000
38106174100269264493136722172834351112331011461

38106174100269264493136722172834351112331011461

380398881052336427359209751671611513610113235327431624132889191641
000000027216158121371612100000001477191320100

45145241011313632202106162539411521
4014524101131362020296152539411411

5121111

1901316012611121121211132
00000000000000000

1901316012611121121211132

105541221526111013231110612
00000000000000000000000

105541221526111013231110612

19039888105362122627121217812121335
0000000000000000000

19039888105362122627121217812121335

124339179501311201311338642117372451152313448864112102117115163134
000000000000000000000000000000000000

124339179501311201311338642117372451152313448864112102117115163134
000000000000000000000000000000000000

124339179501311201311338642117372451152313448864112102117115163134

000000000000000000000
541839812431114131931083141

000000000000000000000
541839812431114131931083141

000000000000000000000
541839812431114131931083141

541839812431114131931083141

1224112221112
00000000000

1224112221112
00000000000

1224112221112
00000000000

00000000000
1224112221112

1214112210012
1224112221112

1

111

21331417173415116261171513231
00000000000000000000000

21331417173415116261171513231
00000000000000000000000

00000000000000000000000
21331417173415116261171513231

00000000000000000000000
21331417173415116261171513231

21331417173415116261171513231
00000000000000000000000

21331417173415116261171513231

00000000000000000000000000000000000000000000000
45182393217216489445424255220717151439274918875662451291134221171910221643336641329671737331215151782

45182393217216489445424255220717151439274918875662451291134221171910221643336641329671737331215151782
00000000000000000000000000000000000000000000000

45182393217216489445424255220717151439274918875662451291134221171910221643336641329671737331215151782
00000000000000000200000910000010000007201000003

173216211151151632512
0000000000000000000

0000000000000000000
173216211151151632512

173216211151151632512

00000000
31112111

00000000
31112111

31112111

0000000000000000000000
1222327162114326211218

1222327162114326211218
0000000000000000000000

1222327162114326211218

0000000000000000000000000000
143239151232221127413673335487281091

0000000000000000000000000000
143239151232221127413673335487281091

143239151232221127413673335487281091

00000000000000100000030000000001111002000003
3118239172164894453682552207171439273013311332239543179242211433360350195116031824141778

3118301721648944536825522071439272913313329154317923221043235934919491531113139775
000000000000000000000000000000000000000000

3118301721648944536825522071439272913313329154317923221043235934919491531113139775

0000000000000
2091711918125217112

2091711918125217112

00000000000000
6211113211147113

6211113211147113
00000000000000

00000000000000
6211113211147113

6211113211147113
00000000000000

6211113211147113
00000000000000

6211113211147113

0000000000000000000000
85175511910112421151116436918

0000000000000000000000
85175511910112421151116436918

85175511910112421151116436918
0000000000000000000000

85175511910112421151116436918
0000000000000000000000

85175511910112421151116436918
85175511910112421121115436918

3

1

3348334721211113492325211071221126
002001000000000000000000000

1311421321121
0000000000000

1311421321121
0000000000000

0000000000000
1311421321121

0000000000000
1311421321121

1311421321121

111121121112255211
0000000000000000

111121121112255211
0000000000000000

0000000000000000
111121121112255211

111121121112255211
0000000000000000

111121121112255211

00000000000000000
224820251713481121815

00000000000000000
224820251713481121815

224820251713481121815
00000000000000000

224820251713481121815
00000000000000000

224820251713481121815

168112139168287873511039271174105867902216422711415429715818792130131233359090117742362101362592061838112745959183621602476015914617017942
00000000000000000003014151222510211224311019118223910392110511515336227628102

472659577632189481141381024329369211463241914102
0000000000100000000000000000000000

000000000000002000000000000000000
474551452116648112969193243659746118111271

07101020340262411200018233050410
47273142143341666515333638334185771

111

1

4

471121

111441

121613

1211

221812112441

11

31

1

1111112111111232

1

14111231322112111843
700000000211001000

411113161522

31112131321

1

1

411211414211931222
000001302010421000

41211512

12

1

1

1221

06011112010113130100020
26146311122242155334268231

66
55

1

1

1412414221
00000000

2

141241321

1

00000000000000
1216221411021681

1421

1222141102681

2021241151361144711731224111
00000000000000000000000000

2021241151361144711731224111
00000002000000000000001000

000000000000000
20114134211212211

000000000000000
20114134211212211

20114134211212211

1213121123715331
0000000000000000

0000000000000000
1213121123715331

1213121123715331

1211121391682878735110392711741058674422164227114154206138163691011221129169617557202185116223136169761094088704233140206491095112715837
00000000000000000001112112801011611411220300011041512210147602

0000300000000010000000000000000000
181671135814335422121416310410142623137423311338113315

167113561423542211941431041014262313732138338103315
000000000000000000000000000000000

16111118211
000000000

16111118211

0047363011003000192403200001122102792013
71135604235422183143104914262313651938337103314

1

120113

2

3432131123218

1141461

11

12522

21121

22

11

1

211111221

111181116111

12

1

71151101111033211

1

1

1

217321

114114

2

1

18171211231

8011211027187110175174105863151751141547388934279482594456494264151971526715775983377249911021394550307514132
000000000000001400000200402000000001171324310221

000
121

21
10

1

1

0
1

1

8011211027187110175174105863151751141547184934277482574456454262151971526715775983376232781001354248307313931
0000000000000000000000000000000000000000000000

8011211027187110175174105863151751141547184934277482574456454262151971526715775983376232781001354248307313931
00000000000000205163356948214384933363714177141591336984286416967871213932264910527

36221123311814271

1

3

1812231632111218211

11

5111

27111017517410586279175731543792736646376191162461241235691134210343

6211211087412921201413111113

111

00000000000000000000000000000000000000000
23291683521729422164524163322810821021341120231765237186727710423351731323

00000000053126318046300125101200041065637030100
23291683521729422164524163322810821021341120231765237186727710423351731323

00000000000000000000000
23297418822131161119243244213104312122

23297418822131161119243244213104312122

217643113218142346811727412511421
0000000000000000000000000000

217643113218142346811727412511421

0000000100010010101
5135103211261612811119121

31

51421111171

22

353331612791

1

0011101
1111211

11

11

000000000000000000000000
4921552412437525634121310122

4921552412437525634121310122

000000000000000000
4357213220111111197416

4357213220111111197416

0000000000000000000
10353123114211033265712

0000000000000000000
10353123114211033265712

0000000000000000000
10353123114211033265712

0000000000000000000
10353123114211033265712

10353123114211033265712
0000000000000000000

10353123114211033265712

000000000000000000000000000000000
11148925036929213734347441214872061641202617115411214225612060122620415

000000000000000000000000000000000
11148925036929213734347441214872061641202617115411214225612060122620415

11148925036929213734347441214872061641202617115411214225612060122620415
0000000000183809041122030110000024111570

0000000000000000000000
44313122813161253516141214

0000000000000000000000
44313122813161253516141214

44313122813161253516141214

11148925036929293343474490301126737712712478126175010214131
000000000011372023410000000600000

1075892503692929325444902698750548612476123524917412
000000000000000000000000000

1075892503692929325444902698750548612476123524917412

39349321232312201411
00000000000000

39349321232312201411

1111
0000

1111
0000

1111

3115119123
0000000000

0000000000
3115119123

3115119123

318134581505101520817071480123919993492511137534647524948562467455447142814041124417954204132473147217410082425181789555513219133286287515182234500132497541451432381503401772051184624621135118326042916361098320725
0000000000000000000000000000000000000000000000000000000000000

318134581505101520817071480123919993492511137534647524948562467455447142814041124417954204132473147217410082425181789555513219133286287515182234500132497541451432381503401772051184624621135118326042916361098320725
0000000000000000000000000000000000000000000000000000000000000

000000000000000000000000000007218823452745807594381496894144025077358382258819761092281451285074148444979916625256071264408
318134581505101520817071480123919993492511137534647524948562467455447142814041124417954204132473147217410082425181789555513219133286287515182234500132497541451432381503401772051184624621135118326042916361098320725

00000000000000000000000000000000
2152181461609714116912614122914584113223486273101310191911236352110286523510616175

02141461609613116912614122313184113223485272101310191907236352110286423510516175
2152181461609714116912614122914584113223486273101310191911236352110286523510616175

21541614131

1111

0000000000000000000000000000000000000000000000000000000000000
31813458150510152081707148012391999327751113753464752494856246745544714281404112441795420413247314721741008148678939018460115693552383956491664410713614409893109225848617423342746611284128095242

0000000000000000000000000000000000000000000000000000000000000
31813458150510152081707148012391999327751113753464752494856246745544714281404112441795420413247314721741008148678939018460115693552383956491664410713614409893109225848617423342746611284128095242

31813458150510152081707148012391999327751113753464752494856246745544714281404112441795420413247314721741008148678939018460115693552383956491664410713614409893109225848617423342746611284128095242

000000000000000000000000000000001730986110343617361516716497518048914086699911066913118391713231742385855929322148316173697414001308782326
187983331568820242609040132197940738865252650384495542140716581251236476150908713587953011821342532309033081133192784663618169620176391902313353662715429537410778960372000171724131665034836132421500230041966255118063181352054022667515404262159924362583781360760510352792092718533570585646524289113992214903

00000000000000000000000000000000642934222124281126653824421281441641162394425302911242810711371561924
102392435345411134554615384537741587176012151953113737584450030368111781260736772761649821322765626112668506222590606162921628134415178401912235037842983391827785864522966374906639371280118722831567182236117429627891157332860495554192727591328

33442929053163411896201216270676424549970108916111451105796203332244208911115178381144166689972585246160601811492624444685717331107147211558171132566421824520027495341143172
000000000000000000000000004938246825171782215191341141311151122421812171235575410151328120

0000000120001301101910154000007505122300
712744005993281533419161128825967815703260231413276862062867198811

7040020021000000110000
9241421251212111521111

1

221312121114111

12211

0
2

2

021122210101230011
153224322111114316211

153262

1

1

311

1

000001180420071225171836566061128314220100
7127440059932825141211242157336861204217131116568106225663710

61152411414316141242

1

13112120111113

111122

274111123

42

7176171351276123011466

12

269599252132121031131

0002252001404103201112339112540111204120034
180132810281018714172524814158133911588198545114346912706453031324627

0016954121210196741397651384179983784464441953318
132810217105515161119674149965140432101737810465842153318

1143511

13281021141129151101

211213

11201262031971332
11211262181971332

1

15

00000
11121

11121

0100000000000012110
1801112121211421112115

18021111415

1112121

110001021
122111141

112

11

2

000
131

131

00000000000000000
31156616315242112

31156616315242112

0051200001000015000720300
18515219820121341511337911111231532

0
1

1

1851163231
0033201

123

18511

11311
00000

11311

11
00

11

000000000
132121122

132121122

1121
0000

1121

002010915224021
15213511212132225121

2

15232711

4

931

0000000000000000000008943649133134159606049931094611701421311330166203902
2631551051126552011667812442454930522328332223296203255919112264140232419339107531357601089915020927511457257765352671468084124262604116

1122431
0000000

1122431

1102111032130111163241
1113211343133211473241

1

1

1121113

1

111

0000000000000013000000001000
243169203131326521024141011308313614236

24312101012

69203165272128821348232

1

1

31

11311144

13

1
0

1

014013299841194033
96252153311546475253

2112

6781212

1

1

29113113

1

11

2433

2311
2111

2

0000000012100220411202100044500402181
87720116612427923225586212211199291142015351121551811139481895

2111642

87720116612427923225586211117751318153221113513561694

25573122114126766112
20573022000103525000

31114

1

1

131

2112

11

11

1

11
00

11

0121020
2462141

331

1

1

2111

0
1

1

11
01

1

412451
412430

2

1

0
1

1

712391132432152103141
2100000100114062020

1

1111

41

11

4139112141173112

5915510555141283281316662209451056281638443421314832029184
000000324200310243111190022210532110190

2

141328241121

11

5915510513101031112313711316154131

4

1

1

12

283172211

1

5511326211171

2

1

3

421

11

1111211

11

1

3113211112113

11

0000100066100
381131011113619711

3

1

11121

38113101061

02211691271116113221432112121
24943221071671319145232872413144

2

2121

2491113442121443123

4

00000031121030112172323904171220230030
14526921411391494334313643252649732415173230812443243

315111

91111621129743993

3621

21

14526921411349111211123192545151133411

0211000000100010100
1404121711125212375241

13441

1

1122231

1

14027112131

11137631
00000000

11137631

014131202011102
78141412121323283

78112181

12

11

210100010000000
721111211212212

1

11

21

2

11111

2112111

00000000005152012301000
1661396779382312210151913472432112

139677915141411461311

1

166372312211

1222611
0000000

2211

126

5350863965019266686801723772081151784528483140242077069196557106695074111321162423604992555348975145388796
000000000000001120140001021301233120204211422200120

000000000000015171811015841233112622594112422414177911153161202256356113332
535086396501926668660572320811517831252817392119558651252548357386692303141118203302144646674444368096

1

411

12

11

2

111

1

331

11

1111

1

53635066243823011303121834211271162

1512114652434

6212

1121111

11

1

1

1

12

1121

2231

14

111

211

48967211712211441047234211243216422

5810351011131123141261361185

1212

1

14611612211112

121111

2912152

11

1921584677713195228214131117

14111

41221

162

1

174611113118535011113

1

222

111392421

180182

17122925641112456133112143121356

2021121

1

2

351208322411334951828629

121

3335

12

2141112121

1

1
0

1

19677132131211453221095181621153614827528715
001311041013121107417102542812220517614

11

2

1121

196772132211468206111

722

121

0000000000000000000000000000000018311115585331827521111172108581873262331961056768543027283103165210813572231
9905239244512113455456238452140146917505215175211157731438242994410629116372588115064982132276548101161149662238757296048142012530506338417783305842082191081893122812103913033774516844721386344111202919511733698650828424382325439132

12813413931403502151702495152772148856280263530122347020918306120571412414147552599581142418217317593742112112222431713010552724
0000000000000000000021230001101300013200110151030200

0000000000100
1341061468119212213

134106146811922111

2

1

0000000000000000020121111510718140313217220123526720101005
12813931403501596495152772148856280263530122347020918305891049314141528765310523171968168737419981151639168228522424

1

21631

1

25

3152111311133

38118422232411352131142811711133

14014842804366775125114638912522242435214611861

12111822

2

31

44782

1111

12834735074621543721481879434724915322221393243291410138954211112825531921819

11411911

2

6834692802634704364542111132432310221124271

11111

363314111212

1

24

87625

11

122313

222139

121122

12

1

11

24421311111
00000000000

24421311111

215216442111184113
000000000100000

215216442118311

1

13

001
121

12

70210
82211

1

1

11

0000000000000000
50192363224201133314

50192363224201133314
0000000000000000

50192363224201133314

000000001516412002001020110100007201000200
5445402412024382086442661311114817132985526251511112522351272911213121112229

0002912430181771016420155221637126417412258544
3924203818124911113617122642218634463222386219153101010207

11

1

1

1121

211

1

11

1111

1

2

2

3924203171441151

11

11

12

21

2

1

1

1

1

1

1

1

11

2

1

11

1

11

1

2

1

1

2978614123151

1

12

1

122

1

13

412111331

1

1

2

14
00

14

0112110
1113111

1

11

112100
112111

11

0
1

1

000000010000000000000000002000
5445402481383061012812318181067521230525112

1211111011

2321311511

6121111

5445402469382434143081810562111244221

1

1

967923673449751344545523845747132917069106435742375229904106051124917321150345821320135412010388496621917363860481420117005043384136012231988147712012914830451061322043081908784021712030484115337147654718436354
000000000000000000000000000000242671459012951785411114121170336401531015480

0
1

1

0000000
1251111

1251111

0000000000000000000000000000003112362111012513421341019243437531233715
96792354044975134454552384574712921706910643574237522990410605112381732115034582132013540321038849662191736386048142011700504338412996329566137551479135149921201572841638283216532022387103253739453017535049

21111

2111613

11

11411

1

233111

111111

132232111112

1

1

1

1123

11

111

2

11

3142111

4251114171282124224

1

532131393312126232621251542615

1

1

111143

3112316

35644511141134111115224

1

9679235404497513445455238457477601706910643574237522990410605112381732115034582132013170751038849662191736386048142011700504338412714728761125461040133838689131273141778271320141625290151832181916726739

1

2

52211113
01000000

1

1

41

12113

11111
00000

1111

1

000
111

1

11

2
3

1

1
0

1

4131
0000

2131

2

000
351

351

11111

0
1

1

312111111
000000000

211

2

11111

1

3141241943
2000030112

1141211831

11111222111211611
0000000000000

11111222111211611

00
21

21

0
2

2

132
000

13

2

000009000000
4112211211111

4122121111

11

1

22
00

22

1
0

1

0
1

1

0
1

1

112
122

1

0
3

3

133151113
010003

1

133141

1

11
00

11

1
0

1

21111311
01000000

2111311

0
1

1

1

174
000

1

173

1212814432331010112
1212815433454213212

1

1

1

123221

25521111471211
00000001100000

111

1

155211111

136

3777443111321333511411312
000010001000000010000

11

37773421131133351141312

0000
1111

1111

0101000000
1111221111

1122111

1

0
1

1

1022741232513221241033733428673423511
0011002400002202100101010010

00
12

2

1

1
0

1

1
0

1

212
000

2

12

0201172102635332062721210
102223237212836334163123211

1025211214

1

1

2115

32111

1

11

1

000133000
27211323112

271811

1

2121

000000600000000000000000000000030000
441210101865507151010196727426231712138441353131

18111115110111
01000003100

2

18111131711

0010
1111

1

11

4412101065447151010195627415132213733112131
000000110000000400000000080001100

2

12221

1

211

441210106544612101019562241512653111

1

1
0

1

0000000000000000
3021212126131923722

000000
30613151

30613151

00000000100101
152121161413722

121

91121213411

411

21312

12
00

00
12

12

40003102121103441080
41123412121323461192

112

2

2

312211

00000000200
741243111521

11
00

11

00
21

21

000000
1131011

129

11111

111111
000000

111111

000000
332111

332111

25186191112263511113734222111
000000000000000000000000

25186191112263511113734222111
001000061010001021102110

1111121112

861220241611

25118

2

00
13

13

0
1

1
0

0
1

0
1

1

0000000000004290005025114017291922016610011639287351901002
32865274434488191397031327406422253935516101092010319621241132691461127187671019172913331278325421073653252918

221463211154794496921203475114381416210
000000000000000000000000000000

221463211154794496921203475114381416210
010000000000001000000000000000

62147132422141215632
52147002302011135002

1121

111323

533

2

2275111584191114152227632111428
041118500000105002250000011

11

22317391128112321146

11

3113

1

211211

11124534332
01123204222

13111

1

11

654034466300591615603322829961708542422822119382121
00000000000000000000000000000

00000005240141215623353210165100920010
654034466300591615603322829961708542422822119382121

65403446630059163151116832711331111271017342111
0000001235205115623110750420001

65403446630059410916321151112051332211

1

72137601124221236232
5150554716220227211

11

2632548192

32827042219136703132681642532125138935148582060236364378033412111711413246018108604508255
00000000012000000000014241140020026874112180000

328270413209313268120276168314415812122270291161827311106992369126656019715
0000001250000000013152694110427445010701031780000

0000171032105224627124029159430253501
58311164202111521452347682292474815415016611

27011164203191414121112411

313141361

2

112112611211

1

401638318496111361742223548802181
000000000000000000000

401638318496111361742223548802181

841211212116910512
011050001548210

8411612114232

11

328266410904270356812431353116398343515811286230931
000000000000000000000000

328266410904270356812431353116398343515811286230931

000002000000001102010000010000010
22194614425339559211077446934257015238644617532631124

12231146
00000000

12231146

251222103830221462421331530
252222133830221462421331531

1

13

112
000

112

22194614425563372197333971141118540616203123
0000050000000000350000100283161300

2219461442561373731711411851143123

22163397

109425112526207511344154047252318395371201413107912611742439142753
00000000000000000000000000000000000

109425112526207511344154047252318395371201413107912611742439142753
00010200021251234112142420101217006701

10942511078122132437157131014414543469126114145741
00010000032151401112011126102210100

0000010122115930122011900123
1092130987111225106111822221319551024

11

12

6

213041491215211353

1099461531

0
1

1

0
2

2

1131
0000

1131

0000000000000000000
21215118112181114212111

21215118112181114212111

411
000

411

181211171111
080111171001

11

111

0000000000000
3111111811211

3111111811211

10200001000001302000300010001
9693289697284131918436318911031211

13201001216220
333122314310361

111121

13111

1112212

285113556213122511
210113332013011401

75142111

1211

0000000000
1128111111

1128111111

413222161328222761412
000000000000000000000

413222161328222761412

00000000000000000000000000
812821104192715446108436333461725211

812821104192715446108436333461725211
7128209317261534577333332411724211

121

1

1111111311115

602489622340910805442587084045715263444950123476018599898417854340493959758645758341154523582148773080562316623593110747008107551199111648528084905533391412144421804964093592546323865827119925218121803743376544134531764620308477174001456738298178521262637950279852000515887477164445832268103847614217
00000000000000000000000000000000192141120623420172336967155711533367545858686725765363598129347111341736529

131114117721121321152114145141
000000000100000000020000

1311141076211112115114122141
0000000000000000000000

1311141076211112115114122141
0000000000000000000000

1311141076211112115114122141

11323
00000

1112
0000

1112

000
221

221

209299130113303285211835292243151324679641128103137253265
000000002010000000000000000000000

113561112151318496211157313741115
00000000000000000000100000

00000000000000000
11332812318311111936

11332812318311111936

0000000000
2211162511

2211162511

11
00

11

1415511755
0011000000

14451754

1

1

00
21

21

22131421
22011420

11

11

000000000000000000000
2092991303032852118282310316270213321215

000000000000000000000
2092991303032852118282310316270213321215

2092991303032852118282310316270213321215

2511111316101192414211111432102192212333104
0000000000000000000000100000000

00000
13611

00000
13611

13611

000000000000000000000000000000
251111131610119241421111132102121112333104

000000000000000000000000000000
251111131610119241421111132102121112333104

251111131610119241421111132102121112333104

000000000
311112216

000000000
311112216

000000000
311112216

311112216

00000000000000000000000000000000000
192315236283324343231163531115129624126212244863591214

000000000000000
161026212111861613

16626111861613
000000000000

16626111861613

411111
000000

411111

0000000000000000
191334314113114122

191334314113114122

00000
628119427

00000
628119427

628119427

0000003010100100000100000000
23152317134612912126622121185156178714

000012061130701003119162002
2315231713162812125622121175156178714

13311611

23152317111781153111215464116712

21111132111221
00000000000000

00000000000000
21111132111221

21101122111221
21111132111221

1

1

000000000000000000000000000000006352495093131603831914416923241915354737552186276725243860378163142916061298418531599742177569711412277529581568
578386632327910805442587084045715013444950123474748599868117854340493959758600758221154523571148773080562246623363110746364107551190611648526904904533391385142541789263333542544023665799219839217281783642446298134001755020246476033993056664289678231254937322275911989415112470562545784265833838814179

6118169244203536891043021311821471271014851110944115353384884279524417429216203
000000000000000001001215110100000001001100300

2818169277302115182123106913849843994473277121312314212203
00000000000000000000000000000000000000

2818169277302115182123106913849843994473277121312314212203

111
000

111

334420353689271624211311525288081123314141
00000000000000000000000000

334420353689271624211311525288081123314141

19281762712261142231115623
00000000000000000000

19281762712261142231115623

612122113141211
000000000000000

612122113141211
400100100020200

22112111

111

11111

351217220381344373244671667220526116212132552644354
0000000000000020020010102303000100

212113221231
000013110131

21211111

000000
222132

222132

181312122611711
0000000000000

181312122611711

221211920223121225343121110919951912212
000000000000000000000000000

221211920223121225343121110919951912212

11111111
00000000

11111111

0
1

1

133516461111522141131284395142
000000000000000000000000

133516461111522141131284395142

0000000000000000000000
14311921323622124282179241

14201921213621122252169241
14311921323622124282179241

111131

12

0000000000000000000000
2589286917601854410114512309143392

2589286917601854410114512309143392
0000000000000000000000

2589286917601854410114512309143392

334241732548382118612111
11012000130100000200000

111282153
010062131

211

1112

1

21122127

121311221211211
000210201111111

1111

1111

11

00000000
11221241

11221241

1111
0000

1111
0000

1111

162311231161516
0000001120000

0
1

1

000000000000
16221122141516

16221122141516

00000000000000000000000000061162966201511316181727782191691541156833201412195120123623338321514
2273961575473576311153774362485687071621145810042449366021110810112028388021835860489110115558734283284311587862323995205195502149152394451232375163673323911818865

111
000

111

01000010010010000
23211243232195111

21331221511

1

12

11112115

1298123131221152617521141111214
001110001051452100211000

11

311

1121123

12981122211510102111286214

00000000
1422421127

1422421127

0000
1111

1111

0000000
1511111

1511111

13210
0024

11

123

2

000000000000000
16111211127311225

16111211127311225

112
000

112

000000
111111

111111

8168726224111221415731211822
0000000000000000000000

8168726224111221415731211822

0000000000
1321141111

1321141111

0000
1111

1111

11225178811413
00002024500103

1112334313

11211

5
0

5

000104221304012152330205311100
128114851152413165262918106142121411212

128114851121121215431226711

21111

2512132

365281
000000

365281

14
00

14

00
11

11

000000100000000000300000112000110
1114059713763612571212331074416237102231410221195

1114059713763611571213310743132371022213812185

211

2922547093142604431373434917133252392510
0000000000000000000000000

2922547093142604431373434917133252392510

401032017111102214151
621142339111122214151

1

21132

22

1

12111
00000

12111

146991121182117142621115106462
00012020106625001460100

2231

1469911162116111116262

0000
1411

1411

002000000001103102002800000001
1501985524227162237148345591845113165

15019834242261215257531556815113164

11462421123

21164141671621112
000000000111000

111

2116214127131112

13

1

55144818113061218411191451211164441149783953291629310
00681651015451645796825281376718440771418

111

111

1514221

3812365111218154217611

1702124151323162284193211

2112122735111121

1

11

21212

111

113

114111

421614319303114113
000000000000

421614319303114113

111221119114
000000000000

111221119114

00000000000000000000000000000000000000000000000000000
22539105163573501153223891375682232931106578893146211799782028218017316037041926679512413114813144202311712137102178242501324579

22539105163573501153223891375682232931106578893146211799782028218017316037041926679512413114813144202311712137102178242501324579

2172119331
0000102120

1

21721721

00000000000000000000000000000000000000
2051573155472933174511191211423262053814328511263135121227

2051573155472933174511191211423262053814328511263135121227

13511111029184147296312468541395133145576
000000100001111000100000000

1351111102918414528631457541383122145576

12

111

14511
00000

14511

20322321843423151321306233372
000000000000000000000000000

20322321843423151321306233372
20322321843423151321296233372

1

5161181119712
5161121116712

13

5

62111315
00000000

00000000
62111315

62111315

956653791091733231201151928881114161321241331711118101
000000000000000000000000000000000000

956653791091733231201151928881114161321241331711118101
000000000000000000000000000000000000

956653791091733231201151928881114161321241331711118101

000000
114111

000000
114111

114111

4201102676534728210746810939622170190631171076249232387850788210915635040121316646393848444331721263010445604655
000000000000852257711914712544223640494410915977522018921226176347913342126718

270282107172705191111317292926119261121110
0000000000000000000000000000

270282107172705191111317292926119261121110

0000000000
1111122212

1111122212

00
11

11

000000
141211

141211

00000000000000000000000
62285711912132541062311171311159

62285711912132541062311171311159

00021410200004120
3326521112221219452

13121812131

62

21239226122

00000000000000000
13113442132243123

13113442132243123

2112110200200160
2212111211221162

2

1112

1

1

77171522566115413141915599812643531531417264623175133101116
04617436174913158537110233431231314183320111327211

6221111131

31111310614312141211

1

112

32259343118

46774124412411096214813354

1211301005024
1211352156134

51

51111

0000
651191

651191

11213583432215724
00000000000000000

11213583432215724

111121
000000

111121

22272111319106531111337135
011000100056031004100002

71211311

2112111333221723733

000000
117341311

117341311

7123111111
00000000

7123111111

0
1

1

1211
0100

111

1

0000000000
1121221421

1121221421

21211211211524
00000000000000

21211211211524

115232
000000

115232

12211111
00000000

12211111

000000000000000000000000000000
3581105329480296221311551110163412113535324114152

3581105329480296221311551110163412113535324114152

11562112101124321861221
00000000000000000000

11562112101124321861221

11211121
00000000

11211121

312944358357376474735636533161111913
3121045368357581535640636537181121914

1125582421

11

12

11

7616634192361
0000000000000

7616634192361

832421112314611
000000000000000

832421112314611

212111437
000000000

212111437

12131111
00000000

00000000
12131111

12131111

0000000000000000000000000000000000110261244667358136294828634226537754111091214914827328269252774027398160269
5314738791875731344033065664419425445053578858475433144872973436255563437493411528233811392328439316975135928006404399724535810611467564723533011242341972823541267136339530561814391729016082788827811792154004181235154499382208619189182097215713159499044312033594453189133377012241

0000000000000162933222022120027310
27393371135427221912532140281965131663217529254102

27792722125325614424441205227192

3925811354191191915925523711

5314738791875731344033065661719425445053578858475394141502962136201563167493411528233811392328439316755135928006404399724533910611467564723533011242341962723521261135039059554814031715315792700771111354150844140434607488232193604287702067815424158758790303432944426185113360011970
0000000000000000000000000000000000000000000000000000000000000000

5314738791875731344033065661719425445053578858475394141502962136201563167493411528233811392328439316755135928006404399724533910611467564723533011242341962723521261135039059554814031715315792700771111354150844140434607488232193604287702067815424158758790303432944426185113360011970

00000000
2732213241

000000
322111

322111

27224
000

27224

0000
1111

1111

0000000000
191258211141

191258211141
0000000000

191258211141

24817475252455933326713718253166966830883163387101839632319232301038271851
00000017012356371318933511385153193371646143672717623181528

7525245331124425715211214582210511
0000000000000000000000000

7525245331124425715211214582210511

41112112711324335221
41111112711324333220

121

000821135316241218011121413513926132305431
248174261121346611167332621424181471813381539354312

6217426121131426321741213113311

191122

1861316141344911

1731121622872204298119156227101158303438463132661191398623804052261752002138212883175989593251247214316114115830438845513150766710199732416139317443916214521348
00000000000000000000000084265317132613013246315043461896126114471814212215074391446713226532515

1141221
0000000

1141221

6154625131371632711323116141621241
00000001201114102024400200

2111

612131241611511414

54623142219101221

422122413614211111211111
40112241251321118211001

111111

1112

111272941952131061117517316551337273
111272941952131061117520316551337273

3

23
00

23

112162287197819611915622710115830343846313266111234438040522617520010151555615065122138523779111661261992503217527363364753180362361022921217915424
0000000000000000000000631727161616012762721354029621271321632614152049394130111828019130532720

11121

741113121121

11

231

11112111

3

225111121181

1

111121112221124

11

12

2

1111211317

411121222131

1231214251

31111221

565680321672311334824521122711492

1

13211133161

111

155111214211242

13212

162142

354112111281

2123

1

141112112141221

11

11411612

21112211211234238111

1121

124

2204323442411759231422119214421225413910

41

1

781111121114314

115214

1

2115

11216228719041961191562271011023033821631326654138016422620062173013047473321625631619397153377510619537206256531083

121114111

12131221

21

3113112431226141

1

1123

1

2122212

0000
1112

1112

1002010000003
1132111111113

1

131111

11

31111121381111
00000000000000

31111121381111

319274411
000000000

319274411

0000000000000
1121561321143

1121561321143

000000000000000000000
173178791211235343332213013324

1731785412123514132112834

25123221852

000000000000000000000
4841110215713133112311211

4841110215713133112311211

239235132931021291474361315911616716911510677107178292153434010123507612584522213351188527997524658107811015733223043123521241864017928666837
0000000000000000000000003023123580188242222883022321611411619161765194461754

000
211

211

2621111141892
00000000000

2621111141892

11111

2021291111103326317311324616111
0000000000000000000000000

2021291111103326317311324616111

000000
121121

121121

00000000000000000000000000000
921772239213215411382345326331244471113110

921772239213215411382345326331244471113110

1472351329310210914743613159116167169941067710752215343891237666512614521856275064282537634840341911162833159872156312433931
0000000000000000000000041288522825113127151410371513126612121410394110339252617

1112

14723513293102109147290131591161671699410677107522153438912376241915821525131525131026192824191334131655225322217710

1

1461331146331211751323374281164

000000
111111

111111

1844651222553154244132123324595111
03423122053205212111101226445011

221145

182112110

122212121295

00000000000000
71411211231111

71411211231111

00000000000000000000000000000009226014564871619234910989473111623715313827013933273015112457156115961697831202044274311523
24255864221228193292429717391311814487509410658214624012301182395942340613788822599777913627163928829102773151191053174198233901667154282640260268112629128124569849817382115204016018234053821541538072022947275012480287267096360744121018147

92706017116421913183333883718182016141984221522
00031102180317213376160713175522155210811

1117141

1

113554122

9270609104211852211424

3211212211291111

00000000
14513542

14513542

000000000000000000000000000000000000000000000000000
1240105310384171814015862712328167313357223772920363039397551581787643153416312631245537842096182273185111255971265442039764411215

1240105310384171814015862712328167313357223772920363039397551581787643153416312631245537842096182273185111255971265442039764411215

11854719210847776274325706811249114364506610491183319742279182395942340613788822229048893320863535829870676496110361749962688146754774235121796011117911738407672781316165618811314670367345124136687152271135713175918675336395193542886122
00000000000000000000000000000007211727104653299156513334587973894331515073989120311569544672642831059749611755013411311101388405477344269563493

27182593231224336397614651321811733495728293493972331277685986752003021171219831011621431616343689661621910112812243381021261716243259102

65260851472386024971281779475449474740544161319147381663782622792655999200137335524231711314121133121392381421464137222

94378146350349897549911034061057346199469406333154141176389105322624521364425307209159418984193416946111612126149581214638113655937209632472292861131345354402394012

106440941876869572323190756710629122994400907613931527101213135512002111376371679670461939762037345477355316746390239834374313206195691082564234679119612483262792631204329722135263144111742683145380905428815

00322222434200000001000000060
141255332398155518421111312329181

0000000000000000000000000
14122311154103484111131232811

103001
113131

13

1

0000000000000000000000
14122311441031741111123281

14122311441031741111123281

11111111
00000000

00000000
11111111

11111111

13121371214321221114912101

20986661217084764614352545642258230782327190242711123801196653384329225333948779335157787643991015138344221132910
000000000000000291100111418122341023923548938829383122

20988231196461221211626111
000006000021000100

221161

2098823119641261

65191222102271514244021914771111117361468912935233
000000402001100011001000200400000

191222912761231161453211026116796931233

65115111393131997111024

601121689476461435254552480307271699732911362232540202141436335751275547855302804320530121145
000000000003101051179676441328742124312127821251100

60112168945946864522312687749422131812331520483284627683561221
00000000000724018678187401210233112111151240000

0000000209334810003300994660011
11679172115221131442482321231752116191223121111

116791721151352143242113114787411

2211152111133102

485425173444664451115213226111121616541643011211
0000000000000000000000000000

485425173444664451115213226111121616541643011211

11131121011
0010000000

1112
0000

1112

0000000
2112811

2112811

00000001101010101121203200000312030010
17143525455239426221108467191626562006271026341715188334182217545

142781631551112
000000000000000

142781631551112

1435372119523214353
03020213004312

11

123

1435719311

111
000

111

17451522411174101411752011111313124211
00000000000000000000000000000000

17451522411174101411752011111313124211

41221811121111
00000000000000

41221811121111

25523792621062413121155323112126262511124
000093000000020020058111100

295262121315214119

11

1123211

2552841141121342219166414

0000000000
1113441611

1113441611
0000000000

1113441611

000000000000
313722113311

313722113311
000000000000

313722113311

811223
000000

811223
000000

811223

122210164612721114262101
000000000000000000

000000000000000000
122210164612721114262101

000000000000000000
122210164612721114262101

000000000000000000
122210164612721114262101

122210164612721114262101

0000000000000000100000000000812120000
35911958375661180119423213932327207872811217601812258356101818961

000000001100000010000000017188580630000
359119583756611251194201127323262073726102760179825685472513961

00000000000000000000000
3591185735666112511463173111451152818814664410

3591185735666112511463173111451152818814664410
00000000000000000000000

3591185735666112511463173111451152818814664410

000000000000000000000711031061
101190941567323223437159222821072513961

37286111111211271317
0000000000000000

37286111111211271317

6416294957312123327157121488312383
000000000000000000000000000

6416294957312123327157121488312383

55311252116312735
000000000000000

55311252116312735

2661386465162826521132310183395529051877960226173333142024500906053303235267035746203254451318331036332851745005104612203415261384243683482164457100629339318666
00000000000000000000000375916152664977361395541161821218965411100167942496211907779810

000000000000000
204549954462124212

000000000000000
204549954462124212

204549954462124212
000000000000000

204549954462124212
114349854432014212

9111

131

103871311053609726659072193217294109140273640121653759846621050353945725957669890418166655131119411214224804111863364
00000000000000000000000000000000000000000000000

103871311053609726659072193217294109140273640121653759846621050353945725957669890418166655131119411214224804111863364
00000000000000000000000000000000000000000000000

000000000000000000189209486164121063259296325825132020101425226646199166
103871311053609726659072193217294109140273640121653759846621050353945725957669890418166655131119411214224804111863364

62542466223758131364400614766242471104156728143542329466143544211926130047557121111543441861146226109
00000000000000000000000000000000000000000000

62542466223758131364400614766242471104156728143542329466143544211926130047557121111543441861146226109

152120307248412815616154467291783562640914361624273223861391151014
00000000000000000000000000000000

152120307248412815616154467291783562640914361624273223861391151014

000000000000000000000000000000000000000000
26116913612315519816515742326793116525252014919798583714541407877538311123457381620221875

26116913612315519816515742326793116525252014919798583714541407877538311123457381620221875

11322641481821633601323727277845134153664142617239205621448504372652790783151430511284275101759321623252317544325821416651721134170539347181194733
0000000000000000000000000000000000000000000000000000

11322641481821633601323727277845134153664142617239205621448504372652790783151430511284275101759321623252317544325821416651721134170539347181194733
0000000000000000000000000000000000000000000000000000

0000000000000000000000000000000000000000000000000000
11322641481821633601323727277845134153664142617239205621448504372652790783151430511284275101759321623252317544325821416651721134170539347181194733

0000000000000000000000000000000000000000000000000000
11322641481821633601323727277845134153664142617239205621448504372652790783151430511284275101759321623252317544325821416651721134170539347181194733

11322641481821633601323727277845134153664142617239205621448504372652790783151430511284275101759321623252317544325821416651721134170539347181194733

0000000000000000000000000000
26825230919552121316701025220527015249126312161762621045

0000000000000000000000000000
26825230919552121316701025220527015249126312161762621045

26825230919552121316701025220527015249126312161762621045
0000000000000000000000000000

26825230919552121316701025220527015249126312161762621045
0000000000000000000000000000

26825230919552121316701025220527015249126312161762621045

3232113111711583
000000000200000

01118
11118

1

323213114153
00000000000

00000000000
323213114153

313213113141
323213114153

1112

00000000000000000000000000000
35436719131645541839315014616154348557161311211172

0000000001972100120200031350000
35436719131645541839315014616154348557161311211172

3543671913164554181522481241314404723218171
0000000000000000000000000

3543671913164554181522481241314404723218171
0000000001000100000020001

35436719131645541814224812313144047231817

52123111251261311
0000000000000000

42123111251261301
52123111251261311

1

1

25211211625211
00000000000000

25211211625211

00000000000000000000000000000000000
1882152178912111135132462352179197203831232111134138312866219823350

1882152178912111135132462352179197203831232111134138312866219823350
00000000000000000000000000000000000

00000000000000000000000000000000000
1882152178912111135132462352179197203831232111134138312866219823350

00000000000000000000000000000000000
1882152178912111135132462352179197203831232111134138312866219823350

1882152178912111135132462352179197203831232111134138312866219823350

0000000000
144112112114

0000000000
144112112114

0000000000
144112112114

0000000000
144112112114

144112112114

0000000
1211114

0000000
1211114

1211114
0000000

1211114
0000000

1211114

0000000000000000000000042000275772002392192352052182520211
6626762020853281171826288825894322993238981693820457115391729516184372531128192633931988220255273724114724916113763

00000000000000000000000000000000000000000000000000000
662676202085328117182628881879432299323696169382045716361228414169281020481716227616265172182486389131041173461

00000000000000000000000000000000000000000000000000000
662676202085328117182628881879432299323696169382045716361228414169281020481716227616265172182486389131041173461

00000000000000000000000000000000000000000000000000000
662676202085328117182628881879432299323696169382045716361228414169281020481716227616265172182486389131041173461

662676202085328117182628881879432299323696169382045716361228414169281020481716227616265172182486389131041173461
00000000000000000000000000000000000000000000000000000

662676202085328117182628881879432299323696169382045716361228414169281020481716227616265172182486389131041173461

71202515118484628881581253452935221
000000000000000000000000000000

71202515118484628881581253452935221
000000000000000000000000000000

000000000000000000000000000000
71202515118484628881581253452935221

000000000000000000000000000000
71202515118484628881581253452935221

71202515118484628881581253452935221

212216234231641211
000000000000000000

000000000000000000
212216234231641211

000000000000000000
212216234231641211

000000000000000000
212216234231641211

000000000000000000
212216234231641211

212216234231641211

0000000000
11229114119522

11229114119522
0000000000

11229114119522
0000000000

11229114119522
0000000000

11229114119522

1411111
0000000

1411111
0100001

211
000

211
000

000
211

211

0000
1111

0000
1111

0000
1111

1111

399614056482416101381095152096116371031833195372715823152229110413098729267937107106
000000000000000000000000000000000000000000000

399614056482416101381095152096116371031833195372715823152229110413098729267937107106
0000000000000004212102242101310000000023349131302000

00000000000000000000000000000000000000000000
25961402048241610115246152062612374122116184738420152191103107203141645965471

00000000000000000000000000000000000000000000
25961402048241610115246152062612374122116184738420152191103107203141645965471

2596142048241610115246152062612374122116184738420152191103107203141645965471

00000000000000000000000000000
1436238493455108421114461212121450692113335

00000000000000000000000000000
1436238493455108421114461212121450692113335

1436238493455108421114461212121450692113335

000000000
612212121

612212121
000000000

612212121
000000000

000000000
612212121

612212121
000000000

612212121

354725385304716271381662663226044253213717455892559616491733438717530273356851106195373666462281875255703199148951721264546001331835709751121411417741677484624523312374835116180
000000000000000000000000000000000000000000000000000000000000

354725385304716271381662663226044253213717455892559616491733438717530273356851106195373666462281875255703199148951721264546001331835709751121411417741677484624523312374835116180
00000000000000000000000000003139753251471392680281023310714173354132418562071160644676141107

354725385304716271381662663226044253213717455892559616491499438717530273356851106195333576338227813847536165140948920214154851191665349247111190149885038043044224826366795173
0000000000000000000000000000881371619622182420757294974046712233398411111444382468157179169105632961433

541422803896148740961011594564541395421189171894440108123454541011121129971383849255050587
0000000000001381351362111042916778092643476001240762424133520263

0000000000000000000000
547048827622321224141821151164

547048827622321224141821151164

261111587533207513131962321244761591011183
021144433107191035622112447614976120

12113

26184431843274111356

14221038967384096101159456433791513446171415311341411163215752413101
0000000000000000000000000000000000000000

14221038967384096101159456433791513446171415311341411163215752413101

00000000000000000000000000143088862191391883281175186118158373155351326741232
1723461161233812484135387682561371745589255147711683814862961522577877919511617140392286551038331855215765107428763577179622191631401375612229756139

00000000000000000000000000000000000
91185113384918332223215721295745603311066461323210111514

91185113384918332223215721295745603311066461323210111514

17234611612338124841353876825613717446792551292116826844824715223975457172100140273114242973422158391185391293552656969817686727941862122993
0000000000000000000000000000000000000000000000000000000000

17234611612338124841353876825613717446792551292116826844824715223975457172100140273114242973422158391185391293552656969817686727941862122993

000000000000000000000000000000000000000000000000
128237269181332231381540432386334276711725723123412154277012472651535847103525118223913695742352417578735239181

000000000000000000000000000000000000000000000000
128237269181332231381540432386334276711725723123412154277012472651535847103525118223913695742352417578735239181

128237269181332231381540432386334276711725723123412154277012472651535847103525118223913695742352417578735239181

55116427721631624312215524257
00000000000000000000000

55116427721631624312215524257
55116427721631624312115521257

1

12

23495149221128836839120733341718352711
0000000000000000000000

044546180072041233839211099162
23495149221128836839120733341718352711

113125101141
113115101000

1141

2345533111862715311424136822109
0453301156251461002412582299

111

324

1

2341111

113

701498119641642768726344371351961766462351263122157571627456459934616235975969164499204825288237195129431788547262836139479845623573152
000000000000000000000158240165114130019811743266755352352101143475152191073834

26349887926540571108266980312226927255923611169236345115472227829219114765101321524846336833
00000000000024142951000100000000300000006010600

0000000000001231694920506552070514440012618111858161100
59191720152405711082669803122101225505220516633342112471207828189114764101321414844335713

2362513331422122711191421123816110782
0000000000000000000000000

2362513331422122711191421123816110782
021415721013441913206351103212

111

23649187119374351757

1

000000000000117517656000114110184061213105182714314251001
59191484152405711082669803122641814031571114262658529114551012911254680131074226103811

10810651118412173654523141
0000101000010001000010

32422121223121

108741162214355331

15520023391295329992915151713213321254421685171
001533107411682239151100112314194371443130

155451292655212153422221

121111

2004193111111211312

1462781522057126698031228446940245307184141122261711129
0000000000000000000000000000000

1462781522057126698031228446940245307184141122261711129

0000000000000000000
5945511217247111342111121

5945511217247111342111121

20430715911314433251153133312115152
0000000000000000000000

20430715911314433251153133312115152
0000000000000000000000

0000000000000000000000
20430715911314433251153133312115152

20430715911314433251153133312115152

00000000000000000151234610312910201610563012396126010011411
26431715142768726338358887581166461575716227812126610831323728591178057294457961067728136435593130228510231512862145

0000000000002323013086614202120210020187000001211
964276872633835216346571628471021131368654482059357628639144669522161556107

000000000000000000000000000000
29426215663381635571621212182734793026133142872164792

000000000000000000000000000000
29426215663381635571621212182734793026133142872164792

29426215663381635571621212182734793026133142872164792

00000000000000000000000000000
3111516192128171111101147426181217316211114

00000000000000000000000000000
3111516192128171111101147426181217316211114

3111516192128171111101147426181217316211114

0000000000000
321113113722132

321113113722132

36463134174122462315294191332
0000000000000000000000000

36463134174122462315294191332
0011011411241221014011122

551321

364630241115221811

16831715188665181661571791132331021411360505020844807365224492948811516379714112227
00000000000000000000000000000000000000

16831715188665181661571791132331021411360505020844807365224492948811516379714112227
0000000013883192811282413837156325847491943719367951427761090223

10023315188662141661571362212119571251315738460132112154
0000000000000000000000000000000000

10023315188662141661571362212119571251315738460132112154

00000000000000000000
11929322121451631162514

11929322121451631162514

0000000000000000
6543112212118111

6543112212118111

86111531112214111
00000000000000000

86111531112214111

68841851241141211322613334212
0000000001010000000000

18511428212

688411411421222213522

1061941142121112112
000000201201000

106194114112112

711

00000000000000000000000
68893912522228218111351314113

68893912522228218111351314113
00000000000000000000000

68893912522228218111351314113

000000
933211

933211
000000

933211
000000

703111
933211

11

22

1271411112215122
00000000000000

1271411112215122
00000000000000

1271411112215122

00000000000000000000000000000000000000
15212748133431552056141182712181173334254723202613243212347551283224859241327514

15212748133431552056141182712181173334254723202613243212347551283224859241327514
00000000000000000000000000000000000000

15212748133431552056141182712181173334254723202613243212347551283224859241327514
00000012647971000409648535295520002813210121001

0000000000000000000000000
15233432059111395411418142127311

0000000000000000000000000
15233432059111395411418142127311

15233432059111395411418142127311

12748115559923101612176141523213518162099381621465431224952291217512
000335198117148910201532711187233151635456817821351005112

011300
155113421

155421

017110221533513104315503313220
127222112235335363227515594713424

25

12723222223

1

11111

21113123

000000
111114

1

11114

48141213551411251
00000000000000

48141213551411251

0000000
1111121

1111121
0000000

1111121
0000000

1111121
0000000

1111121
0000000

1111121

0000000000000000000000000
842242233212162444232131223

842242233212162444232131223
0000000000000000000000000

432222233212112424132131123
0001000001000000000000000

000001000200000
111112113421111

0000000000
1111131111

1111131111

11122
00010

1112

1

111122
000000

111122

322111112102123511213
0000000000000100000

22111111521230111
22011110521230111

11

0000000
2154212

2154212

1
0

1

4125211
0000000

0020201
4125211

35
00

35

111
000

111

15344016131361132117111448214
00000000000000000000000

00000000000000000000000
15344016131361132117111448214

15344016131361132117111448214
00000000000000000000000

15344016131361132117111448214
00000000000000000000000

00000000000000000000000
15344016131361132117111448214

15344016131361132117111448214

0000000000000000000000000000000131508519297515134572755333242254461626121617430587583445314359420
552037546786293987822613202724127142716106366821842555563281519696451731221799766318429869927602402067246430325240527609302812332901102231612124314613236132566350686400132161349911915019315031066528443288623513982426402460

0000000000000000
5116211112191101165

312111121415171
000000000000000

312111121415171
000000000000000

312111121415171
000000000000000

312111121415171

0000
1111

0000
1111

0000
1111

1111

194115484
000000000

000000000
194115484

194115484
000000000

194115484

00054780050621340311132140145110403
681842542303914611771343323119316255527737453071767

0000000000000000000000000000
18475343945925111182221232191216224

054021102754105110221130106203
18475343945925111182221232191216224

0000000
2112214

2112214

000000
184118241

184118241

12111111111
00111110101

21

11

421164
001000

425

114

121211111
000000000

121211111

00
11

11

000
131

131

211121
211120

1

0001000000020000010001200000
68253021292313321341661711211410381114

3211413311325221
0000000000010000

0000100000000000
1111413311314221

1111313111114211

221

211
000

211

0061119020001001112140201030
68252718282313317316314181336193

1111
0001

1

11

42413

1
0

1

68161222
000000

68161222

0000
2111

2111

2557912210213
00000000000

2557912210213

111112121
000000000

11112121

1

855210221241941131831016221
00000000000000000000000

00000000000000000000000
855210221241941131831016221

00000000000000000000000
855210221241941131831016221

855210221241941131831016221
2022812114173002770603220

1

65221111

121121111

1321

11

1347926480429324298242558615061656149273895844833205918412941281516857062467112134842972272866528431191538380140115321670557382632519850807859414267344123544
00000000000000000000000000000000000000000000000000000000000

000000000000000000000000000294035147001041951461391130000001531141945800
1347926480429324298242558615061656149273895844833205918412941281516857062467112134842972272866528431191538380140115321670557382632519850807859414267344123544

2157062982923728584262489554683237273571933181541353243012342201647351391411875192184318163
000000000000000698744419112512567225295010103160210151

24136611435234413
07441001140013

244111311

212321844

16665229829228583851885553153237315852079585611221714271304141123941221395
000000000000000000000000000000000000

16665229829228583851885553153237315852079585611221714271304141123941221395

00000000000000000
1207084121147501211764

1207084121147501211764

0000000000000000000000000000
495437416025683274218148814124112161063220461410

495437416025683274218148814124112161063220461410

1252461925181194202174936818311322912652328119131159221112216192223595117
000000000000000000000000000000000000000

1252461925181194202174936818311322912652328119131159221112216192223595117
000000000000000000000000000000000000000

1252461925181194202174936818311322912652328119131159221112216192223595117

000000000000000000000000000000000000000000000000000000000
1120168136118287245241972546449914927389584483318842844589614795816624671980947966241222113130148393681392046455238251217319702567013654610323544

1120168136118287245241972546449914927389584483318842844589614795816624671980947966241222113130148393681392046455238251217319702567013654610323544
000000000000000000000000000000000000000000000000000000000

1120168136118287245241972546449914927389584483318842844589614795816624671980947966241222113130148393681392046455238251217319702567013654610323544

440785175590017123202102720961228143152794895177558397562128765416439443172787160910131541120628571116815672931886146599
0000000000000000033748873110001476116895000000011018751215800

00000002529352110472134186302135036852242
43611397221692964343763111361340102561031215536119132312

436113972216929612103678116176832318756
00000000000000000000000

00000000000000000000000
436113972216929612103678116176832318756

436113972216929612103678116176832318756

00000000000000
44421151126131

44421151126131
11000051010000

31612

22111

31111

00
22

00
22

22

111111313
000000000

111111313
111111000

1

33

182625257222327831112115278518556013323252113221138541086114255562335114242559
0000000000000000000000000000000000000000000

0000000000000000000000000000000000000000000
182625257222327831112115278518556013323252113221138541086114255562335114242559

0000000000000000000000000000000000000000000
182625257222327831112115278518556013323252113221138541086114255562335114242559

182625257222327831112115278518556013323252113221138541086114255562335114242559

2156621564843149175872176911151437677712249818842210985291293911513463313378581315611241848958121543512
00000000000000000000000000000000000000000

2156621564843149175872176911151437677712249818842210985291293911513463313378581315611241848958121543512
00000000000000000000000000000000000000000

2156621564843149175872176911151437677712249818842210985291293911513463313378581315611241848958121543512
000000000000001302917533799936114114451970595103541124183344085532491

155545148669214917587217691115143651771224984711733513428311917936026227214118291021

6011778151116111410162137111471

366520434551161038322613202403104529893149175036010626823990864841873122159467523420564761067155803359145624525456183472324010911927942304534061861742162395305690555444109135183120193542022986486322411137147380
0000000000000000000000000000004218392210696260683841614387899359224031961213785194503341227211200765212284873663224

160105330252281196244872541615352135391072092791018325111509510916261044426915931372525981791955471273625191165212911642
00000000000000971093338740195426218515011839213101348532567822126311822636

281611077875168114543615723858524761725256229821
000011005030220001020111310110104

00140202010200
2110142322112212

2411

311

14521112

21

3228214111221132
1200110110101010

2

1

1

2821

1

141

1

1

1

1

28161107254131319218524178132101012687
00000001000000002000000000

2812114121111212

12512

133817412112241

1215124521182

40107151653121153253

11

11
00

1

1

0010
2111

11

2

00
12

12

00134233011232120407
12225293122232232918

1

1

1

121

23111

1

25112411

1
0

1

00
11

1

1

0
1

1
0

1

00000000035633191052640182518910969491543131052358682916717641412
1053302119624487254500213539831306612215191348444152371321121717767511243364147353784424727511

00
13

13

11121
00021

1

1

1

1
0

1

1

0
1

1

00010100014010000
2133122541319141211

1

2324113121

211151151

1
0

1

00000010
428213112228201

428213112228181

1

933114331352101233112162113
000000000000000000000

933114331352101233112162113

1

21

2111111111
0001010001

1

111

11

11

00
11

11

000001141
111341161

11134

2

00
22

22

11
00

11

9603025935392040282871926315854355031151123254167169264
00001429221531212295412732201481522393129253

11141

11

28656152116

1

1

62330223230025291831331634121292

2133321731

33775239422711023138111

2

1

11252212

1

56103011011
88145111111

314211

1

1
0

1

00
11

11

21
10

1

1

11
00

1

1

0000
4231

3

421

12112
01011

1

111

0
1

1

00004821101137434378602011191012112300
11948725437714271313914154964219261232202012654611

11

1

2

1

221311

1

1

1

1

2

1

31

1

1

1

1

11

1

12

1194872543711241082712323111195112

1

1

4

1

11

0
1

1

2
0

2
0

2

111
000

111
000

111

00100
12113

1
0

1

123
010

111

2

35322121371211275212111
000000000000000000000

35322121371211275212111
001000111020001000000

35612612211

31111

21

11

1

1

1

211

1
0

1

5113001020011011
5436432151121122

00
14

14

222311111
112010101

111

1

1

1

1

0000
1211

1211

11
00

11

111
000

000
111

111

1111
0000

1111
0000

1111

02000000000
12214711111

122711
101000

711

1

2

00000
12111

11

211

000001400
211262671

21126127
20024012

1215

11

1

001101101000
123113111471

11
00

1

1

00
11

11

0
1

1

0000030
1121461

1

111

1

11222

11
00

00
11

11

16052101788321622118974
00000000000000000

16052101788321622118974
00352111200012143

16052612672201221683

131

0000
2111

0000
2111

2111

00000
21431

0001
1131

11

12

12
00

12

0
1

1

1
0

1

1020011000022000
4121112113167611

000000
313111

0000
1311

1311

1

3

0100010
1111122

1
0

1

111
000

1

11

00
12

2

1

0010
2531

2421
2521

1

000000000000000000000000000000000000000000000000000
5113842341748052861765870787122148633987353125751553282985243415928503179785249305489712611522661324362151071928629667

5113842341748052861765870787122148633987353125751553282985243415928503179785249305489712611522661324362151071928629667
000000000000000000031642135516811921149713240002502711221711

001
111

1

1

010
111

1

1

0
1

1

0
1

1

21
00

21

1556211421121131145042
0101002010013001010

1

13

1112

155411503

11

1

1

21

192525211711122811531522151219254
0011200400011010002100009030

2

3121

11

1941111895111210224

2515121

1

00
11

11

101

1
0

1

5113842341748052861765870787122148633987351275328154403637571444516270222628344151854524113241513260512173
00000000000000000211491410818487270931251512224209711109422132015

511384234958052861765870787122148633987351275328732627233052617543213315828296125144194573910158

79617112122

11
00

11

0
1

1

00000000000000000000000000000000000
487056278529773811116521303442781533654116201441711172631051515176149

000210010030121225504302010402120021
487056278529773811116521303442781533654116201441711172631051515176149

4892224221
02200000

121

4

4823

21121

002278284727111145118331267853221121043692101941111168113
7056258429773712135012934027713760361614114151172231031213175128

131

2241234421521122124212

1

1

1211

7056282124111712234612662513

1112615212211

11

1

0000000
2131312

0000000
2131312

2131312
0000000

2131312

341212851
0000000

341212851
0000000

341212851
0000000

11

3422851

1112121111111
0000000000000

1112121111111
1000011000101

10100
11111

111

000
111

1

11

11
00

11

13358102510151204248128574561725101215061346673117176653
00000000000000000000000000000000000

00000105110000001000010000303330000
13358102510151204248128574561725101215061346673117176653

11
00

11

0
1

1

133210
000

133210

11

58102510151164661185745616259121505134664384146653
0000511012141213110000103002330001

132

2

1

111123

1

119211424

1

2

411111

1

1

58102510151235313324711146512544323412

1

1

12

1

111

1

11

1

1

1

2

1

1

11

11

111

1

111

1

1

1

1

1

1

11

1
0

1

26251231121136523
00000000000000000

26251231121136523
00000000000000000

26251231121136523
00000110100000000

12

14241211512

1111

13611

642117111212
000000000000

000000000000
642117111212

12
00

12

6321711112
0000000000

11

632171111

0
1

1

3041032228687714999563158660017927092385081324242012214446223498645125535931666012725132154565766731258812452871771451454126635925571872941266241811074636750257
000000000000000000000000063135223111271391142311017531448110020221421447311133010

000000
231111

000000
231111

231111

000000
321112

321112
221112

1

000000000000000000000000054111146862756149281803813626711102084014241303
304733100187714999539858660017927092385081324242012214432223422945114635918328032218297166314282664189922206452630252591441144981103968126143676201

153477262149397831224127241019136211264025252
00000008785151101000301100

31

1534772621493978269341918941252126372111

2481

1244

11142111
00000000

11142111

5948015461599511854710371370950813242420122663222294511463591449211246062620327142206171161822255104738458943812432536194
00000000000000000000000331010462931202068010315512300507

312132112

321111411

421

594801546159951185471037137095081324242012266322229451146359144615114589211932412920331141815244103734308333611632476183

01
11

1

11
00

11

221120111

1111224111114111
0010200000113000

1211111

113111

121
001

12

922533702804971079234789410550381172052120346753123292911141
0000000517487373197176171633884210317110141

2533703991005311139172360

9111211

11121

92280987423413532720151211128

22132611

0000000
2381133512

2381133512

11111
00000

11111

316451
115200

1211

24

29912852331407571097024072521987161712523597664985174167195121175163835095182302694355
00000023381404028134513159425193137163111024504565352310514047

5681870112591715517317514118231296414327111176328
014589851151551331547381722127501229391164927

27013334111112111

11111

221283

2981562121191146

111

1321111124

0000000000000000000000
2992631691401098272379343871213181712

2992631691401098272379343871213181712

00000000
19081659612

19081659612

1
0

1

0001178374941810179143313011101326139013091271
832641892197433108336207101018481332211193023108548131431

1121

1

11

64113316513

1211

1

1

2

1

111

1113113

1

1

1

1

11

482214

1

11

1

1131421

21312121

1

1

1

21522

2111211

3412111

1

1

1123211262

832189934621111225

11

1
0

1

0000000000000000000000000
30839233633310713441163725164267111

00612301020411002010102000
30839233633310713441163725164267111

0
1

1

00
12

12

110002000101
111114131311

2

1

1

1

1

312

1

1
0

1

111
010

11

1
2

1

1
0

1

0
1

1

00
11

11

30839226502995131473123311
001126942015043101310

22112

308392719173512

11

532121241

181

1

11
00

11

0
1

1

000000000
111111121

000000000
111111121

000000000
111111121

111111121

00000000000000000000000000
136119106218243157257156821312135113642811

1311181042181111572577682131113491402711
136119106218243157257156821312135113642811

123
000

123

2251
1140

1

1

1

1

0
1

1

0
1

1

21

21
20

1

122141
022141

1

000
111

111
000

111
000

111

34317647113910338131382212160
00000000000000000

00000000000000000
34317647113910338131382212160

34317647113910338131382212160
00000000000000000

34317647113910338131382212160

0000000053941664312235028383287957120122602635165701
33612002749675436673099224128437812948132271607918425146165725534714309111548817114646922392135

00000
42311

00000
42311

42311

00253300002000020000000000000000
322717422620521965334494941122783336101107

000
111

111

11224411412324217
00000000000000000

2

111

22341141134217

1

0013352216450321530200148100005
32271437221741843336353112258212837

1

111112

3118

1

212

3211111

11

112

1

1

271516

1

11
00

1

1

0000012412104011734507032520007315011022
3361967366599992598181472121192961310173862042132287358321167

4001
4111

1

1

1121
1122

1

00000321211973201523110163040003033
3361967366599992432333110101232744313120322011120135

511111

1

1

1

2

1

336196736659999235112821212415116

1

1111

2

1

1

11111
00000

11

111

3221121262111121811
2011010160100011700

1

11

111

1

1

1

1

1

2

1

1

1

1

00000000000000
122986183512122112

82483153110122012
122986183512122112

232

1

1

43

132

000000000
211321255

211321255
000000000

211321255

2004780541998514111312311
001003000000000000

2004679531968214111312311
03665461953014011312110

111

1

21

111

1

132

2

2002114

1

41

1

113
000

113

00
11

10
11

1

00000000000000000000000000000000000
242541045031271512617422437143516610515307636813310201719625426

00018121310566805141212030210620310713172427
242541045031271512617422437143516610515307636813310201719625426

111

1

4

1181111

71

1

11

311116115

121

11

11

31731

13

1

18

2211

11

1

1

1

2111

551111232

1

8

71

1

2521

1

1

1

1213

2211111

1

5112416

131

11

2

1

1

61221

11111

111

11

537222131331389853271

1

12

11111

121

1

1111

33

21111

12

211

1

1

2425462110222121371

3233111

2

2

1

11

67271441136214

1

2

111

1

1

22

1

231191231142141332
000000000000000

003000030000022
231191231142141332

110
111

1

01140001013121
2311612311114121

1

23212311

00
11

00
11

00
11

11

958136319532142149109147611691713216882061151084761083126751732423562838169721012184306332485198223519353481345326838732622213220363249552133
0000000000000000000000000000000000000000000000000

958136319532142149109147611691713216882061151084761083126751732423562838169721012184306332485198223519353481345326838732622213220363249552133
0000000000000000000000000000000000000000000000000

958136319532142149109147611691713216882061151084761083126751732423562838169721012184306332485198223519353481345326838732622213220363249552133
00000000000000000272402937711123882523444050881254549139761548664468111744053887913452

000036546193321101787438927671901221876076207113178125121313072207170867313244
754181724234766196528813321636113546234139017818527913412428022125316921852311830910013020210132261

0000149121278184213514544926583211355751254953563117356210606
754667903168161133278192213824714927603512416154255203983518386412756

754667903168121831112643203212215

21631512212

0008264232383430518534332631293143171838649251101950139811
115072230890653335874355245393531373532471819441573531122521511411

23111

30821212

448443133383131115121

702210161

11

31

121111

27928221122

1

1

1117111

111231951

112

000000000000000
798314311213115111

798314311213115111

1363195343109147611691712368820611510893121332371312116171022812939141995192365
00000000000000000000000000000000000000000

1363195343109147611691712368820611510893121332371312116171022812939141995192365
00000000000000000000000000000000000000000

1363195343109147611691712368820611510893121332371312116171022812939141995192365

0000138118310210010021118240193758112675315977276600113431733347015
20428214955718318176103813114122120342774364194078486281400743120643254469015

256312131
000000

256312131

01320211151060591323215023831114811711
14917382231941816349335122734451216201113812

2111131

31

141311312122261111

1111

12151131

11

14947

1

1114

21

151

2272511
0000000

2272511

17541611213114231
00000000000000

17541611213114231

20428212619411723411317214381213847
00000000000000000000000000

20428212619411723411317214381213847

00000000000000000000000000000000
221311415677161851131111101531121174359359262153410222219

0008211100503921054013103038011017
221311415677161851131111101531121174359359262153410222219

0010100000000000000011
124315211225331494108292

231121111124911
210011001000000

10111041
11111141

11

211451
000451

211

20120010100020817
101121122522471818

142121
021021

1

121

800122301
811122331

13

1

1247081631831812221888942010
00000000000000000000

00000010000000000000
1247081631831812221888942010

0000000000
3311322612

3311322612

311162384125
000000000000

311162384125

118697141112108421185
118697151112108421185

1

2
0

2

22153141111225132
0000000000000

0000000000000
22153141111225132

0000000000000
22153141111225132

22153141111225132

116149133131122146615313
000000000000000000

116149133131122146615313
000000000000000000

14813213112211314312
041311311001000210

11

14421131412

0000000
1161136311

1

116113631

1253167979623651650216623615104239928712639168728912891461542511945114050943134905672131775973561360185296439115135136873178047451114445397137510109149746231943392872041121373543586208552681859194434
000000000000000000000000000000349957215001841326431281561101327286335536115961130241449981115

31211831
00000000

00000000
31211831

00000000
31211831

31211831
00000000

31211831

10421504709236515722166236151042399273117785658585328977154251169110884623891490567213171266356114316868593501363012598308787710106842137211018025858174801062372211742103767739395607622441694150373
0000000000000000000000000000002253362046522344933869819374628233274641318219611765921119169322612136

0000000000000000000000000000000000000000000000000000000000
5491025273453251055762332827399270952766260145142465015220173925672131553243564529002191477525633410014112255030730186463148660423497610914405763481799360527201128697309

5491025273453251055762332827399270952766260145142465015220173925672131553243564529002191477525633410014112255030730186463148660423497610914405763481799360527201128697309
000000000000000000000000001142184423880321116361669280129974955321641264070366102130654561234

11

00000000000000000000000000000000000000
60466420313225354690207676148323711319410874142215111321441561092

60466420313225354690207676148323711319410874142215111321441561092

85671211132392921
0000000000000

85671211132392921

00000000
21121125

21121125

38913412312321852228
0000000000000000

38913412312321852228

486810741141042223131114161119193212
000000000000000000000000000

486810741141042223131114161119193212

66373221161121124
000000000000000

66373221161121124

14417915030222432113
00000000000

14417915030222432113

11113256111
00000000000

11

111325611

00000000000000000000
1866958833332185371432178172315

1866958833332185371432178172315

00000000000435438247152273729146117702472973424291637634411984181831822180132551243
32510534847244260914513346302513285374990571562898897511187828234938550917276810412485171932062393142654259

214211

746038212131041334

653221621121

111

112111

3251052742573034091451334630251328268931616625131818353558459541321315

15510113516191221326624

00000000000000000000
8512111112811151628742

8512111112811151628742

0000000000000
614322121114122

614322121114122

0000000000000000000000000000000000000000000000000
29777812345573531951663501051918141521544392491705872428257585872163192130316111232298181713182791152220259625071214

29777812345573531951663501051918141521544392491705872428257585872163192130316111232298181713182791152220259625071214

00000000000000000000
24251184115113353261

01030000000010000011
24251184115113353261

00000
11111

11111

1
0

1

0000000000000
1111713323211

1111713323211

000000000000
122111311213

122111311213

493479436191512471116235584197118514758191229771541062674383101874981594245442842712938222101735524823233491501356516720493140911963382621212816091343026
00000000000000000000000000313946175001228677656201810318337189125363352231813403

0000000
1323112

0000000
1323112

1323112

323539781692916228112223115813345111416131753
000008764100011104000110741091200

00000000000
39484981321291211

39484981321291211

00
11

11

12021232
00000

12021232

0000000000000000000
32351771641111112251353

32351771641111112251353

011
211

2

00000000
3011111221

3011111221

4614794011915124711162355841971185147581912297715410622843823217059815942393373359104322219414348240426430613112457149171781228115128114874542297912520
0000000000000000000000000017718517238501303078837052771023782681649374810735405171315329

0000000000000000
3154062281077571361722111111

3154062281077571361722111111

202328310221310
202328311231322

1

112

000111128120950273524233111
18424976211368622763353624234111

1842497612451813811

347317384516311137448241971185147191229771541062284381651126981594217276935191122231662391512345021758885169428616968201108430208
000000000000000000000000000000000000000000000000000000000

347317384516311137448241971185147191229771541062284381651126981594217276935191122231662391512345021758885169428616968201108430208

00000000002010
43211511117511

113

3311151111251

5853411212481623211
000000000000000

5853411212481623211

91509521111111
000000000001

40264211111

5124551

117511111011301
148611111112311

3111

11

00000000000000000000
214315761236941314211134

214315761236941314211134

000000000
14251119113

14251119113

536215976412
000000000

536215976412

13392961111112
9362550111100

4341112

0000000000000000000000
101874141415361373223721

6910001100110320001300
101874141415361373223721

000000
113112

113112

2212111111
0112111011

211

0
1

1

00000000
11113352

11113352

000
311

311

00000000000
73111111131

73111111131

00000000000000002144006201000211648118121594021311111695
211175877814866028738692545247422633117967482010471548311161179819785575547301847846112184112663346

30100010000010031153514
811121413124613222313314115

100001001111023012
51121313122513181510

311111193
000000000

311111193

1111212416145
1111012415143

12

2

0000000
2112741

2112741
0000000

2112741

221486601453869117524727312134321212131270117249356113311741113
0000000000000000000000000000000000000

221486601453869117524727312134321212131270117249356113311741113
0000000000000000000000000000000000000

221486601453869117524727312134321212131270117249356113311741113
0000000000000000000000000000000000000

221486601453869117524727312134321212131270117249356113311741113

189175877814213739563148623913103611432918268755742192215664477714196591323
000000000000000000000000000000000000000

189175877814213739563148623913103611432918268755742192215664477714196591323
0000000091115423811104178653151419211294022368038715

0000000000000000000
1601483956310937553111141445213

1601483956310937553111141445213

0000000000000000000000000000
29278734103942310164411213154117234263645

29278734103942310164411213154117234263645

44394374343921243282181236216133223
00000000000000000000000000000

44394374343921243282181236216133223

11111114
00000000

00000000
11111114

11111114
00000000

11111114
00000000

11111114

23613434724034769233571751251556564573413042912534261105833001343362034381240092762645915671143114208726573331145161436521020135571076536
0000000000000000000000257161910012041114154351320970132147181201949

1

1

2361343472403476923357175125155656457341304291253426110583275127320184428123889272253451416710810118863587332111493139581921913357886127
0000000000000000000000855482481002351696019784321291861297128725143171728182412

12
00

00
12

00
12

12

0000000000000000000
234182137541691399012180724142113

0000000000000000000
234182137541691399012180724142113

234182137541691399012180724142113
0000000000000000000

234182137541691399012180724142113

000000000000000000010
20411220133151127512241

2011203214127512231
00000000000000000

01000011026510021
2011203214127512231

2012032311221

31111111
00000000

31111111
00000000

31111111

1242114212
0000000000

0000000000
1242114212

0000000000
1242114212

1242114212

000000
211111

211111
000000

000000
211111

211111

1211300171136102214214011231
222232323314811221821132324613

0000
5111

0010
5111

511

13
00

02
13

11

2000201
2211211

00
21

21

00
11

11

0000000000000
2113212131138

1
0

1

00
11

11

212111121138
110000001008

1

111

1

2

1

2

11

1

1
0

1

0
1

1

13144
00000

13144
02014

3

11

1

12115511431151
0000000000000

0000000000000
12115511431151

12115511431151
11114510411151

112

1

11111
00000

000
111

111
001

11

11
00

11
00

1

1

000000000000000000000000004000000040100012321000000
64646858103125155654357341915346341952651492643231251136441645554111362445171113131128822932988

00000000000000000000000000000000000000000000000000
64646858103125155654357341915346341952651492643231251132441645554107362345171111928108722932988

00000
21122

21122

00000000000000000000000000000000000000000000000000
64646858103125155654357341915346341952651492643231251112431645554106362345171111926108722732988

1

646468581031251556543573419137463419526513122332094133555410536224416101002410821202486

1692515111111611

92523514213111131517342

282255772215726079662132821122238187161936146199129641159631844523410102
000000000000000000007000000000000000000000

0000000000
212131016221

212131016221

0000000000000000000
7471412111181356332111

000000000000
74281171323321

74281171323321

010100011211
714131331211

74332

1

0000000000000000000000000000000000
2822557186796621321111223417536112134111857143311631092

11133612195122
00000000000000

1113612195121

31

1112
0000

1112

28225571867966213211233145134111548933143109
000000000000000000000000

28225571867966213211233145134111548933143109

00000000000000000
577225102512478111165

577225102512478111165
00000000000000000

577225102512478111165

20000000000000100000000
113111324821212936414132

000000200010100011
621122372212236422

0000000000
5111723521

1

511172352

10021000
12121221

122

1

11

211
010

1

2

0
1

0
1

1

311114
000000

00
11

11

113
000

113

01
31

3

112121
102120

11
00

11

0
2

0
2

2

00000000000
857033237611521

00000000000
857033237611521

00001000111
857033237611521

1

857032236211

4131

3171111141123
0000000000000

2171
0000

2171
0000

2171

0000000000
1111141123

1111141123
0000000000

1111141123

874545449658549381017152413066133436112221226106114513172
00000020000000021720320100098114410

0000000000000
2121122341111

00
11

1

1

212111234111
010000011100

2211122311

12224123
00000000

12224123

000000000000000110000000000000
874545449658499381015150413064252141215226408121

00
21

21

874545449658499361015150413062242141215226408121
0000002321547015161732701110201613151

2

304529181018132565146112111233

11414

3

111

311

213111

1

4

5745446740105135591481481434

0000000000
1122412121

1122412121
0000000000

0000000000
1122412121

1122412121

62232131111
00000000000

00000000000
62232131111

00000000000
62232131111

62232131111

1121012191272181751116923132011428428629885912625206292046143185591533773189
0000024482714510103146140101000052329111921722

0000463420001122320452004215060241
1121055727172103734715215172230742773509023152793283112735963047165

00000000000000000000000000000
736757297173448236241568231816145123432242

00000000000000000000000000000
736757297173448236241568231816145123432242

736757297173447236241568231116145123432242
736757297173448236241568231816145123432242

17

000000000000000000000000000000000
11210557279530131461813172464223526711254711029772356272382

11210557279530131461813172464223526711254711029772356272382
0000672410113020211175820342668104464920521940121770

1125727172231413432510133
000000001000000000

1125727172231232110133

21

111124

000000000
312311121

312311121

11412131
00000000

11412131

01100001100111
10562111112118111

11

411

1051111117

1

2111222621222
0000000000000

2111222621222

0000110000000000000000000000110
101634313411235431665151156156201025252

0000000000000000000000
10162111214441411011105512

10162111214441411011105512
0000000000000000000000

10162111214441411011105512
0000000000000000000000

10162111214441411011105512

3412112311211123841713
00000000000000000000

00000000000000000000
3412112311211123841713

00000000000000000000
3412112311211123841713

3412112311211123841713

000000000000000
11211121146122213

11211121146122213
11110000146122200

12111
00010

121

1

112
000

112

12217322112193138116814
0000000000000000000

12217322112193138116814
0000000000000000000

0000000000000000000
12217322112193138116814

12217322112193138116814
12207312112192138114003

1

112811

00000000000000000
7211423212212533352

621042321127533352
7211423212212533352

12

113

2591293771584391952682838487861188584596497271545532691503359882244838044681369457611364437359420266703370929184616487884264055456443239864593554281696943610607037391271990205032333729156164287866395215217318609255343756898562512147856210241275327185781597455292637558630269928649024439935300716294545142924393837303121267954485308752571787402826152204789173479304842
0000000000000000000000000000000018582319142519018999554167425790617102081931587364651808162795479360312451825323252922412613930205652037238069211084876421827834113192420276381235824679

000000000000
131172112313

000000000000
131172112313

131172112313
000000000000

131172112313
000000000000

131172112313

000000000000000000000000000000001655370725662107542892466751312691655283072728237721901118527338504470582331133767518190726837763520202234
12104104996481957034841954278846699391161278246240218921167717639455054299239051742452542907969398865919738141936862392782477123157257785054464333184618221340188883099397856201800170399452623161225217611897136020751042464823746072654909203528420889871234

0000000000000000000000000000014899127364012033149303304435598313295726444482294434215830497432416724991332114553464177
1007252952513492424501734341181024274538831064149439227051076385812468013331773475816332911826010222951257545351092448621401308767803139420130481393813116811129255141097293080793127771131219011157307119940011471581390579641

131201251154411612102
010170012000060180

00
13

13

1111121
0000000

1111121

1131239534711
1011225534711

121

1

3

0
1

1

12161232121211

0100000210000141107103000000
32313448642441413112125728111521

11
00

11

2111131411
0110001210

11

213

11

00
52

52

1311121
000000

9121

41

1

0000000000000000000
1112211121224511122

1112211121224511122

0010000
1141441

1131441

1
0

1

00000
11121

11121

0000000000000000
1211252112111132

1211252112111132

11
00

11

1118433463322101523212137
0000000000000000000

1118433463322101523212137

51111111121
11011110100

41121

82117370107791098118314396642367149617716055105675915828776168959740255224121683224423858170122671403330272447792
000000000000030013912742455823882365913177491820717947282722621637897730119512121973071

00010
11111

11

1

1

10429792596224211135173161842052214241612434695193241251014169
0000004910430613216345191154221533138221124

35111214163113123562216113222

111

6929792596222711101322311583126321101201181143

000000000000000313536011100000000100000
4491447010710981143642364969641231425548112232163121431222212163

11111

11

701111122

1

1111111

1

4491441071098114364236496863122111113115312119112153

00
11

11

211311711710412211
00000030030001000

1

1

3

1311

2121119412

1

11

1

1131
0000

1131

1
0

1

26815817117135111025132
00000000000000000

26815817117135111025132

0000
1111

1111

55141151329722462134172012149513631114404115112
551511514316724473135182013149513632194434116113

1111

1721521

11

1311

111
412

31

1121221
0000000

111

11212

60102342310310003310401210101
1033265524126131134713611321312

11

112

1

11

17

11

121111

13

42121221141121

1

11

1

000000000000000000000000007724158032403123422050142427866482926633303542232994794101622724
718947042232465213073624563372575291464864726025491076833690801215972591554020091968314545166680127920010116114170843143921121224430817817016482145456819479713131362925141680204

111
000

111

0000010110
1212116111

12

12141

1

1

123511111211121
124512111221121

111

11122
00000

11122

21012034010301711110
210512134121313911111

1

85111

1

1

1

1

1

1

1

000000000000000000000175410134511630850220148123102302229
420810414861790115136878365816664726055373112214564312341790404314176241392829351972174642205114341468168236153418323714517273052

70154422119337

31111117

8

1

1111

111474

11

3231421

33513111

38851041486179011513687836581666472605537311222784312341246404314176204302128356454240104614239641161125231241088212815

2111

47733420112

1221311212

2

1
0

1

0
1

1

3444611992155345431210845118
000000000011210012110101

341615164123227623

161

331128111

1

31111123

1

1113

1

0041351162942161371591115
3281515111931354212231115101119

328111341214114

1

22

1

313

4001010
4432323

4333

11

00491011201001141121017002
4328610013111311132251223137142

199131191

131

12

11

23386331011152

4211

121

11

1444357717462862156883371792166148219961076102568801512484306219156436912521013312311131342312175179173661990321728310672386158883
00000000000000000000000000000000000000000000000000

1444357717462862156883371792166148219961076102568801512484306219156436912521013312311131342312175179173661990321728310672386158883

00000000
59255411112

59255411112

297111113231132
000000000000

297111113231132

1
0

1

002995131001351537102111305312231032934
5131763305432111761557153111316712831132934

51317629313121461

2112

1

11

11

1

111
000

111

124362312831
00202010000

31111

5216731

4121

000225855113292011314843111913912219571282500
595230213734138751841246111614108411111157143196613102861

111

3111414216

1

11223211

1

23021321121141821

5951232219912411

1

1

123

11211

11

1451331231816111
0000000000000000

44131231651

11321111

199249228127297013023355559917096936376813215628474572081773129976519517345418942293413350476516393913761218260392941431025739114361323936568446327610675193855702214932591
0000000000000000000000000001517048473839164263746393530673244153963318973799720655722614

21011
41111

11

1

32993239287119516889131387922105642475104284124082662833385141922514225
000000065041023651021033351010015105030313

111111

2

313212112

1

1

52121111

411

1

1

29387111611111112211

36062392871195168891313879635222152215196513313211811110121141

12

1

1211

21113

601547628495131621241253963482411
01237526225131411101243763381411

1

1214211

1

11

6031252

74542599320391743823384331103129121407869215714321102111175122222111733
00000000000000000000000000000000000000

74542599320391743823384331103129121407869215714321102111175122222111733

12

00000000000000000000000000
1942142812721556619324142010134283922232364113

1942142812721556619324142010134283922232364113

521341167151522300161122423717136104441355
45831160130521298161122413507136103441354

1

11

4

75111

1

11

2

1

1111

0
1

1

00
11

1

1

00000000000000000001000
14863831142112561243211317313

11

3822125622143

14861141112311323

1

0000000000000000076053001610010030030011301000300
105327839033717044893156281208352279834272601443918033710613718011231346711191210931747794913432191405405715

11111

1

10532782692621702689315621411612768834272608939170194546216032284481113696273523265220191365284610

121751806792225211553621117171518621217125322521148115

11

1

1

11

438411014
0000000

438411014

000
111

111

00000000001211110312311015020110575014
1302599926363264287765154335465310451354357562617322411564011135856149

10775999263632642877651543354656349731553216316113351029641

225126112334

1

1

1

114611

111

709988535812511177817565824910973139135532873739501739897132149211841122912412639
000000000738833001222512011242291023515922451132022014213

0000000000101100
6111181141114117

41111111

217141217

0
1

1

0000003310100121011200
571121745521311153131411

5711111211021211

11161222132

000000000000000
995378311811011111858

995378310811011111858

1

00000000000000
12512562815669212

12512562815669212

0010001711012563
823221233162211328610

14

6

82312221112

113

1

111

112

000001
111111

11

111

311311111111144112
310311000101000002

3

1111

1

11141

88125132163211
000220040210

883112231

121

1261222542741
0010010001200

241111111

11214354

00
11

11

000000000000
58173121224142511

58173121224142511

3
0

3

000000000
64121211122

64121211122

2001000000100
4132121153211

113

111

1

2

1

1131

1

1111

000000000000000000000
7017301139131241612185102

7017301139131241612185102

111
000

111

12213521
00000000

12213521

0000000000000000000000002587889519313596548992000040001140120
34526230327117055710417697224454839153923161713821625566257207563220188110539537483207225016418021659138318616421179261002544012331910810657635

2122232121111024
000000000000100

11121112621
00000000000

11121112621

00000
11123

11123

1112111
0000000

1112111

29726230327117055710417697224454839153923161713821625566257134563215675984170109531131219145166840232511521441211721871802512418916454125
00000000000000000000000070118580523112274231726447355505271007

00000000
31211121

31211121

110
111

1

000000000000000000
1821312663723411101719241

1

2121

182131246372331191719

4

11
00

11

000000000000000000000000000000000000000000000
11526230327112727120634441226534112447134384675312753181781121351111124192221140111223436

11526230327112727120634441226534112447134384675312753181781121351111124192221140111223436

00000
11121

11121

1111
1010

11

3121111
0100000

321111

3
0

3

00000000000000000331100808103000124800001930600033
435571041391516101716551316171382166614256321561022310342874133292651927314109610812764916131046113416

2

1

68241711111441122

1

65511423782891212171511026732441114

1

66411121224102

43557104132351610171171382161932111023183812426131211931274311411

124

1

1591201

59

15748214

6

3111

11122

1

22711

1

1112

0000000000000130008030
107682312511361123521183141

113

1076823125113611212111111

00
11

11

121
000

121

73521234164923412221515113833142
00000011100000000014510040111

111

000000000000000
73125413412211113

73125413412211113

00000
21115

1113

22

12
00

1

2

122121111
000000000

122121111

111
110

1

0000
2181

218

1

112
000

112

48151521325321730181259621523167594745281113110
000001125318725171158620223157584542180013010

331212

412111
010001

1

421

1211111
0010000

11

121

1

00000000
4810322121

4810322

21

1

160548183602437586413979501067021652937388013383077754316286911041430310411644487230126842355368141239747818011917921817141213793683953517833745120412723581722521575971623415423369745274915426804322163261782117309
0000000000000000000000000000001874843493397867601763183329415119394635517215179537310931200632114364653301741

31522314005010112615216513167721322
72022615116119122720308613228772522

13211151452151111
11201020120151101

1311

1

1113211

13
00

13

32114111141
00000000000

32114111141

1221
0000

1221
0000

1221

002001007100101000000031002
748675532222224388653112505214

00000
61111

61111

312413211
000000000

312413211

000000000000
74216712111121

74216712111121

43413121112111281111
01000021112011261101

414131

12

11

1001102110001
1115112221163

111

1112

12116

00000000000000000000000000000014834325625188119821642123236179543240349271150142138112214712721501592355851744101
1605481836024375864139795010670216529373880133830777543162869110414303104116444872301261023553681412397478180119159013071054103227932428460475618678542161172011205769435323361295634238643350502811502521450100266

7210641111111
00000000000

7210641111111

103740922903268445313973805990107514562092235185366150258516574273337349909741673133201150349537253257784691025732559840152695571041476291011
000000000000000000001456014894165068611677233370356573115348164442912105038585522025

4192049142514381915412972395636670126110577878863567374168120111205165173467277913101981641621301616629829351234442734

618204314781246262856833595439786831130108279616229921052179310387843761129053864571117576425253171712482252

35724214034910151921934211072113037431471314549173111165
0000410161453718510026331086191279210102

3572421403493333311132721212205563

11111

111111

222122112641411115

2211

283141487457121121421136112
001001000000001010000

1

1

1121411

2831414774371136112

213726699169141157043231090148117881103122409281422846971430310410244487230122612355368133812418011912426308242348865658991751557252206143127200168406159289171251201092238149
000000000000000000000000000000000000000000000000000000000000

213726699169141157043231090148117881103122409281422846971430310410244487230122612355368133812418011912426308242348865658991751557252206143127200168406159289171251201092238149

0000000
1111221

1111221

12111
00000

00000
12111

12111
00000

12111

36224142332181
00000000000000

36224142332181

12121
00011

2

111

141111
000011

1411

0000000015118577196454390514101101150168211217702
821246314210274272239116974720352020157171727135215466883371865102523675257866735311214

0000000000100000000
9125311921211223152

121111
000000

121111

0000000000
2141181111

2141181111

711112112151
000112012000

61

11

1115

0000120055531010122726803202142221410
1241022144854755131091126127443322319424210712937118

1001121111
1113121111

112

00008141000112110000300001010
124214415145115141546215221811761393

1242144383151213221522111176183

151232

14

10211611611117143
00000000000000

10211611611117143

112
012

1

000000
211111

211111

2136111122
0010000001

21

21261111

14254242224563251821824116713
0100001000000001002110000

010100
131111

11

121

4113121711
0000000000

4113121711

2115426311114611
1000010011010000

111

1154163146

11
00

11

000000
111111

111111

142114125811
000000000

142114125811

004330200310220001000004014000000
822396016816221131318224104232620336221191111

01892110154211102501
824692111154311102511

1

8228

11

23910433227211122494232213221211176
07101217110022183131192101202

111

11111

2391121112119172

22131

21

1

1096471542111342126436630103
0000000000000000000000

109626143211133226235630103
0515030101012114326417103

10912111110716

111111219217

21111121
00000000

21111121

0000000000000000000000000
631171376112379511348212222131

0712130000103160304110011
631171376112379511348212222131

2513127451112

6321103311235918111

5311922413111114134163511
0021061011040001300

5311914234153111
00000000000

5311914234153111

11
00

11

1
0

1

0
2

2

201
211

1

212111231
000000000

000000000
212111231

212111231

5191513576227307115460529215116841517990832383178123513076410206129288171451989361346656670281627163838221809665257381787655928113132116706158461515056256227426727942624815912524925598214281127137994016989902443365473761120923927382821315588174252120675649317503815395722846872255167269237494731262310328015173150585426514962328983200
00000000000000000000000000000000103161993534744199924277166040521563674221887527846261696409360834134220091380514889210833753118357443203551374228222634938293501135513053488316673

28122976742217151242112229588361263066731197574080569213325401325454369684970312328904674711630816081611564000814628203316220489147001634822523105611210514462570490063202315110789200637476119196045819230380976304230285125965591988237165332496443911140101536158388366241356219633587944272668158932510284691099947046
00000000000000000000000000000000150084695412801264493113193834503034252104792349437768164906964350358444434571977826251707328489347132354377241287831463036046663490130115786618727066

0000000000000000000000000000000026232178314834118151498171840521044445938060124268312203442070643979167837324384187172655372156427505214780516506372687117309868
9254606761168937598611281476511191339558926149482196916677601212441922595008050203573132364191596912633549154062984156711507202710911692440252814541412667619890174803031262161141585463919375158421019631678149604575629025921569625899373673227333572135564287219646299638255512933

121
000

121

111111135121111111
000000000000000000

111111135121111111

21071823130514739488205506948218351618714121223327623156117331413231977
0000000000000000000000000000000000

21071823130514739488205506948218351618714121223327623156117331413231977

0000200000000000000000
3185461212623133132212

3145111126213212111

4152311112

00000000000000000
21212231211111221

21212231211111221

000000000000000000000000000
622716121638233192594365255172

622716121638233192594365255172

000000000000000001000001000000000000000000000000
4066741654938375686474721062530218379837637114024311128321731948165742916354110126372652572129955436229101301586110

406373165219481446028131341421121152345

301493837568647472106253021837983763711402431112832172141322612436265256210995543612896281282105

00000000000000000000000005010012010100100000100100000011
993416070139116056459353143125464623402259204962818189214621139810688312125414193283422921452162112717597125204632141210108414265913214693797

12

71218161150111055142343714722117929317423

28123447013911605645935199312546462340225920496281818921462113981068831212541419216837232045211185231352692113433013121098371521017273396

121137111111
000000000000

121137111111

000000
111312

111312

00000000000000000000000000000000000000000000000000000000
30450341532195947216931415340162101380218153444599683464135715878081020712760627680251607211132549387411318114121732104953111343158173164153968583

30450341532195947216931415340162101380218153444599683464135715878081020712760627680251607211132549387411318114121732104953111343158173164153968583

0000000000000000000000000000000000000000000000000000000
205865428054262183520445388437919314335242697531771787615578309134524295134184260146112131328446152920214909622066944294266393224531674449502485031229142208930171

205865428054262183520445388437919314335242697531771787615578309134524295134184260146112131328446152920214909622066944294266393224531674449502485031229142208930171

931732242111157931761121423161212
00000100000000001143201100101011

111241111412182212111

73521539112111141

11211

225212121111
000000000000

225212121111

0000000000000000000
1113144413812141111

1113144413812141111

00041421119200002100300100000
41394141822420612212815523291543132

41394149141211715520181442132

41111

580336120481070465903117170958882245353233491189190663631368263241650136832613920210554611670810302855109418189180106275105150042156225276266961898614632702015447752117846571396335373
00000000000000000000000000000482827810548166475481264577710280812948623161126281081522853214775

580336120481070465903117170958882245353233491189190663631368263241650136832613920210554611670810302855611361117158109569523092168199164161085798264718443174711969428863188298

421411

00000000000000000000000003982418666160033527914041323145380222236404151422075153736235156210986146822841031639529186759391412
22442218474113383666404499110774160248448517624559198411048186140177791541328197639188046621915442896233724697172951444200515319002342663452151209915563963715841969607043404898375976108957801031465

11111112

2

11111

121

16007195183763679192518230211113419102614

11

197852667910771207332812229101322211843121111

11

4994235105624161112

111

11111111221

228411711213511

21

411412112452

21122

11

1111

11

131

111

2111211131

5921141206502271111241542311421111628

15715754092813336911421714253013311221241

231

15113

111

6312576270159455421311537120911111133361113166942262

2024174005760717777602898402909254638115743809595147532816113913461541812961440489339325202581623216381824248354213923014

1311

1

2111112

82

1106902642335721137939762841752879473663658809831034265230626111396721616532027114726211924

40852355223286024727918321545724567222554551111526351392864102666012499511915610812352762524415416414110013174010612223
00000000000000023214309192401178484148107537391132901021409813527105029

4083332592247183213657245672225545511115263511236712136614615743341417153421211611199

1903522322682791804369461752153121323311121714

1111

1448283455953128135197019944492265268313148556216351724194667852842321417333276105211128565281818622
000000000000000000000000000000000000000000000

1448283455953128135197019944492265268313148556216351724194667852842321417333276105211128565281818622

104278922615121795119119821
00000000000000000000

104278922615121795119119821

00000
41123

41123

24448068021223452720125907183274725068518625952431434892689759144343185342641173495124687462542911333123648324415343416227179125
0000000000000000000000000000000000000000000000000000000

24448068021223452720125907183274725068518625952431434892689759144343185342641173495124687462542911333123648324415343416227179125

000000000000000000000000000000000000000
35191827797680327218891830132175615264915382115623364144134448376676214144

35191827797680327218891830132175615264915382115623364144134448376676214144

18517950454335512433157375131320231131155
0000000000000000000000000000000

18517950454335512433157375131320231131155

10993654012181411534311487131352238
000000000000000000000

10993654012181411534311487131352238

621421123117213211
000000000000000000

621421123117213211

3242111
0000000

3242111

1112117112115
0000000000000

1112117112115

0000000000000000000000000000000
2451281158797782957106327516158111223347534171

2451281158797782957106327516158111223347534171

102111111121
122111111141

11

11

00000000000000000000
22121764312311313111

11111

221216643123112211

00000
21111

21111

1834496112248206334102343241045262251782024211511
000000000100000000000001000000

1832486111182711011611115

2011224820633395152334212417820231111

23852615714381657115925017188614231361713263213334317521711231
00000000000000000000000000001000000000

238526157143816571159250161375142126171326321223331551171131

1511311111111

23824431348117512990306210165122217617848411625231331513615728179
000000000000000000000000000000000

23824431348117512990306210165122217617848411625231331513615728179

135838366326998107150735119411183510312252321721054311661
0000000000000000000000000000000000

135838366326998107150735119411183510312252321721054311661

000000000000
107819132111111

107819132111111

1493132391511696785103015411121012676101512110314452
000000000000000000000000000

1493132391511696785103015411121012676101512110314452

00000000000000000000000000
14458121127971335231221261011

14458121127971335231221261011

00000
11611

11611

0000000000000000000000
62926121171752141111251141

6222112111141

924111765212111411

000000000000000000000000000000001463538722438933914702903103948104521594334149363119368916082215528068587685473722699791325356331851743013610126022800
188683699810482248225110144071143932776108312585747244158637242419277624608122484026390849859662451872737591371492532363324354944321116114132718523301655516433544132881657131021775103522360522894323599246512743077522440981612219375824283143026349310531831378748412941439580304522577047

62147665612479824921111924719511824061247611295782
0000000000000000000000000000000000

62147665612479824921111924719511824061247611295782

52354333305135013741978125541147752131868135274891865424181863460
000000453232553012141251271561715324151391330

41754333305135013741978652261822145574222512471428

106015142111111172

1

6919202916506528170570354312587518385295101138827092627113069772301818699102353923154624160323864530613167158115393394420896243735327913191428510192311891861839894713664400112820683547139914548582312515947658288322806524068310343314661452306242957965019215838801752
000000000000000000000000000000003715946628908176663624441319354291412042369120911518012226715605601986272759720907061262510799508511924462751541416518791

4077111612142311421

26791033761508256264510328210873144464406763581999916278681777293157615592261872711171345322176105243408217585528453415784467388817307202441147380146246252227267192162019

2191350511668938201129713176461292191141912571844555331283711230656463131233132108374912125664

11

2211

225111

3900995435572544590543125841192765919610942226917907724977313903108025066552144763269721072175326487981023112520522571306753328830093703123354141348191105152183335351346045671125592021732398210998629822135268877

4559121331013312

189432374211633332421

3406031171618021103265845335865433171132466158615286318123914161

111111262
000000000

111111262

0000000000000000000000000
3675861371326313423262132624652168

3675861371326313423262132624652168

6014563474756894371167312906610750251238543172102808955407115785014154043393387545232382814120280900
000000027290659144971552112992808854399113755013553737375316740190352813114276863

11

60132034747568928844212320819364122225453114111

1

14

1

43723219164141134946536313

3

11112

11

1368795121122121132223

110111
111111

1

00000000
25222112

25222112

000000000000000000000
46749412112211483422211108

46749412112211483422211108

00032276641326556318145128856893511174820284315229121235152
75434861076345917487326766619157149867093511175020314417329121235152

1

11121

16113111

754348610763010245731211

1111

12

1

1645121784363845267217145541552752289741291412993311852213137
00000000000000000000000000000000000000

1645121784363845267217145541552752289741291412993311852213137

7577193511355919963122263175313421371194410172956221
0000000000000000000000000000000000

7577193511355919963122263175313421371194410172956221

45321389111273251533225465371
000000000000000000000

45321389111273251533225465371

915912710644575446382227110437253946420240318113281275942877556134184677586
916912710644575446382227110437253946620240318114351275942877556134184677686

211

17

112211125
110110115

2111

226377323823481683251228111461454513829141012837920
0071236225402201311111014401533831281498724113

41

241112113411

123323

22637131162121321213711287

00
21

21

13135181206337186716813301468616838012922527931823685396312912115253614234996326271373897429877231901689101210
000000000000000000000000000000000000000100000000000

13135181200637186716813306086110380129225279318236853653119111151235138347753161211426943990761518213311099

57865831111314221115231223818835831

10010100000
11123221121

1

1111

1111

1121

000000000000000000000000000000000000000000000000000000000000
1403467224583433155666261128893363412492419727012496214617149174136922391408200825151410154014019139019165371948203113951166131473416218763039342184181096747211733571885142116310134110

1403467224583433155666261128893363412492419727012496214617149174136922391408200825151410154014019139019165371948203113951166131473416218763039342184181096747211733571885142116310134110

409412141921761271262544381747132857231578135195151
000001011100100100000020020200031010

409431131312811

121419217113139281114112434215310413311

132113221

21111111

1511414222311122113

34914362827231121332212110162361
0000000012300201011119150211

14361726111239111215

3491112

000000000000000000000000000000000000000000000000000
64330555712278216413432610814164441013247333506233931997557262487171081652799296416267148151441718221191841052566

64330555712278216413432610814164441013247333506233931997557262487171081652799296416267148151441718221191841052566

000000000000000011010000000000001000000000000000
115517791272142922410736498053436575734673642701629514942213147661075412138141111925717943132981

1111

11551779127214292241073649805343657573467364269161951474223147661075411128141101925717943132981

00000000000000000000000000000000000000
39925235138734424423113166258624823952081961417104211551836511411265121712111031551115215511143

39925235138734424423113166258624823952081961417104211551836511411265121712111031551115215511143

000000000000000000000000
21456122111344523221251421736

21456122111344523221251421736

1941643341141411624318245919
003321000102000010001

28141325

1941646122318145913

325669425456141909376419952215030602054111191544712491724643713223266141177501612457104934660
0000000000000001926000301382091100100000200503

325669425456141909376419952215030602054141216445101524281218326514017746810455104434656

234112

714133312113721

111411

289025818218731781458576575190423434452641221134931141511125284104951233111231496448207782510362326
000000000002397113017011010012003114984340144851154918

13

24

2121

111115

121

141

221

1111411

1

122

28721581821873081145857657519042343445577724185545121832411371117462621043225

1122

2

1

1

12

7097268311299550117173535113231141122

12

112

121411

1

111114611

111

1114
0000

1114

23161121145316113515732010211
0000000000000000000000000

0000000000000000000000000
23161121145316113515732010211

23161121145316113515732010211
0000000000000000000000000

23161121145316113515732010211

0000000000000000010201259531114011011322712754100000
234451638863182218448165656262961152631363687415511031982822427132687094784215232462015

0000000000000000000000000000000000000000000
2518886318221844816561216151021153547640411672222307101364352029211115184

2518886318221844816561216151021153547640411672222307101364352029211115184
00000000000000010010131040000010125100000000

886322816562541145624225139632134201825111

251818184412162151012131110291324471191411011253

156112112131

000000000011005200100003000202
23193363562617451510193322631221231291212131211

00000000000000000000000000000
231933635626174515101832221112112325121212919

231933635626174515101832221112112325121212919

111
000

111

35133117912221063047139230141132965160776236244207173231209481166942152432456113263202232461621102622212109010259388312695150
0000000000000000000000000000000000000000000000000

00000000000000000399114915216008722414427623133343451673267831149
35133117912221063047139230141132965160776236244207173231209481166942152432456113263202232461621102622212109010259388312695150

8024212526983631033373121333342121666
022101717931121011240110131231721351

1

2221221131

2

11141

11

8012151

18674311112134

1

2010242211268541298941531333340237221
12322221126834128194821223340236201

11161112

67111

2611

24370001151205204020310
3581223215112215315541411

1111241

1

1111111

431231115211

65118401914181334131332311511607814107371467441112315
0118371761893011113221129524539531105343192012

1

11

1

1321111

1

111

6513128321116133

11811

11

1

1

1

111

00000000000000000000000000000000000000000000000
35133117912221063047139230141132980776236244366311215540367402571828419813613385357502932071997718

35133117912221063047139230141132980776236244366311215540367402571828419813613385357502932071997718

3744686455602015015720713764121662718534179667818589269290341497352150542332837861014917210656411510465225722513147731314779231
0000000000000000000000000000000000000000000000000000

0000000000000000000000000000000000000000000000000000
3744686455602015015720713764121662718534179667818589269290341497352150542332837861014917210656411510465225722513147731314779231

3744686455602015015720713764121662718534179667818589269290341497352150542332837861014917210656411510465225722513147731314779231
000000000000000000000145231347214724192819295241111255539318724610939872252948842175

941192421313665494237258134265330131112124814174327817619291111016

2803494034291655015714288641216622985341796678148641881118497108652247716303334137525119927165020358482740

143695725302367563280493444596855813238022738394373923534278732622822811358389170910131618257958747392154320051515223227520212763931901308645122614441565450111655973427713812
00000000000000000000000000000000000000000000000000000000

143695725302367563280493444596855813238022738394373923534278732622822811358389170910131618257958747392154320051515223227520212763931901308645122614441565450111655973427713812
000000000000000000000000010237226132602933694387561213377115324126524494723378145821199631607

42114668365727311049124459685581153802273839437392353427873170254031103838916471013115839187160102122216251063139134618889127715488596177593211553346314037624133573
00000000000000000000000000000000000000000000000000000000

42114668365727311049124459685581153802273839437392353427873170254031103838916471013115839187160102122216251063139134618889127715488596177593211553346314037624133573

257585815619315886125927156311121032182112
000000000000000000000000000

257585815619315886125927156311121032182112

49322610328942919221792591179622729041535016111091101223636175211
000000000000000000000000000000

49322610328942919221792591179622729041535016111091101223636175211

265197151220314128551316192151343521223311
0000000000000000000000000

265197151220314128551316192151343521223311

0000000000000000000000000000000024295078585433924891961092668113245451093413847243012621554310390443254787978110542911511420032408411332414711342358304912775758
1525624227385931878748152241221316079112602867252995622981394010269899845672824291271323796956332715311224295872319049035623567506529292824964137683554467951045813921828716645825898034312511808261643278475785013328492167510910103751483430211137841881488587782783277003225396211546681551414324

00000000000000000003551202024530728271126775071322819015713425526472014
30658052743295932751061139417911259464264036776775117108133179522224343101611722381421601021254313131769868379624811474674

48128418254516310045443936711121126133241
3656318254515399454339367115924123120

1

11

1272111721121

131121219213
000000000000

131121219213

35701458424039315768914222491089346423617362853109875532413517222141120
0000034244767802462795873742281633245198530242297121524812

1

21

33261

115

1

35818411811212112318411

16328331313111121633311

11

1

111111

1

1211112

11

706477276113832211631171727

00000000
13211341

13211341

000000000000000000000000000000000000000000000000
30658052392595932751061139434283546426403677203143204323124228902612528838523231621593015221440

30658052392595932751061139434283546426403677203143204323124228902612528838523231621593015221440

0014100100001510300010002304110200
391002775213632414101519319135471575618782881215818

0101160111222174848313922231002
616331935123331118324104152022231114

11

111

11

1

1

1211

11

61147

1

21111211111

1

11

1233341121

1

3939732133153106714145193332922382713
001111038293500338331140211228

1

1

1

1

1

39396237132141111021422755

1221113121156321
1221113120156301

12

11331553211164111214182111
0000000000000000000000

11331553211164111214182111
8281553111154111213172110

12

1

1

22111

1

54274421271180286115111811940121154522628171847242251472205683999226104273835719381611
0000000000000000000000000000000000000000000

00000000000199421411027612237179121152507357832069243414550259
54274421271180286115111811940121154522628171847242251472205683999226104273835719381611

32123132

11

81214134162534

1731151111277401532232231311

54274427118011319182110163124331928537644441101

1223111

11

11

133215111

11

2110

11

000000000000000617712282221193157110177337262401211168803290132513376223514174754
1144014475695894071140258120321244321942711503561061471771473920992961663026024398634195113975017820472156133522440532111133

27112224591202182713141020167118161061461015243231424
01222456113014211013619117660284105914243231423

1

2713623531153123213111

11

11312911

111
000

111

2311131112172
0000000000000

2311131112172

25444741937840784116931507124351194853281219343215355455912493208421614364111411636
0000000000000967801124151451279110402211104156

254447419378407841169315071243511948532831327241423130457366117321212153171130

3

0000000000000000000000000000000000
11414715017329966934381651519919524354070346217821152321267

11414715017329966934381651519919524354070346217821152321267

00000000
41222326

4122226

111

38219182288691131646443431543111110
0000000000000000000000000

38219182288691131646443431543111110

171018311127711107105365610073484142114618204541783102416
13711310026610104103312099724339429451620253146272313

1002362202200110
1112363212211112

111

112

1

426113122221
002000000000

2

422113122221

0000000000
1111112111

1111112111

000000000000000000000
13241161272213118328434

13241161272213118328434
4110140002001012003000

711111521232434

22212133113

000
121

121

0000000000000000
8122122121112322

8022122121112222
8122122121112322

11

00000000000000000000000000000000000000000000000000000000000000
44036488319721861695034224102250635230340818512211415226691965687361620660134289518626781946181908827245082547718333851308111170184114635596118031246118311206516464420731119238

44036488319721861695034224102250635230340818512211415226691965687361620660134289518626781946181908827245082547718333851308111170184114635596118031246118311206516464420731119238
000000000000000000000000000000171245831311719784247237145113648940361134624311511203552

4403648831909186169503416998149555063340818512211412026691965687361571660134283018626781946181677521935469436916142881037844548708382940471322417799243179485183210426110179

635541108167324965618131221324308322115848622313183247

000000000000000000000000
3314123565541811317291131

331012236440160517181101
3314123565541811317291131

6

412112211113

21

4280906211221751513119230019554931667400020445741672198015562566189521514441992052889376461296693464161461125936516391863152131554278677116280111811913848661256987104774074828415511611382462451053169275103180594387
0000000000000000000000000000000010082129106147108125146914105024973210768394465466234788441581131818297298578868280

00
11

11

011000410320001016300002
138241322171671132237352224

2

138215313514112

1

1

121111

111

11

12111111111

0000000000000000000000000000000
1512803064782991294314326624121187241333165421910

1512803064782991294314326624121187241333165421910

0000000000
1246111311

1211311

3611

1

19181521127111928271431201646244280838110719010713294001426142802184125172231746803141441326282743943626601631191871281620241316193330230
00000000000000000000049662027912131750210252022213720432

20143244280289210719142125101551251139119201081113107110149113

1112111112

11

211

31192

137120239412355351431210118113

113221

1

1

111

11111

2224

1

11111

1131131111

1

1

1

11

18981521127111926901646810101519012874006142802184140313371301671392924231416519021361714296

1111111

1313112221

1

22118

216

1

1

1

1

0359212161734108488115212124413433
5565105128671835138489115213225518734

1

224411121111133

551122

1

1111

311111117
0000000

311111117

000000000000000
111273231144147

111273231144147

0000000000000000000000000000688311051121333281113215623210015630212
321404516253313855111103762612211760129913025492205126837661303767030222833912471130442331432835234016424034496351532952777852975274751415429

3214045162533138551111037626122117601299130254922051245376613037670302228314339423342316401218193514342932473301432748736822656183741114923

1415121411121

21112

23812251111121415112

823112311213323

1111139119111
0000000000000

1111139119111

000012035251
121112235251

112

21

1061027199171842232217335146120213415531210
00131201101301172941360121838121110

22111111

111116

21732133

11

10610221681524111

11

233080018215521975403551612374619413781334853112711186152922521858571301156314308368618241821762101402913694413439264512638111075367112173211
0000000000000000000000033713032081152320108104512671616086191

12

2

1

11

2330800182155219754035516123746194137813348531127111861529225218585713011563143042979172179146293382884524124311531945736636162310

1211

00000
112213

112213

00000000000000000000000000000000101413232023153178110477334232757103262801201015218
28177895151007518122950878959017241127011700924995536647347304241849127501660320312253212128601713006131821246199742642142512141518668322033125429060428810319194692964134897684444805717238388751545518258420314395066259187122557468301022

000000000000000000000000000000000000
2646548231334016654214786871014229151412220179322116

2646548231334016654214786871014229151412220179322116

12112122
00000000

12112122

281778951510075181229508789590172411270117009248935366473472686413061261116398199892456120075951127971309212461988326421425121415186681620331229265585260951713458288400473733425472551721928144413841515011781073461535588411755724817996
0000000000000000000000000000000000000000000000000000000000000000

281778951510075181229508789590172411270117009248935366473472686413061261116398199892456120075951127971309212461988326421425121415186681620331229265585260951713458288400473733425472551721928144413841515011781073461535588411755724817996

10233049785205241458866169909186233143713123861001723221014131816212252
0000000000000000000000000000000000000000

10233049785205241458866169909186233143713123861001723221014131816212252

330552974960317324121514418479078313635371013671456393437432459243058281323101353014719009616723893893774183831221462150264028491393163824790737150821702793894100631912642163014392600626631302916499557306434661640910882821352
000000000000000000000000000000005489248775325668190713361739254257169426171845909945113114428810229224132419111261404225679233

4619955236303715125428732816632510774353002621932612161692521316101012232971271391326311832111898982513409234181531
00000000000000059120103749185367194199114594479346741171523148110820

1112112141213

1

52462252104136030019510347191112266344247551

421167231

1

262533523378373625428732816632536756797989070112121232023711534161793011341426

11411

11

421113213111321

1

1

1

1

2

105102511123

1

11551242555214112111121721

51311212121523111
51311212121523110

1

00000000000000000000000000000000000000000000000000000000000000
720122462881911221514416517816342422206872041218385752266831986595775613324656366189736057120319416190109316420578223711909916713179739453291413199101835494852161472344

720122462881911221514416517816342422206872041218385752266831986595775613324656366189736057120319416190109316420578223711909916713179739453291413199101835494852161472344

16187572119235610399473674310241272
00011181133204646246220220262

11

16187571223835301121111021

11

12192714141511572446
00000000000000000000

12192714141511572446

17972978317489441171389412616136804052311651631856218253810881203143033925193555723941518791150199362186262117293470621
0000000000000000000000000000000000000000000000000000

17972978317489441171389412616136804052311651631856218253810881203143033925193555723941518791150199362186262117293470621

000000000
112224242

112224242

000000000000002726141404083718830531131231025112201
1411507151434408138180579970730026210442946449352623342114144847572584040721517435252644171286210

11

45111211111212128

141150715143440813818057997073002621044294641831163121630365425735377013133232623239631

1

111133

8410048583051996619732720850345981674580129648861664529731024210311712415198035624119053941181205214544362108622818174488231343128492185581601063940876248351711361002
00000000000000000000000000000061383844011202869391118825572172650251132848528224346497037565

84100345422517366130218208503439211034377243369118292972221685573613436280261187117831197469174331924281915156016412510546950502184401812626135187879293

140408266710920657114357215174816088744843510715795547272924521041011422266934661651722928141358164982320144

976074799766210123525159130155956423223142415142114481181
0000000000000000000000000000000000000

976074799766210123525159130155956423223142415142114481181

01241230200209021
102251317612332113422

1021285323431

111111

00000000000000000000000000000
318611672311312733633242632111392133

318611672311312733633242632111392133

0000000000000000000000000000000745569866076245571751053197525182651019526215112628213574327380181466178371022663214
615244644963027557157110714424549713771227442987717826966441370312138336332826635718081607482101631592939094450916693404213348214049610332378483151187323396345499385886912471531762321903011279133434562630450723365750

721061075736546612853462348476816272342611370888446169107631721478827422
000000000000000000000000000000000000000000

721061075736546612853462348476816272342611370888446169107631721478827422

00000
31112

31112

362419193161171768113132511141193914
000021200016681130140120193902

111

3624191914117113111112

19410391535766582689127117142203342196003542667149184359596825020443071403236618612943667330639817465527069912381237021020387211243346334348401319881604486
000000000000000000000000000000000000000000000000000000000000

19410391535766582689127117142203342196003542667149184359596825020443071403236618612943667330639817465527069912381237021020387211243346334348401319881604486

239119917427193564125215954021200384723855964818926259529204515612927911261104958488175384116139159546932318392452748681803349951114026141951489793757286563189538741835195117172179487
00000000000000000000000000000431782389380012211712622387116124684919208271062337459277562431

18121171241113313141146

79455400228347412434111101336052015153447188425672875451561292355067434262761021325886321081065362214233309772561287100224017104507707212062471281592882563455432523

11461923315832501773844291149102391712212113114619233724158325017738144298123110491023917122212113

14969811504583530368331041169320437399318242845335712612931956123137407321339413817151428231052311423205144141014337212241239356323962014

00000000000000000000000000848963766820247193018999628663571797287115981917021629075131131541117124136589230
1461361113143546415711062421020106290807217984601945141939441376412978246221138208543117111785879332823945613512913755476913063771517105728727840818814916245148111351592156341

3505106479748302353130313539497919412117128353211413525212121415

146136763925400157110545136118039376769166346019451419394413764129782462211382085433013991321113119134291675369874688735323611260117930183813539722766106

000000000000515100313110021013203010010630400
1831861143610819413084145132871233827279112229151793311717231399861943757921122814

149194130841451328712323229191545289411585554143332121462611
00000000000000000000000000000000000000

149194130841451328712323229191545289411585554143332121462611

34186114361081026206211077123521672242413347223
0000000000000000000000000000000000

34186114361081026206211077123521672242413347223

31526272100451089145901035425982118889149641401940505950974376828244269382167426814323524664329293101451438116105256910763239
000000000000000005234110394162272510300001112599221113

42598211888914969714710653113753281783095136443182918442137813141631021
0000001171065218342771572674585882922557237117711841021021

97315412121121261

42541110618149631343820142725

499221132

1

571583710514221268211

000000000000000
122363131252715

122363131252715

111111
000000

111111

3152627210045108914590103541304940505950955275417161532210151238181020821431282244936752165
000000000000432330211266038183213311301121961341

51121

1

312062721004510899889405059509302349910311110411551037103

10541247915125131133121

32145904741199111314821134161111112541

0016354400000000002000000330402
422647474511812123271934112416511012

1111221112111
0100000000000

112211211

111

0023200000000000000
42262726111127163411414

1

42264611127163411414

31111931
00000000

31111931

213199209115314318123201485581363514965876342514
0013000000000010000000000000000000

00232100370112441021141121500310103
213199945131431111318146556136354965766232510

11

21316110511111131

111

3

111

1

2

1121111

1

1

1

22

9941314113491355412254

11131211111

1113

1026471212111114
1026470211111114

11

294862451202293429713311265153983242093498713131173283314303854442335226319973226572149518625
0000000000000000000000000000000000000000000000000

00000000000000000011000023024650471841404211003100
294862451202293429713311265153983242093498713131173283314303854442335226319973226572149518625

294862451202293429711265153983242093498710811616211322334633731134114711123467148115524

133241229161261624112261411211

353378264716768279572182067974181664010576121181256116842248182053171334261154613447112944981897831779651103125
000000000000000000000000000000000000000000000000000

0000000000000000000106620019612154253191750327627381725171436384264675
353378264716768279572182067974181664010576121181256116842248182053171334261154613447112944981897831779651103125

11214253341124

3533782647167682795721820679741816640105761211811454841142651112814961125317717746917163911392255350

932621691751031152855016592294373575394083603848110890227327968718322272317832421314148531515866782927913330135129223333630
0000000000000000000000000000000000000000000000000000000

0000000000000000000000000000000000000000000000000000000
932621691751031152855016592294373575394083603848110890227327968718322272317832421314148531515866782927913330135129223333630

932621691751031152855016592294373575394083603848110890227327968718322272317832421314148531515866782927913330135129223333630

4572822359232724541217121
00000000000000000000000

4572822359232724541217121

2927621512291243219532219102751132
000000000000000000000000000000

2927621512291243219532219102751132

412442858304818131437109981571324041400349610341249936399866551616941396683618961941071233998472932272014310167135203556421338225294191355176398323488615385286233768137162598329255315644292
000000000000000000000000085416112926651301568230144101122481074162518766230679583421081474206586

143244214241111528221113
001000000000000000000000

142143242411112

31

1111

111821112

000000000000000000000000002320013632441214821021232220997613285921036762240113180168301613591394
412442858304818131437109981571324041400349610341249936399866551616941396683618961941071233998471210545161181001173162162321939971225118276271425813414609202660231112107167645713463661

22521935811111142422113310226340517105750911582327591295516111911888836150319410712162900974214370566015915814455613697831371602889734351512563201939129522422939602234

18722350019376711015986505487206488324395259173647375752078393177838541112121213361067138171210615403541155884241126432733

513151211116191084419145161613216012541211142
513151211116191094419145161613216112541211142

11

00000
21131

1

1

11121

000000000000000000000
312432113213318127322

312432113213318127322

0000000000000000234181144756190724423466012020481
10913313912636718115919844655599201954621446074482132515716867334452512710124116152127930174152116

1331391261811591984465559920195462184354393286142502232621311939115413114766
00000000000000000000000000000000000000000

1331391261811591984465559920195462184354393286142502232621311939115413114766

0000000000
1121151411

11

11251411

0212300000000000001
10926810352211121721313

1

109243411

443221121721312

113

1111
0110

1

1

0144447120732422220123203104
367666553112317937224412835415338

111

11

6811116512451

1

1

11

14

1

1

1

1

1

1

1

1

299111024212313

2

1

3

1

2

000000000000000000000000000000000000000000000000000000000
3401942101307872236212818655921503661296116219671442162515622319318310813431411182761511711006458842086105469547113939344116210051613111210223920148167691719577115996307906

00000000000000000000000000124310030121112364133400031140502246
3401942101307872236212818655921503661296116219671442162515622319318310813431411182761511711006458842086105469547113939344116210051613111210223920148167691719577115996307906

34019421013078722145750672538150366113110801953141215051562231931831081343141118274149166956148831890678570439132253361025940158910909523920044158651518275105993283899
0000000000000000000000000152114126633730721475586478371114152418748361275940803201574112347141526256442204750

347456522381592156169512283111113201015171112136721

8374213262853463316045503972751221117421171631721195035296152332624

411322211211

27382924729353293462531503664382938274815162233143311412106111672906968491438511369238103104236346315146599

15241832252231110111111219412041711

31315618113054358861349619252112123311161322221734414

000000000000000000000000000000000000
217531193541658214301202131116637512331625131611119661453192511

217531193541658214301202131116637512331625131611119661453192511

1
0

1

000000000
111415212

111415212

97023637044128221194307635001583121524591549926637468243103111265734283431209135834436768214216066313170438359741517990
00000000000000000000000000000000000000000000000000000

97023637044128221194307635001583121524591549926637468243103111265734283431209135834436768214216066313170438359741517990
0000000000000000000001402000121832121563210000411651110013

11112463161
00000000000

11112463161

0000000000000000000000000000000000000000000000
1650441919919824316183812231553755103137362916226135146313144764641482141332013621979

1650441919919824316183812231553755103137362916226135146313144764641482141332013621979

95423632010922133976340213415416283427611141324397142031212235222817173231570114328271217226383888
000000000000000031010101000110421000274563041023

9362363201092213397634021341541628222761114132298112182101941528161218135911431153218623654

18121413122317231108491102211

221

87114112267121153138108
00000000000000000000

87114112267121153138108
00000000000000000000

87114112267121153138108

1193448550865871118553533611864471462125714172631811672110311499431480446425632272244671534393564922631141490310562098112991198390739673839811101202114330223012957841037613861183350115331070103128816246164231405805342591
0000000000000000000000000000000168365499485882129287161537635531904973243237025531688203364336142565689182109522722211342

00000000000000000000000008318018919752498238169133302512920714224317935210141117143150605543381322011461136
437339398882918150368584726382295315495580515466327288340112642631825812593004153191271539156973043014451651043022224272484123521821132122517465640425457150186229

308
318

1

0
1

1

1478321112681027981422177181151682391942018341
000001147815981018143151151052281321305190

14783111133322511671315

1

11121121

1

2111112

0000000000027351518621631064423235439115728284852411134
49181369422238515466209288465815947170933676324658474029311813612013165344923416151321364265

421

32210

1

214411

111

1

241312114111413

1

2

1

11

1165311

211212

1

22111121

1

1

683111244111

1

1531

111

1

1813694222385011384736335143216102411

11

2

11

2111

1

2

1

1

11

1

1

22315112111211

1121121111

5154661092385819311112136315033101132657

49221842532161362141232101

11

1111111

1

1

1

0000
1121

1121

00000000000000000000000000000
36232238819424826426346121919123482121513033242223

3623223881942482642634612191912248212152921212223

111123

0000000
1322112

1322112

11
00

11

992026115414336111734210259323
0182611530133610062011119203

9913142

1

2

114

3

11

1

211

1

1

1

00000000133913107131400201100360100210
26171016174141911332616189521325393264212109226125159111457

11

221114532

11

1

11

414427119161

1

1511

2227111121

261710161719112617108351617151192872511138945

11

2

00000000000000000
885351122441221193142

885351122441221193142

000000001000013000100000110000
698227631549514392131114352102211254310321531541

11

6982276315495143921211322210221025431032431541

31511111
00000000

3151111

1

1441514115914
1441515115915

11

0000012000128501936122534307110131
11824648437222116131481711126915526864111421132

1

121

21

4371111112

1

1

1

118246482229401112131211

111

1

11

111211

11

00000000000000000
13111374511112719

13111374511112719

291921112114113313522614372076203119111415
002100001101002212381211612141611106

12121

11221434914

11113242213819

291912112212315

121

52111
00000

52111

000000000000000040373022765119104203202106154710446695551278553489820017911318115660756
56294266523995862102582153345153724863529630825246564633311816165133249247147277017278190938950120965316229723128162816751141010

3323712123311111
00370100000111

311

332111192

1

1

000
821

821

437824311257121022279321318711222322
000201000110000101000000000000

2167411111

1

437822125242351211361021321

122

1

2131

12

11

1

1111

1

11
00

1

1

000330001201000000000000030
80551210876211148538131128741141171

8055121021121101271271046411211

335283114241214

00000000100000000000000000000000
562635562220112734111657178451215913366444364

56263556222011152311165715442611913256443363

11

11

1

112426411

21426649799861933325153720563521830825241182514134220111133137338292338021411239245
000000000000000000000000000001010000100

1

21426649799861933325153720563521830825241825141342209113127338290337911411139245

1

121111

22311295112165126111352
00000000001020000000000

14112

11

13

123112814123152

1111

12

1

00000
11111

11111

92131000000000010
92143131641443311

1213343431

12111

2

1

832213124111
0000000000

1

83221312411

3411
0000

0000
3411

3411

000000000000000000000000002110101010100010000004101000000
7564090468858711097535307892436572570791578396363126560456016971523375313235622811411282523223611642912130720192020789524717441041262268426147143114831310

111
000

111

000000000000000000000000000000000000000000001000000000000
756409046885871109753530789243657257079157839636312656045601697152337531323562281141115245317360159281212771916199786504517438412622282251451327107888

11

75640904688587110975353078924365725707915783963631265604560169715233753132356228114111424531435915828121276171217678148431542737252198225142132697266

1211242352221413311622

12545231131212111134452
11545231131212111134452

1

000000000000000000000000004021710101201026034331723129102010220
2065242541441322494971438899336349419426316782923273444163125547019352714527191713381231984311601208913334581942523964272407755344394011508121

5513171312838110431317161566193271672625271473443711
00000000000000000000000000000000000

5513171312838110431317161566193271672625271473443711
00000000000000000000000000000000000

111317131249101113171373212174521149271132510

44833232383473112674151222231121

00000000000000000000000000000000000000000
94281043220110362946362942551111766141434191058756181519532121224

00000000000000000000000000000000000000000
94281043220110362946362942551111766141434191058756181519532121224

94281043220110362946362942551111766141434191058756181519532121224

5739251328132249497143889613433011942631642105734441631251831683232171111436711819442315110986831329518023826439121674833633348204617
00000000000000000000000004000011100014104142400001000042200

000000000000000000000000000000000000000000000000000
33201322364971438893329923217726316427573283773125183217853121141824201378584027022616023216385736327301272451778

33201322364971438893329923217726316427573283773125183217853121141824201378584027022616023216385736327301272451778

13163910102115174113162112163543102
0000201101011000000164102

131639105257310161621528110

3211511

411151121212
000000000000

411151121212

0000000000000000
2113713226217317

2113713226217317

24192513282844691730211815201063241259212658106553271221207

000000000000000000000000000000393138483553882532133552564725321628612525912653714215823810933726441826262209411178169650
23446571247018904435674423439194924048643013219137114031000157611844822914221328782354513221017166379461422301469251313979473161580680216104889010786392602223103912187056981923852121266711363151752468514321405481186

2011201992918171615154474636293110482442368321669105282430
000000000000000000000000000000

2011201992918171615154474636293110482442368321669105282430
2010171892918171615154474536293110482442367921669105282430

13113

1

677667281308150519184415267994235187141432961143121487
00000000000000000000000000000010000000

000000000000000000000000000000000
677667283650216164322276121210414116411171335

677667283650216164322276121210414116411171335

0000000000000000000000000000
130453321132452332383133132325152

130453321132452332383133132325152

0
1

0
1

1

39907722845514577406874623312360288788223430471377282195743322232169639167442317342943
000000000000000000000000000000000000000000000000

39907722845514577406874623312360288788223430471377282195743322232169639167442317342943
000000000000000000000000000000000000000000000000

39907722845514577406874623312360288788223430471377282195743322232169639167442317342943

363961715536202674362578161452103758182112111445815314341151238147153270294
000000000000000000000000000000000000000000000

363961715536202674362578161452103758182112111445815314341151238147153270294
000000000000000000000000000000000000000000000

363961715536202674362578161452103758182112111445815314341151238147153270294

0000000000000000000000031491600155403122050541497454118651152104221
641701511143372796647128415718373926315520965394078239510676217941857166635317111029258913227281742311351394345111210365273268983542101183739

7915111458217912201311662571552123163295913454
000000000000000000000000000000000

7915111458217912201311662571552123163295913454

332726176221050773236552823019335751113912913744
26252517422850763236452813019322741113711713732

11

1

11111

1

51

11

1

11112

1211

1

1
0

1

64913372796647128415718373926315520959594078239510676941851511563482576721854147109223031641136192252451319852629
0000000000000000000000000000000000000000000000000

64913372796647128415718373926315520959594078239510676941851511563482576721854147109223031641136192252451319852629

10231715122632211112022341
8111413122532210111922330

211321111

1

1

110257958182631213942619348503319111936194311321871056333041241536248910
0000000000000000000000000000000000000000000

110257958182631213942617112392311118211214132525222821972923673
0000000000000000000000000000000000000000

110257958182631213942617112392311118211214132525222821972923673

22361110811341831098621031112215871227
1425876112317363862931102214741126

4622111121

61

112111

341141

17617818028033483382219137343229325312410215793566826112356914789887445445012422132852401615946352212951511311052810325474530
0000000000000000000000000000000000000000000000000000000000

0000000000000000000000000000000000000000000000000000000000
17617818028033483382219137343229325312410215793566826112356914789887445445012422132852401615946352212951511311052810325474530

17617818028033483382219137343229325312410215793566826112356914789887445445012422132852401615946352212951511311052810325474530

00000000000000000000000000
1510313111342352216129247511

00000000000000000000000000
1510313111342352216129247511

1510313111342352216129247511

31211114422
00000000000

00000000000
31211114422

31211114422

0000000000000000000000000000000000000000000000000
75492152100233206681192523272653187092523555117763259192617796529122592131912964621282217101287127316

0000000000000000000000000000000000000000000000000
75492152100233206681192523272653187092523555117763259192617796529122592131912964621282217101287127316

75492152100233206681192523272653187092523555117763259192617796529122592131912964621282217101287127316

00000000000000000000000000001087740111571899107659704171139311010303
5665853149112837924572561931809289541804713459830113622517326532927731069323883026148180616277219716430574229411212395273831151508910025292512171203321281031752580324

000000000000000000000
3838614415121111913133153

3838614415121111913133153

00000000000000000000000
734572121261304342115131611

734531112512743421151121

4111132141

0000000000
1434211121

1434211121

0000000000000000000000000000000000000000000000000000000000
541583214781283791947256193180926954177669233681211362251731583292759106932388300513217931177501721471740147793926113354946367847221845626306118751249532307

541583214781283791947256193180926954177669233681211362251731583292759106932388300513217931177501721471740147793926113354946367847221845626306118751249532307

1000000
1211111

211111

2521131320282150182414211613201310955123279174191348134651246110561151310
000000000000000000000000000000000000000000000

2521131320282150182414211613201310955123279174191348134651246110561151310

257934410178253441489944611507154443564521229332987137236155241740185170341153217520656431
0000000000030332026900218171720267341633118991661813278424317

9982431362432255
01000000000000

9972421362432255

1

445694122412
0000000000

445694122412

11001000
21112111

1

11

1111

00
11

11

00
11

11

1111
0000

1111

1211113
0000000

1211113

13

0
1

1

0000000
1111421

1111421

124111
000000

124111

000000000
121141921

121141921

15311621321713135213
03310621110313121013

111132

1211121

1

0
1

1

0
1

1

0000000000000000000
25441021117220511114318

25441021117220511114318

000000000000000
121111132521211

121111132521211

114462113252145111423151
0000000000000000000

114462113252145111423151

00000
3213111

3213111

97122121521111212561
00000000000000000000

97122121521111212561

18193441017810761521116814115168324158294612117525513312139
0000000213601101100112020002100010100

12113313314211322

1211111131

121

211

18193441017810761495121119552241242444192244419137

61621452
00000000

61621452

000000000000000000
74123111231752211241

74123111231752211241

1932281123511161192721532114
0000000000000000000000

1932281123511161192721532114

000000000000000000000000000000000000
52836166141533935372372193673039522514123713641431412872

52836166141533935372372193673039522514123713641431412872
0000000231200500314112131966013502121913041

843942111124331114612862
00000000000000000000

843942111124331114612862

3214371142112213211021773111
0000000000000000000000

3214371142112213211021773111

00000000000000000000000
5283296682121211221131771115235

5283296682121211221131771115235

321111241812211
0000000000000

321111241812211

141454616836392120714157654681034331015621232475
0000102130013000002001000000000000

0000000000000000000000000000000
14143341662636177143763468934321015521232424

011302542630052141355426412242651922341
14143341662636177143763468934321015521232424

2112

14132112613512512716283

125113217111

1

00000000000
21192121151

21192121151

000000000000000003084460151111614132242341813914036530923222013817722118710180
137027568662638601188254822459741740454838761421198174263840946567112023659147117854116625292145111166913251610494951910238481787

2874038538185837125525651452530316515462910925635221293869222
0008121635115223823325626012001951116013131182

20001010000300120
32221111212311141

1

12211212111

2

1

0
1

1

0202109120
2312119121

111

1

11

00206100662311185132100300669325200
7838304112222192312181444310259315914115323323

2

3

21

1111

21

11

4612216246222222

1

321

2641

32119

11

18311121185211

3891102

6

1

611

1112

209401017841811131271811524131
008100110000000014201121

81

209402783111312716713221

1134111311431122231543161441

202848211722122452062516154122333214615152
00060000300000105002027000100010

20281316213116514715141325136
0000000000000000000000000

20281316213116514715141325136

00000000000
11122151111

11122151111

4822161122141171211172
00000000000000000000

4822161122141171211172

0000000000004677585512552146917251429635873868829453112322541305115340
1339568663860118825482245977945381022145925662649301592551577071014212518287611160201484354332292219961

000000000000000000000000000000000
3586167118816218338274721111131054142136146233227514211

3586167118816218338274721111131054142136146233227514211

0000000000000000000000
26377315143161313121311352

26377315143161313121311352

825620351794519218451057463263102231447331421
00000000000000000000000000000000

825620351794519218451057463263102231447331421

11572108202123335132521233389433815215
000000000000000000000000

11572108202123335132521233389433815215

0000000346463314461149101023737775270427351835169816172341
1107477178921955185197469565419481265142633047310869283951215646911110172451

11

192227307486701161182120555962122

1

51

111

46820523664042337412111819822624162

4474728534982966897675121721449531881193102115

2

11

5915391911

1

221

000000000000000000000000
275138662135112136462842511231

275138662135112136462842511231
000000000000000000000000

275138662135112136462842511231

2818541359628621777191796975348873654654927542221202218911311898858140354549841511114410767606132721027286153171244710326681634
000000000000000000000000000000000000000000000000000000

2818541359628621777191796975348873654654927542221202218911311898858140354549841511114410767606132721027286153171244710326681634
000000000000000000000000000000000000000000000000000000

000000000000000000000000000000000000000000000000000000
2818541359628621777191796975348873654654927542221202218911311898858140354549841511114410767606132721027286153171244710326681634

2818541359628621777191796975348873654654927542221202218911311898858140354549841511114410767606132721027286153171244710326681634

000000000000000000000000000000000000000000000000000000000
315181841698212339197937233406858210453796514520301268601766113196170581720197331009548633514621252901856814211588334775269730407674101657070125802933616414731088251775495811683510383584292

315181841698212339197937233406858210453796514520301268601766113196170581720197331009548633514621252901856814211588334775269730407674101657070125802933616414731088251775495811683510383584292
000000000000000000000000000000000000000000000000000000000

315181841698212339197937233406858210453796514520301268601766113196170581720197331009548633514621252901856814211588334775269730407674101657070125802933616414731088251775495811683510383584292
000000000000000000000000000000000000000000000000000000000

00000000000000000000000000111211217211334767883211637142117164311556538737167179736319005913292338935993117239248164
315181841698212339197937233406858210453796514520301268601766113196170581720197331009548633514621252901856814211588334775269730407674101657070125802933616414731088251775495811683510383584292

31518184164220793776676576310976568936699812811242054671521954950294966251613310077086268235109643363068811813222171199297139042778471990319824854156179713518960202235136100

56441431171472979261517967170959213932451770114185953618467128981241750595912539269641969423355188813457253315499228

000000000000000000000000000
611235216322132211712552231253

611235216322132211712552231253
000000000000000000000000000

611235216322132211712552231253
000000000000000000000000000

411035013102132010711541131253
611235216322132211712552231253

2

1121

22211

21

11

1951102308971596431041085217947550401775120151122411800901948551161014847362334248093217245277211174119188666157682379847200618358390170425449832910055197487201314272059801734482368178214612284610859451172263723492832487391469902169949111072969813227943016515700014667018379013523516044612186833539816937725013813454435830160053330914557517918122730136756195434
0000000000000000000000000000000034227702966059733168108951216094971304143396053438531303907793808288905396838968639604327070749950130780743508995063286120979865218354192385410670433885076400

00000000000000000000000000000000000000
25162120175315474220962172101161776515523142610255352923

00000000000000000000000000000000000000
25162120175315474220962172101161776515523142610255352923

25162120175315474220962172101161776515523142610255352923
00000000000000000000000000000000000000

25162120175315474220962172101161776515523142610255352923

55597799968643928222013212064284464812418010657545301317271455946218118633145385957924126229089681276108281726926022760255511743215031559858123618473341594015718876167334228
00000000000000000000000000202519118161766239281011134233624256753156120427542732104

392071311271074138124122411311639318215753522
00000000000000000000000000000

392071311271074138124122411311639318215753522
00000000000121392821005752501

41112123111

39207131127107281241119317164911

111

5979951326028222013212048569033678838143130181145433323441246197248231155435612510736437334917631769927644830795451131619301122318
000000000000000005479356625232798111038274929315190413314130821

00000000000000002318822341330621411788725348368220227222136271540252437166713676109476119
597995132162822201321204856903367882551301811453828425381653901982071283348114963213412901412786462674482128241961416301022116

1

1114

1

1

597124282220132120485330891901301457891327213536202088100181646391211442013

921112131354212

2

1

1

1

1

181411231

112

1112

1

1411

411121721

11

221

3

122611

122

9911217

41

53111132

36059825514113562491162161153121

1

3

2475331831

1

212

1

51311111104434413113111

11

111

1161923

44126431321251115510682541013121
000000000000000000000000

97132151162139

442943201554661151013121

00000000020000110302000000002010000
1733019910364912323643171813384546762240826493735727645274934164811918938

1
0

1

0725391206622151311333742691038025473695717521254731124210818736
17310354912321643171813373945732239126483735725531274832164711918938

1

11611

6

1

212111161211

2

1

1211121

1

1

1

1

11

3

1

1

1

18

21

11

2112

1

111332111

1731144111132

13

2

1

0000000000
3019915115121131

3019915115121131

000000000000000000768311101320212061063010616203901311184
554063157154282124180706234035362284513835413345212312318862538253126777026549456513111838196308515568224321

40211121321181
000000000000

40211121321181

216185911113132
216085911113132

1

13821
00000

13821

0000
1114

1114

0
1

1

000000000000001000300010101060000100
13015418212418070623403430228451383533114334445110630354612693221551439715412

12

131

130154182124180253962033281584513835332444511053034167745164381439712410

4522720010270129484851172

5517551441561111184329
00000111020111071001

55175513154113327

2

11

12111
00000

12111

0000000000101000001040110
6345681312201338421541732465131

1

3136

1

4568111328321141316211

631191472142

11

000
311

311

113583218424375223
013373214424350103

14

212512

27411115522911683122623314303418315213
0091231031123321300012241381913

1111

132

274123211345101323162926110

0000000000000000000000000000
55383411922163273134316826421273

55383411922163273134316826421273

411412642192611
0000000000000

200410310152400
411412642192611

211232

2

1

32111

121313571491457872999896717137452931505292722934055925257574751266144
301656146144780199889671613337262543478262253395562455535460164141

11231261117357411212222141123
0112001000402120000012101900

31

1211

1

111

121

13

111

1111123312111

1211111

01
11

1

84116531121421
84106531100110

1

1

111

1

12

1169427716437211711315122213558794220941
000101000006110000411001111210

116942711168111318145478315611
00000000000000000000000

116942711168111318145478315611

314320121025121301
514334151129141412

1211

1

1

24122

1

1

0000000000000000
326211614285141012591

020000305510522391
326211614285141012591

12216

326116113432

1156516078960291412774433289706459531449219028504616121535445435515653103303489435731921018104345709555513301025938534642206487161846163963134471646497810884983473288407743028134583910769787254016568227973484111818777618361344
00000000000000000000000000000243114102614010706314816610755201337563844691187510017146791912111870156

00000000000000000000000000042045004220321511160002526121151520323
109521555896028435184325775953144921902850461480673403129364000103303489213831928621043456045225782984714334642160656939530219612385225538718219199883571954753397874927811011135329061349414154226

31331
00000

31331

51114161111131
00003111111110

51112

1

5

1

00000000
1521113212

1521113212

0000000000000000000000000000000000000000000000000000000000
109521555896028435184325775953144921902850461465660403129364000103303489200431926881043456045225782984714334642159056039029819013312055337092171757535419045633576524165892431726958846410148218

109521555896028435184325775953144921902850461465660403129364000103303489200431926881043456045225782984714334642159056039029819013312055337092171757535419045633576524165892431726958846410148218

4611112111121
0000000000000

4611112111121

000000000000
134174111754817141

1341747548173

1111

11

13181412129221211
000000000000000

1314121292111

1

8211

1182285101133
1062265101133

11

21

61352070693143328944871328624236151653221915610533054841224202151881219840361132426117629220417293220350504249092271510609637083663358994741153141172449811062
00000000000000000002162794313222591826126214132117231521235910466295135096934921424260263326711428165277743

0000010110000040200155518754021
19145189432189111145344647082412612256

14511521

1

111211

11111414

1

111

211111222

11111123

19157321511127141173

1

11

1

1051419922111134311258365
00000010033000100

105141992111311257365

21

14111121
00000000

14111121

01101000100120113200100
3111711134221149115211113

1

1

1

11

3115821

11

1249213

1

1

00000000100000
31111112213112

111

3111111211211

24272620311812612312317456854342914102108127227619254226720617516917861582650297295
0211731161251231228643685434281498108126727519254126720617496816831579650296295

1

2427124111

11111121

11

51211

21

1

1111

21

1111

1

1

00000000000000000034240040100413208020003031016316140120
59420970693143314420601328624236151653221915617744941224201331953214226209326911261862726153106133397122633103643203989

1

11

1

331

151

1

13

1

1

1

5942097069314331442060132862423615165322191561774494122420129113281412613935842518225261457413389110242243183969

000000000000000000000000
12621423629312215314511115

12621423629312215314511115

731311231114212
220100010001000

11112112

1

41111

1

111111

00000000000000000000000000
4219726817212205321223232238254242

4219726817212205321223232238254242

0000000000000000000000000000000037921331240862736271952671448216113491245193429238333411888268924133170685040279448527235935112346
11547953316587281987530451670956641728758930921176424966070320303371616064576670123559444012568667651179431139416087664158145602034872713451446851910699156478101441115246414919933245721613394601792244815474989553929311012401480102355017535139541339298340266777414313051005562

11541953316587281987530451670956641476698930921176424966034319743371616064576670123511444012568667615179431139414642247130145601896092713450030851910699156225101445872762261062084017779216932425916329840745267973619716537576189176241521748517139491334819192
00000000000000000000000000000000879548295511125226618675606720198229144534210125766049975994155234029380

000
312

21

111

000000000000000000000000000000010000011100000000000000000000000
5339750252424921846211690281022849805838376563878251875629741376337150229193430142455213285179439511198093107247057210326306833950401050991235288301885470317181618735796932111512101282987681108332722710029228971322121420042

5339750252424921846211690281022849805838376563878251875629741376337150229193430142455213285179439511198093107247057210326306833950401050991235288301875470317181518334796932110512101272977680108322422610029228971322121320038

111

311111213

22211111112211

00
11

11

2873244438581291452677803285259371938363176111626931387113413471929968281255189245585915643621633126320627242412159369236221221382416551510211465018
00000000000000000000000020010002000200101100005101010100

91113

11111112

4

9631031311115171251332

1

2261111111132146561115

1910244438581291452396803285242941938363176111626931387113413471929968289331892229859135325311281441557563331951307325181101054213795372016

211

112144

22

281164332237731125414531532621594711255

1

2112112111227

1
0

1

0000000000000000000100
222699230212510030214725531191173843121627

22269923021251003021472553191163833121525

1112

0
1

1

1111
0000

1111

1131

2

11

100000001
115411111

1541

11

1

0000000000000
1223111121171

1

122311121171

001001101001
111311211151

11311115

00
11

11

25359412524112154493111136655162106
014100013111424220401040000001

1

11

253412211016161215115192

111121

1111111411

1

1

1211311

1

2
0

2

13
00

13

0000000000000
238423331121621

238423331121621

0
1

1

2
0

2

7539539123092757670927784017713571925373739758222740061116215107179121752152149702258324604341121635455714418627181161772146391085925696106976555581272928931151422233
0000000000000000000000005640011012000013200102182002122404

3

21111

1112

1

428405341110618223111626

32

44413841971111213

1925428422414101151211141

709512309232967092778401773309314681621110341497618728117619702244484604341121635358134151710466345152414462857064771329153241161716

1313112

5391357407606400612813117912619345345511712667043246281123484381111420

12421
00000

12421

21111
00000

21111

00000
7611111

7611111

111
000

111

5153915552837444953255845188653902482441392231042588850235802217834068710128451181711511107287249675821622528199
000000000000000000000000511012002000002000000

11

1

1

515391555283744495325584518865390248244139223104258885023580221783406871012835108121041197087229575821422528198

213

1

7181691221251537668261313123272611493421417124
000000000000000000000000000

7181691221251537668261313123272611493421417124

00
13

13

1143

00
22

22

0000000
1111122

1111122

0040100
3141121

1

3121

000000000
112112111

112112111

1

1

0
1

1

11

000
211

1

21

00000000
11113131

11113131

111
001

11

231232
000001

132

1

121

1

11

1981121221132217147
01000101102111034

1

1

11

196112115

1

1111

1

111

1132411

001
231

23

129803814345170281038714039466478222046256147884017151142811324716265431832369921266
000000033370194103803219590170483881533612131415

11212211121
10000000000

12

1

2221111

12609381318217028103871403112217693126173121513426417128254
000000102100221010000100100000

311

12

12609131821702810387136017483512625216114142

1111

111

3843911047112114111

1

2121112111611
0000000000100

21131

11

1112

11111

211112331427312
000000000000000

11212

1111

11

2214622

1

1

00000000000000000000000000000
302116383116122211633591194188911732

302116383116122211633591194188911732

00002101000000000000000400110100
699496482416314611112344821163351012171

699465241310324311132348233511101

1111

1

2131512281213141147

0000000000000000000000000000
14111814171933141319199198232021523132

14111814171933141319199198232021523132
0000000000000000000000000000

14181417132171771481216212

331

11411013221513223132

21111

251220223349115921221621435554655155319524652391527225
000001000000000210200210000001000000

0
2

2

0000000000100000000440110000110
25273491159171212129313252312515321237143145

6

11

11

226

3491159311192222131341312102

1

125

25271311218111227291112231

1
0

1

1217523314211121123221221211232
000000010001100040000100

121

1111111111

1

1

12175214102111521121

1

1

2310

000000000
312121111

312121111

0
6

6

23241311314
0000011

2324131133
000000

2324131133

10153825258154715118156791524031774
0001000000010000000000000

0300031154001000001
851725231141211112162

11

61112213415

11211

12111

1112

131

1

3

1

21
20

1

1
0

1

211123812359106146671323821154
00101381234910613666132372744

212

1

1111121

12115
00000

1215
0000

115

11

1
0

1

21133634134513325721155295710189611847117208358814721458167
000000000001001000000001000001110000000

00000
21211

2111

2

11
00

1

1

22131863128112316625951233212811263
00002000010001000000000000101

1

14

221318112211151985123115241151

11

51315142111

1

212

1

22

2113361227131512231105715531102151201242114562
0000000000000000000000000000000000

2113361227131512231105715531102151201242114562

1
0

1

1
0

1

1114734104121115191341512211132
0000002000000000000000000000

511711351126111

1

1

1

2121

11

1

131131113

1111

41

3232331

11

1141

293649613009557521729879918610071626117301187151286578123227221605757930526732103831912976757937418961006072616897155245357478182421335392575617721199815773215679652158711168312477231728151915757475846266125621644118974643638
000000000000000000000000000000000000000000000000000000000000

000000000000000000000000000015158333833273633475880456494246511291085223474239170151461011415426164216826272
293649613009557521729879918610071626117301187151286578123227221605757930526732103831912976757937418961006072616897155245357478182421335392575617721199815773215679652158711168312477231728151915757475846266125621644118974643638

22264961300955752172987991007162611730118715128657812295322160575793052673210249182596585793001896100605371689710522915401730201119499173510702111176926671067482204469983289772431471133813636955706248622517138617193893352
000000000000000000000000000392066480863816194386121410675104071694448165031820069792141624011371146106253205281708170822568813151673

2511511121

2111

11314

1

1

12

12421361171111

1123311

1

1

211

1

3962774477726912124572196166147361331351382325102

6

311

211222

11411

7421251211492415221226

111231112

31006344539432093195111231651541722161115421462215

154153123343319112111241211761

21964942173055946371121704155266268542650389800932901492011337132027128881144137741675739311028126

1

1

1921226168049810150123244283215341472222344161213324127161

1

39787244088458541327042711997198612326171166823751243874312534

4463322

12

1013251312

1122

522111513121212351

1112117211113211

61

2112

31

4323423112121

1111

3088287412044312304292111

1515

11

121111413

1

1

11

2111111

1

122221

131

11

11

2713911112111

1

12211121

1

1

12651024231311112311304481112332

11

11211218221

310125101399504632721122882562789223172

521241

13

121112112111

212

11

1

1

611

1216121

1711131121

11

1

3773110

131

68339670911103298494100710325359835698916381188365657172102039461732929957930013969939269168971315537930910593716362281455600971984481329146125922128704355989797361644

1

121111

20111211

711862741341097418930212482449412791581612893614954671121529994813
00000002102102001120342310000161051221220

11

1371341893432252211228127541433121212

111

1

2

33203431121122101522416310114172112371

16912491321116

21

21

3829711094312621213456345181233191111114244

1

000000000000000000
87521511211421431441

87521511211421431441

101
111

1

0
4

4

11111
00001

1

111

000
211

211

37
00

37

00000000000000000000000
403576591616272125514912543635112

00000000000000000000000
403576591616272125514912543635112

403576591616272125514912543635112

00000000000
111715101512111

111715101512111

304828680141415094621892513417401682344512152381009119114107112221171
000000000000000000000000000000000000

48680141415094621899272867101441042371009018414577121161
00000010000300000200012000000000

2040011140031
2341115141231

31412

1

3768014141509409189231411512361009011913141
000000000000000000000

3768014141509409189231411512361009011913141

00000000000000000000000
115342110549143411381645115

115342110549143411381645115

3028254210121113312111175410111
00010090010000000000000

00000000000
12111111122

12111111122

000000000000000000
302825210311311116528111

302825210311311116528111

1

317684746438420102495627671345089127605251145371733017033139911063633643683436552591613267106811867958131219463153152354055267260482671524187435844620636109818239872562046801163113280124581251676412194416258480241431045786461549542361063035352117046673812
00000000000000000000000000000000011000000000001140003221034730100

3618212222207531
00000000000000

3618212222207531
3618212221207531

1

00000000000000000000000000000032463702415770344152267278592255150515746186216791683133734689971233325166061222047209832753800465536
317684746438420102495627671345089127605251145371733017033139911063633643683436552591613267106811867958131219463153152354055267260482671524187435744220636109218239872562046791163113272124561251476382190416258478240891041686461545442291062235352116646663812

300534570237579944130559648981436316328166431328410286316736834365556543921337756834077103686156294245236488521544223441964033851911028943173790106921074210486100705959168423812627033461696194028892191803431091626639652999
00000000000000000000000000768684899508129166817641401505298256755384427629989135072121142470061414799912846930710912481384

1644022752321936613274596412412309153251547410066864928583212164933640230630535274280481014464154864315352375756875867214611122411711924660347554742439024886951643411212395861198130982540531574480722381164

1361322950538628283177420541003116932181637309471271622014161532440912732267169213938921136262836980146063313726361110731236014044737624203768341012810137511356054350479451

131011213216222
0000000000000

131011213216222

1318212311311411
0000000000000000

1318212311311411

0000000000000000000000000000000000000000000000000000000000
14444423830236397134479748127962779318663136197214137283150623119360814239100196110932111253022929963722044710046718175911450155661114145731311135

14444423830236397134479748127962779318663136197214137283150623119360814239100196110932111253022929963722044710046718175911450155661114145731311135

0000000000000000000000000000000
212325370921661141811115411311515241343134

212325370921661141811115411311515241343134

000000000001020000110201607301321001010200
7057140332633554334157451469121132616898102013299143333522161203971

33571403326334334157219247121516497813121831951413109342

55121111432142244

2111

3724323411516311

00000000000100010000009531118006153010111
103284515201071485234596813197313252101713157356192471991723142485

76523427523111123384113471412311012

10328451520107723263101663214101412663511431141731362

000000000000000000000000000000000000000
3935291073728273526913116513510281375138853441132511181291

3935291073728273526913116513510281375138853441132511181291

00000000024145673367111534501076196178449164932514815128226124251704354411
67992017140418091268437545102879611312511193550110619818854916593251481513122913046231192594812

2211

6799201714041809126843754267242298058212211133621611522341

000000000000000000000113130382156107513591218197107302894112836568542848383935693893356874
68533132910216102127148178504214197269209702111360138011481166781551434991361363723393165117548211989105431157443913531699769735905951611331051116491150119

13

41643716211816671033

50481640284210267232124843267111442336198311911128111234165263611458327272418910

61244918207659915354581227311331641231221320125131776312725

494560100431211115941335212

107146605238275594660191114828191413715212512

2

6651327721352641218110601117742402009117345306

927120306157292200165511414121

635176183421021279679413131199633733210184377761165619512043965651197551463417022551415316202222170151043722

1

11416711111061

121

11

696441291787768195211521261143265206124121757
00000000000000000000000000000

696441291787768195211521261143265206124121757
00000000000000000000000000000

00000000000000000000000000000
696441291787768195211521261143265206124121757

696441291787768195211521261143265206124121757

00000000000000000000000
452511915226245511959249111311

452511915226245511959249111311
00000000000010110001000

00000000000300
452342262258142611

11

181

452332252255413

321

0000000000000
25961115118565121

25961115118565121

0
1

1

146784997406262819087192071785376248633731618869440117428719691283154717301698273206278944599184961419710251238119397395997054240313972507961020957197688871252613225539922183023295749168971934728109717529599122055030941239341768512352181473412281320425919764948525082
000000000000000000000000000000001131518191118474454301007182616616151518311732410525504112993201

301811271234139732212113231112287178158511312
00000000000000000000000000000

00000000000000000000000000000
301811271234139732212113231112287178158511312

301811271234139732212113231112287178158511312

121642701110072184114151312105289174497462420742572931104316114441542375870100001138640690882321105442019
00000000000000000000000000000000000000

00000000000000000000000000
1216111810331111622511165101810562

1216111810331111622511165101810562

00269111381050591574955623199405424207281110444134198584599351130639488875314105042018
42701007231111213111052791734973624201405524209371111444154237586099991130639690877315105242019

88415471819111215819211

1413711

3561513711

41211113121

33868525111110218

77413161

145

00000000000000202579544688934151428826216151251435342256434143676372342565571214165
69518716108813261428734204841242449920338877613267524014215811881585438821304372716828656474648265718021648109147334207385973430320

0024251311172400001
2449924251312172511292351

24499111129235

00000000000073971428082275110117574101713103313252511952181252757321011131316158819151
695187161088132614287342048412420338877611241614598902951170295901040161140142930320742391364880361562151329761226155

821220338872112561810111196

7613814

14273311132511212313

1

1

83144011131102251115122726322

6951871345882048804111154261252944911111

343992221971192814161

1

1001326412614111

1711141

111112121

0000000000000000000000000000000010144999911330814081732887211822353309231311332972250461301051295113725
134899972192598190871920717692762398337316056094401174285395312831072659169827286627893828114127412578111211191947220954240312581699174878217758747119711214463486116740314164824812171964999480930188820465302781777896852133526846134224681594894224530

0000000000000000000000000000000000000000000
3540564734625115945057173126984963145164036133192011102981311491703237232011

3540564734625115945057173126984963145164036133192011102981311491703237232011

1
0

1

0000000000000000000000017556962913598023112171067053319815888300654551607129337182542466594135522071313962619514761392285716244553823796
7762316190871913117631162022335876030692391057266557410217071347233615950336759120333790248535023101422658064315308692119231207863459016646312884723705154338484047569657161892420015409762616761682396121244834324405

7761594387367032172180069775222622466594151063444663375017634751505715051067722972124613186190163110355890214510216215102213433

11251342323207362136972931771516166

62331205872504835223754817110414185622291396

964437865190417061914263382109988211391092241242118131051086834156111412

722888572109740644016238123804467734631155574102170756145771313413867393212061290313441574407527127741250671102415667147131044313506228249429278843178112

000000000000000000000000000000011023013030082123281014510471210102217
13489221131247365603161442541541021543172112062092943908328615221630335384100311912272209669160262053201001324465159771177981118151113113106112804153135577492264195490238642580

16391431132122511434822122

1348922113120536531316144254154102154317211167209294469309313442189347100311912272209669160241952188322343715972117787211113810969931101552745192291964145473218242169

11

1310237101237519211

423439297712099431461191116011258925212911

1111111

11112

81121024333169182502992784873224140172171987
81121024333169182502992784872222140172171987

2

1

0000000000000000000000
152127118292232218265676543617111

152127118292232218265676543617111

000000000000100000000000
601522432121231107104129110111881

1111

6015224312123110784119110111781

1

00000020302116421310572124231000000
881012816474344221431612624125178432671

2

35112422131423722371

2

88777152142131164213

69311578753841482333542631892103712454645347522265252011369978118113118763604935498354624353405221971016620708189259795380115743042844874442782421331447281951157978138850184591240
000000000000000000000000000045318129141113223866292211514850533371331946257939687252139

130454232134135821225913724616123931252251155
0000000000000000000000000000000

00111012303182020538717278362162100143
130454232134135821225913724616123931252251155

2111

1

1

1

1

1

11

1112221

111

13045233231221671

112351

11

11121336

1111113

121

356360875384423413226311486263422718928478842858859926518186839491723141822026418242715637134399171637293312215011917824319336475617910840476351232413437149
000000000000000000000000000040317731100914621033953332858636919131915796832648118

000000000000000000000000000
215861853256513632882241465371351

215861853256513632882241465371351

000000000000000
8578111132131213322

8578111132131213322

0000000000000000000000000000000000000000000000000000000000
25331382738442341322631099476342271891912154859971112756494348182201621253231102512226495508234197105801150153182847428322202341714109

25331382738442341322631099476342271891912154859971112756494348182201621253231102512226495508234197105801150153182847428322202341714109

00000000000000000000000000000000
36150111530210867916210233761212273111216511732181062

36150111530210867916210233761212273111216511732181062

674748286913716455698350104432121138483132561076190946201348381144342659919
000000000000325521000014643953646131116561512342729

691371646932501042212311183629317254121152864

67474828555138171563383132414241141258117142416

3377971061772227444119031926415513762914351320828311300235691771131241111476014628644783524929809016811731110464632738251433113910184832
00000000000000000000001300001186885100361502083493113590125

00000000000000010100000000002030010000
3021303518422937763113371311575417172323115410397439491112

30211327138511221122

303518422937761417171321112923713615112

1226293

262334334111

3912034105137496175964921918109391219365378131110169123141144
0000033665954821616983781043322592412113110142

521111

923487375321148222111423622

3

299121

20105501211311855713521

111

0000000
1212231

1212231

0000000000000000000000000
12255140143310347119551442752942112

12255140143310347119551442752942112

0000000000000000000000000000
602338622254536633111241116752331771

602338622254536633111241116752331771

33740686752017444119028922955133444752311300235551779823572229207573026635231878211255318876151613
0000000000000000000000000000000000000000000000

33740686752017444119028922955133444752311300235551779823572229207573026635231878211255318876151613

00
33

33

000542044117386697803025
11454204411861274981013135

114341211

1441

22366268662092174865911141213838425321814117531512604338648015
00000000000100000008314610001168010831232

626817911411101223375241
00000000000000000

626817911411101223375241

1430211895314103719410211
0000000000000000000

1430211895314103719410211

22365274865811421231111311112522310145612
0000020000000102100040233100

91212

223652748656114122111111151645412

341

489972113165344663283121122111311
00000000000000000000000000000

00000000000000000000000000000
489972113165344663283121122111311

489972113165344663283121122111311
00000000000000000000000000000

489972113165344663283121122111311

14014598674555478830154997941937079362166449547809818314474247983024573728577757837438738190872752679895944789108171823445753568265917831330247208113293771543240267926115886953887698025145818004694517074043223162685669373484676594836191154383589879103837141854415251370433212031149443030561359
00000000000000000000000000000000990307444373210546768784573606846310120766416577511692126183552238850433400075584326056133057483056270431055505212372288022621077120661978749164

430172525301987336224103278404113612593239332814712104670308909299502081722427167306831823211485071431025235932154816544525510109302174799248354276898694944116736308476223852014301060124373882893272842117225310813431183677788526447415484133373476

971287342153560546813089466353296575010712561452841119342817768213364377836958559472200812255452056504523047982941230230271914220712321220945033424044964154015614016143616412108516589667853371487290132251155255677377592414101757414903425027151994560585800203771818719

14684124483034976321194568812135238171039713276

0000000000000000000000000000000000000
516395115501653818414186410132510451752913198618137018252955

0000000000000000000000000000000000000
516395115501653818414186410132510451752913198618137018252955

0000001412020101912241013720443416122811181233
516395115501653818414186410132510451752913198618137018252955

51631155016524162272111329324954511142771721

951161111111

1

000000000000000000000000000000000000000
53402205358262674818157216165018215439211315654725526144181941163226324862

53402205358262674818157216165018215439211315654725526144181941163226324862
000000020224303210003010541161155519971572913

0
1

1

2202671312611713177144441235121297218414339
000000014020000062007170110410

17112

8512312421

220267475115494113112813628236

1

1

12

1411452117

0
1

1

1111181110146112
000000100106110

211

11572

111

11113

251541415112
251541415102

1

6

00000000
11211311

11211311

000000010000000000000001
5340267137221622111621211911281

5373175112218162

2

2

4

111

402617211221362118

00000010000001
13411293715471

1

1314

2

4118747

1

11

000000000000000010
311311153283112113

32

1

1131115226311213

5358241341
000000

5358241341

12223114312112444
00000000000000000

12223114312112444

00000
23121

23121

000000
212241

212241

000000000000000000000000000000000000000000000000000000000000
10713451023853302732472161155354717095360571502710966284153106822187497414421941339172005917126022538172143774794112667321378725523524091785321194442728517191

10713451023853302732472161155354717095360571502710966284153106822187497414421941339172005917126022538172143774794112667321378725523524091785321194442728517191

000000000000000000000000000
242512351112223211145315241141251181

024010020000101030115021000
242512351112223211145315241141251181

0000000000000000000
241941416221242471441

241941416221242471441

432132514216121474
43213227106111344

3711113

11122111
00000000

11122111

11221111
00000000

11221111

000000000000000000000000000000018950161626431228698571953703181698432557048154919818931878169220306
7399233415402961016288887794681656935619945024738962169095528823805831136197922511289744204910097262932632381317647962224548117829230312624393334172735793278221962239034841526980180874194817511620523356992307235611715333911764

00000000
21811211

00000000
21811211

21811211

14726471822883334197584386151021161124139514871914181167246243313531022267940152013
0000000000000000100000003000100000506144437100

000000000000000000000000
443319128281114211335141143190125

443319128281114211335141143190125

000000000000000000000000000000000
221822152323524351835481211617510213331077796

221822152323524351835481211617510213331077796

14726254446354386159592711201545816230327111142637112
0000000000000000000000000000000000

14726254446354386159592711201545816230327111142637112

000000000000000000000000000221183363939263824235223225754814872701622262167134780846611771336124993141797
6931619048188727615699024339186278532286524594222357526353397883994951114237185493204677800676334742820376103148100166027820114914478146143416422481110765711256667417735521481193283103287761374320511334

1211424111
0001411000

212

11111

1

3131
0000

3131

00000000000000000000000002301832113152315528671713531401094911475473965633722190874692428
1162518188727613239788355095085865245154585341431123423025711525707181962214106776476334341112151216910010775277331697591767808418145394121921287221297777733140327019509

1111118341123

11617081887276132397883550950858652451545853414311234230257115257071819622141067764763341129448384602242510258814136683089627861642881164203854482931622297081

1151615

81312111

000000
113122

113122

111111111331
000000000000

111111111331

0000000000000018911131222119843103307102583091121952152817354217364904734194117
6924671126303576914314724319285711311975713010115342831201431044828546793761432154484220761614437446334534618

255143165911313

69246711263035769147243158757113119711151311821384125772331323903937254166

143341316153515111441551

2981711512125243811111
00000000000000

2981711512125243811111

00000000000000
11111244151151

11111244151151

000000000
211112231

211112231

97211225128111631
021111010001100

111

28

9715621

00000000011010170958815541431225402321218211201000144
2001019791366386937907654583521812371997825541441745111166235095853229533111325

20010197913663869379072245818221391142145904975672858321171

4335711161111

12

000000000000050010201201000002512013676291000000
6341920652524268791209288542241991101222363526213451215205184825675262967667505952416441

1111

634252426879120928854215499812211126084581164320217693743252169251

31

21

192065872951411122101228221191153428519

1

11292813212111

11

00000000000000000000
16641829241378756248411111

00000000000000000000
16641829241378756248411111

16641829241378756248411111

4381322125513453323973217274513611757661519197691011106991836
000201022031134201182194735150387543466522922

000000000000000000000
438132212211137712716293331221

438132212211137712716293331221

133133104399223152261512991233237913
0330139439722311226028388232033810

11214211241

213263

2

211612285
010100000

2151285

2

1749133413121138141104122836832921164721
00000000000000000000000000000

000020000100000011536011719114620
1749133413121138141104122836832921164721

112

5

17491334101211371411041113817511

00000000000000000000000000000
81222181135561140361539101449210324

11011061013412321413048441315023
81222181135561140361539101449210324

1157591

12113431411

322115111

421131

1111

1112111

183216369359745517677997431052213459449161712113711413761121243183
000000000000000000000000000000000000000

183216369359745517677997431052213459449161712113711413761121243183
000000000000000000000000000000000000000

183216369359745517677997431052213459449161712113711413761121243183

488923341540238537172236165010846736404106944478576182613402155682489126121646110145384184203661281047158181349029317856925120941215721526215311247426279255651864152108197
000000000000000000000000000312872662124652421168413222455355791543070966123215142329

00007102101003260711110
13411753767671642791231421312011611

13411753767606407811311711351

26543525519542937615642432143689381457211231226994110234613715165365571213220
00000000000000000000000000000000000000

26543525519542937615642432143689381457211231226994110234613715165365571213220

00000000000000000000000000000
57404131325324261131111122211311611201172

57404131325324261131111122211311611201172

752666170100101000102442830
8428108202412212791134482862

6231211222

1111

31112121115912221

2141581313272542333

00000000000
11113231121

312

11112311

000000000000000000000000
314419421142356101212111121273

314419421142356101212111121273

2979233415401211721961650819207385215265409605383606148159261216461101621101143638193112878431757154315673229941032229990225824210333613971834042
0000000000000000000000983393591424726637160185165012178832093611106312295204437161237

12

621

2

2

2

1

1

111

11

16

1

1

16

1211212

1

11

1

111131

12

1

14221121111

11

111123

1

3431113321122111112

1

11221212211522

297923341540121172196165081920735121526529149033860614815926121646110597310272544854244853613522801214314316546195137360163262

1

311113

134

1

1

118115453111444435111

211000141102112
211011341132112

1

123

112
000

112

2361204229866421
00000000000000

00000000000000
2361204229866421

2361204229866421

000000000000000
38114132641123515413

38114132641123515413

3111103101
3111126111

231

0000000000000
291611111315152

291111113112

3314

212

200122927592884373958114023921667819382042694444317893714268125912110917176442026612713623614824461985394369385282011087528575167180211515923422552878877148925347358282203243
000000000000000000000000300000019001030310320002353912140201

118109627592884335958114023341606764842103996303485210841261217317115926240262216127108885264967793851108113973291508654314915317192721776987941642712568152
00000000000000000000003493112424639034638222941832318358573239612815147622382771935047045

0
3

3

000000000000000000000069138182143645234215564412197414171659275830491426781159737454846739491063
118109627592884335958114023341606764842103996303485210841261217317112516929241814816985120188557291924882142719354152537721202195953871075129277868107

212111

1751221421373122

5771425428017105210317620741133126536143

33237191171163311

51182211035142131143312

12652921853352811155112541713094432811265420861118247214106335172315801882341231129277815574110

31122330743646312561977824218540417446010213138242101

413173526353111175351

1131

1183216770034718010446728421018662812115110321373117710161446577081939106322385112125

340721211921117931121115142

11

1

184173511171232314973111

2831

1291611123124

111211

3432942118153211151111662311

1

724677687231314476915125087163211

23335225121

211332

31

00000000000000001118911672102520353918105127201523165927100826176483912332511
82133385861553812059448144853428113336602158226101114220211339113275339159140912731302461601468411081513152624901583053115513590

611220718107965374298554310131625361415521459
611220719107965384298564311131625361415521459

111

1

501122207848211184020423897811320390171103983115127720181167121104966
507122207848331274020425197821320391175104983126127720281174121124966

141451

6128911416121

000000000000000039000000000010000000000001030000
8213338586155381205944814485342811333678149513323312111328101393636233444273143942110228446164

11284

2143102113112101

721530381205923986662213744363511310111361021121392017325307312351111

20128581214424832121383948110325577512129101831

61613823612581192218353711183915452163010602117183333

4181142244223312313

11330125425786467814482976213952493657148715824507212245829711885285945382683919200737261171898137692490503555710579143936001065111665583546001445310633098986443431441661157561765772599277919552649159514721126406523729851015225204674578913812973590
0000000000000000000000000000001810371016891366276198190210616712329159739755082263603703772862165224158219204626731292504144711

947489694573386512038572702681151752282132631108121532982147334812616817748272529785736061160129953575977765301871855594325522548283124841244204811304206702722442708138479991065594857687877018434401120250827843246852029
00000000000000000000000000000020303823223416272371510248166155480226317591159141839751864711773817239862198865976613429362361310

562437439344425925177482047188740844122103282736920123117258191111319595770496318743228112461244
0000000000000142000010422500354232227333043503

439344425925177482047188740844126642161841165917684933076429162631

1321111

561251911311334211

531611132211

2437227723162417315531731331

000000004315354141134522110437282209172885444743717341881591938454163
792343109238243106168563432541418120493215264115123731571660124778128210310411891682140057167

572259109165128658937721332113241125082527118524172477

220847311541791861626111111411617122161515123192934

21111251112242322421
11110250110000111421

12

11242211

7436215217317811678461322843791287053435437199610361718117215491041101446637192442351127882140333622741827
0000000000000000000052171293025006370337011101201251631132

7436215217317811678461322843769287053435437199557194911421287103571376634162372345282037283462430795

1211

22113

12

196323821712148623834621420521062316252861681367461602702121215541128549498842002134476729183138113781313922292123471192822340183986823345331516787181121844263
00000000000000000000000000000000000000000000000000000000000

196323821712148623834621420521062316252861681367461602702121215541128549498842002134476729183138113781313922292123471192822340183986823345331516787181121844263

8652022867412666546829635172871011203135221826112560231622121562572343975991510346328
000000000000000000000000000000000000

8652022867412666546829635172871011203135221826112560231622121562572343975991510346328

2612271643123434141211333
00000000000000000000000

2612271643123434141211333

161378761193925354129981341718652111620935316561203832221122287022873
0000000000000000000000000000000000000000

161378761193925354129981341718652111620935316561203832221122287022873

0000000000000000000000000000000000000000
79122184938611471837264324811832112577419150847911617084414112131825191851024821993495078

79122184938611471837264324811832112577419150847911617084414112131825191851024821993495078

7629859304691642315312143161261074466159081693941441516172115337214961241094113633732253181164621
00000000000000000000000000000000000000000000000

7629859304691642315312143161261074466159081693941441516172115337214961241094113633732253181164621

93718722818314533615626263118192543621348202401136036936416311721277644316550124404392720238728191904734316845133
0000000000000000043061303514013086256293610103323052318014217515632838691728

12431225129431

937187228183145336156262631579254362134820240113170764491404158141294021812942121611143015260815314

32529224955182106927625361172347010523494211056212332684431296415191086356841399563812105511458571715112615301616676115
00000000000000000000000000000000000000000000000000000

32529224955182106927625361172347010523494211056212332684431296415191086356841399563812105511458571715112615301616676115

51617631807181818769572522272414383133865174418632242927662246937473717664217421682481417864581434739416345150220444837731766227948806254501392180480554821512229471
0000000000000000000411166134768569111722021105502289139542056394154276626614248632629187441328452135148428

000000000000000000000000000000
309176325146722282255128143311221141431275243

309176325146722282255128143311221141431275243

0000000000000010010039318484890019413110124262951731100
17861818187667022728977714031863224280249217473748345573501702099593923361556717232873776137410376010

17861818187667022728977713361863167280249217473746344373457325114921311025217184421037655734504

6757123112142

23125216

1113221

11211

21912423870573651115422958824105219731167282613161152064188491368862630
00225685135471053219486241002195301372622516801964184481258822530

219124211128148311112

1111

9253114211364

21111331

00000000010000100
31212111117211112

212111

11211116212

0000000000000000000000
21491068111113110442241192431

21491068111113110442241192431

0003110146000000200110345231200
1802871612781584761244016741214417282912

18028716124757161244016541147251712

000000000001232465118545152721465107108756240210001880
27423831338819212524141243024651190481533436683512322483128119821912018911

2742383133881921252410122534142121592619956131121611

41223228323422325812

000000000000000
321316111211812

321316111211812

000000000000000000000000000000000000000000000000000000000
44342845922881842337319535261533236720291249023751200374936520823892614578815201984281811301646215814238761207331183336

44342845922881842337319535261533236720291249023751200374936520823892614578815201984281811301646215814238761207331183336
000000000000000000000000000000000000000000000000000000000

44342845922881842337319535261533236720291249023751200374936520823892614578815201984281811301646215814238761207331183336

000000000000000000000000000000000000000000000000000
1505302852252043223350452162291531823729191118329471091508854133414241719691620148251528104722311239802113721

000000000000000000000010000020010220100000001240001
1505302852252043223350452162291531823729191118329471091508854133414241719691620148251528104722311239802113721

1505302852252025223350452122512681031458853122110131517386105341143312737110062

186229153182372699167614764111349222188942114141016923993039113118

5424172611247812328630119727024105210747527581091172358422217910089703362392668149118619046333711371536775119181581507320629520576593371590731521981543599478189020444448408141292
0000000000000000000000000000081213002215412171781704288158111171362452412997574261431960

3072101181247897211229551882428053224717193472648212322726055642478751445878771128825159181683019138385337523212135918991284438
00000000000000000000000000000000000000000000000000000000

3072101181247897211229551882428053224717193472648212322726055642478751445878771128825159181683019138385337523212135918991284438

1726757259185858654095278997413447371461143114167366358339911843342162616171342413473042133714216918285107367411171820156186
00000000000000000000000000000000000000000000000000000

1726757259185858654095278997413447371461143114167366358339911843342162616171342413473042133714216918285107367411171820156186

00000000000000000000000000000000000000000000000000000
2182071438364187156119160121172358348742224798173715527411329152711714531935510276332956322353304330550321335301122036228

2182071438364187156119160121172358348742224798173715527411329152711714531935510276332956322353304330550321335301122036228

00000000000000000000000000000000000000000000000000000
1622359118649321751981257125275162117435180122223285931772022063231015151187282322344572384923161251910911963198230108

1622359118649321751981257125275162117435180122223285931772022063231015151187282322344572384923161251910911963198230108
00000000000000000000000000000000000000000000100001000

6444971142634510721502412

16223591186493217519812571252759873435180122126285931772022063231015151872822183445517504131542318589562198128108

00000000000000000000
4821111173151121117112

4821111173151121117112
00000000000000000000

4821111173151121117112

432001175017302917559756773655004617812211638432816665719223130216532343100632416153227111352362644934430566756429828224404745913510951284622
000000000000000000000000000011120001111033640141312222196030320700

0000000000000000000000000000000000000000000000000000
431727650291515595235429453406299280152356916158181403589372051251399334307452430426225574481213134899502106358

431727650291515595235429453406299280152356916158181403589372051251399334307452430426225574481213134899502106358

2841173024384419364755493036384326643651506272351323710144112263121519152526304016182761920321468133573151114
00000000000000000000000000000000000000000000000000000000

2841173024384419364755493036384326643651506272351323710144112263121519152526304016182761920321468133573151114

00000000000000000000000
722077353614333183813171112461

722077353614333183813171112461
00482726001320351106000011

72111124
00000000

72111124

0000000
2122161

2122161

000000000000000
7220205812118221144

7220205812118221144

1424271257206072629178017146142408105188212966944424161027611882414065471925441221133635328241756972104019111933113451993635847440626456821247329017131901062694990152932619647726282589277593325
00000000000000000000000000000000000000000000000000000000000

000000000000000000000000000752183988321141223612190140474638622570556275064624434573661156812819521683
1424271257206072629178017146142408105188212966944424161027611882414065471925441221133635328241756972104019111933113451993635847440626456821247329017131901062694990152932619647726282589277593325

000000000000000000000014853034131529160541709126114245914814713272235422234058739365116193715586
142427125720304167517801276220878374253444473372661188228445977749758282415391867994114404726129725022218171549118317317111337852651508150696289493350210156

11113

1

1

11

115

1

304117537116187332761219261243

6811111124

2

11

7

1

121

31

2419140108034113144562275331522511125

11

167512767676185344447268823875977749222045917332495504241814131111943117393277201522616

1

1

11115

1441314921111121223

1

7221

1211

1

1

1

111111312

11431

86910

1121

165148112212114

231111

11

22

2

17

2111

114

121

11

14241970772046574273361124577586041286211451963314915192314272378832044153

111

1

1111111111

23112

2211

14111

111111351

221311211121311

1

1

2

2175132

111

31

1711

11411115

1

1

1

24111424

413010011

11214

11211114

180118861
000000

180118861

0000000000000009115312919978445246312002954351511104
70639820026812913830152641119254447257835321714665921922969723287188102126263512034272037317171723

1

21

1

26

182112111

7063982002681291383015264111925444725783532174456115455215231126610231234852820173113614718

31

21

61

1

11311

000000000174537146536303657679113213323088922131691424523423719116420614751
30324840614140135154559015920524617718154373272552518431757379652417172162856910243292164211415063

1

1

31

1

303441311122

1

1

3195311423112102113225

54021117

40614140135805901591320184721112222111141555551

1

3111213

248606142515441525211113163

211

12

2

1694651132234

0020110000
682251416421

682511642

21

0000000000000000
11141111711314119361

11141111711314119361
0000000000000000

0000000000000000
11141111711314119361

11141111711314119361

6212422911369352514004618916482119302024427719646332678031455158810903981562963063088672117599697166942479232371917524712911549110058693812620711329121194303862024417138639693020481038192127
000000000000000000000000000000000000000000000000000000000000

000000000000000000000000000000000000000000
33245733161571923673120241058421221911834874510519425637952116111

33245733161571923673120241058421221911834874510519425637952116111
000000000000000000000000000000000000000000

33245733161571923673120241058421221911834874510519425637952116111

58822911369346814004618916449119302024427719646332676421398156910903958562963063088672505996661669422768223211171226127115471081585937126199110253414920336012401693813332211546922191116
00000000000000000000000000020604225224718394410012439141827210121316

4117
0000

4117

000000
171611

171611

55322911369346814004618916449119302024427719646332676421398156910903958562963063088672599666166942273820207164221124110131033567898117183102243312515295892231473292415181339616189109
0000000000000000000000000027112159292680367173326392418552514112139486953230003736

111111271111

1

2142111

1

1

11711111121111

21

4111111211222321

577371223

515183659328198119403874101989554272041482355196614238945756511671024457005151603213379324527123374130128652249024

5532291854163280717991450980561004618165374292676421133156910901603366348831617672599666109435762991737460492629149234250437947112248112013543659011487295146048

1

21112121111

02011030101042111148331011
5081641311014521112495141121

506143141112111

111

01321
11321

1

00000000000000000
3519111175111221221

111

351911751221221

1

2911926125019236134211016381150243373375533070608226398594042213338122914587571843912252603464329445003316630242841094812948745301718254929716281732176
0000000000000000000000000000000000000000000000000000000000

2911926125019236134211016381150243373375533070608226398594042213338122914587571843912252603464329445003316630242841094812948745301718254929716281732176
0000000000000000000000000000000000000000000000000000000000

2911926125019236134211016381150243373375533070608226398594042213338122914587571843912252603464329445003316630242841094812948745301718254929716281732176
00000000000000000000000000101000327610122021001311643171620210121

14292661671146

102225113351

1

291192612501923612401101638115024337337553307060822639859404221333812292949118139122425934128344883116226212841084512846111316923490911528018129

1211

1211

77722144002171918914044818221818343100312193644012987163366256383573503349020010115551422455321213601110710598181314696722523717101217215
0000000000000000000000000000000000000000000000000000000

77722144002171918914044818221818343100312193644012987163366256383573503349020010115551422455321213601110710598181314696722523717101217215
0000000000000000000000000000000000000000000000000000000

0000000000000000000000000000000000000000000000000000000
77722144002171918914044818221818343100312193644012987163366256383573503349020010115551422455321213601110710598181314696722523717101217215

77722144002171918914044818221818343100312193644012987163366256383573503349020010115551422455321213601110710598181314696722523717101217215

17003552451749543686200227715622044241175269296514112144876708245573885457041624677723550660514655485479189439803000750328988484167955215525707852531265166720907043817361523135958005301492253585571467358422400168092541311901435777387114925466364
00000000000000000000000000000000231236129112790562015712024696220219211925157864131743561305143224

411620231117146251278311642411104142910242416231
0000000000000000000000000000

411620231117146251278311642411104142910242416231
0000000000000000000000000000

411620231117146251278311642411104142910242416231

00000000000000000000000000000000000000000000000000000000000
45312692772651345130358031173807248151615034479568518769022991153861055424065714453821150202159563901777729711014025312211207567127017991242521238134441043414114087523461115515447193625106839728486819284776

00000000000000000000000000033016769993961032079801143547113315751042181007118337102915937760204489139636117057195035426980117803899
45312692772651345130358031173807248151615034479568518769022991153861055424065714453821150202159563901777729711014025312211207567127017991242521238134441043414114087523461115515447193625106839728486819284776

3521141

225

1902657092705804222124810307878941708926902299115384755465758638228810004691538237341984012157812321512423439517810329321722784439523648120832

12

1033590749402112181326111724414224144

1

1

1391079263636307529964861978345495011085986210211265122206167227611911592123728220152111451366141423

31414716800523372915143435661313713112762172232

182121111

5851741121232314

1

2401111127112

21113111

11

1

33221

6141113217

1

1655055245162264368620021297699110817238122707112120061546701121328605127534447812012593670927746663033544300061898772528125335774246166410298535582711523881686979844863881581174542953773333911981074413197478283326961885671359
00000000000000000000000000000009561016153165778415085626913686642722335429323461409410822923072930542117362501991264995163515

0011021001010002101
3112331212111131121116

111

1

11

1

1

2111

311912115

00000000001000010021020087338212000
334621174244111662231518241210191574513110442756243

11114216412641

33462117424411155131118231091114324111714143243

00000000115000
41113231115121

11

4111313121

0000000000000000000000000000000426336391026402172465605221112352305518639222621021141162458363632497347
1655055245162264368620021297699110817233432661112120057603560117821613649452037831500578968622746663033544300061897042248274251118136781682325219610198198964671086844184282148131922522135827089207761803685351815

111141123

1

60915752243109142160231214118343210201601163607291127777245162165949441105639851294864103213096

1

1131

11

2

1

71321141241

1230901336100284117363311

1

2

11

11

1

1

45119

16

1

12111212

11

29119941142221115

1

1

121113

1646955073160464362517725184059311030006084163131436810136225111122151212810641122232

11

12191411

111111

1

5811211101111111111211

1

1

1

22

121

2121

1

112213114331

1

331442111122612112

11921115

1

1

1

1

1

1

3113

3

11

1

7

7121261

2

1

1

2111112211

1

11

1

11

1

1

1

11

11

1

1

2

1

1

1

7661106721352162215418918217344145137542011181416222315151728218275112310

1

2

1112

1

11

1122

2

1

1

22

11

1311

122

61

2

1

1

1

112

2173213122419112151

1

12

1

11311111

1

1

1

1

819618023067268711100400289112863822629937752840235130728412922756230315510534414424142474121713978155141353029183443287519229015379513677199823

72

81110222

1

1

1

11111

1

21221

1

1

14115611

11

4314195300279631115262165141412443164156153642

2

111

71

11

1

11111145212

4114

1

41

12111

1111

1111

11213

1

1

11

11412521211

111421111101113

1

2

1

13

1

1

1162231

211

1

1

1

111

231

1

21

3221

1

1

1

1

1

11211213

22

1

1

11

1113
0000

1113

01014315021110091043512501010100
2920621431915132216891547181371312125

11411111

4241321141576214

2920258112

11

1

0000000000000000000000000
183623505211411361461125132891974

183623505211411361461125132891974

00000000000000000000000000000
4363732216571478824998468142280112111772920831251502915813520

4363732216571478824998468142280112111772920831251502915813520

0000000000000000000000000000000
3038593929375311122443332531746562042571984

3038593929375311122443332531746562042571984
0000000000000000000000000000000

3038593929375311122443332531746562042571984

121
000

21

1

10372907113531209670754361814781481824726269428161776116074386449161202311424769032975302271701218434108432677839618040101848541629148215588757472710286818981258810178550233237412435409923795872471737257473
000000000000000000000000000000000000000000000000000000000000000

10372907113531209670754361814781481824726269428161776116074386449161202311424769032975302271701218434108432677839618040101848541629148215588757472710286818981258810178550233237412435409923795872471737257473
000000000000000000000000000000079253300110636224226217910224622193224921032

00000000
12111421

12111421
00000000

00000000
12111421

12111421

878201671353989670754356212411481824650265627821668885648864491611909106019554728245618516432054189172336933731524010082152657012221445850664264828131787989714164520215223111542636603595092041671239455
000000000000000000000000000000011947132819914129106126306266100815194331381711452922966421362232546474263882682

33517314122411114323452
001000301110002200000

000000000000000
33133111111012141

33133111111012141

11111
00000

11111

000000000
431312221

431312221

839201671353985955542956212411481790650265627821636885648504491611230793195547282420185164320541872715750525512923595313364218261131689565203122561503754509612241069835372053562643591641206199355
00000000000000000000000000000001373661441341275297111210968119240264344127119288224263129251104044122023062

358791576211368155365811926315882518272591993932360137503478591025711930979140554519226632442818765220674367471724131
00000000000000000025944108342778477840792044660438387152521932018534615593643151302421

7316815987239112421572722262052522411471

35879157621136815536581119463429164827236199395515281478221640100265394106546318184387202138109

000000000000000000000000000000000000000000
34241853192445752938119194735118734103812148346624147136782676356

34241853192445752938119194735118734103812148346624147136782676356

26202822767053794127135409785433920344616452218251118175243366328175361661393512814262646
00000000000000000000000000000000000000000000000000

26202822767053794127135409785433920344616452218251118175243366328175361661393512814262646

00000000000000000000000000000000000000000000000000000000000000
143494037362626257951001034540338462934424404142576444661322524763161602825558151814244141592941415227181632485602311996281830317491582035106714138

143494037362626257951001034540338462934424404142576444661322524763161602825558151814244141592941415227181632485602311996281830317491582035106714138

000000000000000000000000000000000000000000000000000000000000000
108734392544533269259399966967576303159316793274202175017264295213202562352277611191381636633253136365242041632405306214432091013116826129514113462619210617891411625866498112

108734392544533269259399966967576303159316793274202175017264295213202562352277611191381636633253136365242041632405306214432091013116826129514113462619210617891411625866498112

000000000100120000000040002400100001
170351713203710542461511734612161531214423331911938397

15235172020873204143214331359111101823235

1813171814111213291323714121783635161

3111311111101541313
311110111141301312

11

215231

3936703637162210533111943151981830321382613111563681111421041413
0000835312551951349132522031421325204

367011111211121136419222
00000000000000000000

367011111211121136419222

001271342076624563614771103370214541321904
39362813167312567321017413191811311571063041083853949

112122

1111121

2111

112111113641341

11121

7122113

61

1111

393610311123133151152113143

1

1

11112

21122122222

11

112311111833271

1111

371001143432576267148272143258670103516328533931102613417440177721141632222
000000011103205714072631362030126744107320000

0000000000000000000000
140903118214141444336324429

140903118214141444336324429

000000010713151263428719111223626129170177194952118321422
3710011434324361771071617232071549241322436292510201191130311231211532222

1

26

19

371001143432436177321114352625210231313249317113218

5215566522373121392255379412646191421739181521311463
09320400121012911123131219912362294921111403

5261362362115776266210414
0002301000000000000000

523122332115761614

2

3111137

132313

11111

1002182356237357514384200
1112192359237357514984212

1123

2

121

1

1

000000000000
121931144112

000000000000
121931144112

121931144112
121730143101

2111

1

0000000000000000000000000000000000000000000000000000
1598940225623776383410827595114822813561574425813161588260182517553224912078363310425985761211370272309156941561315

1598940225623776383410827595114822813561574425813161588260182517553224912078363310425985761211370272309156941561315
000000000000000000000013830000443200100000001006000000

13761225623776383410827595114822813561574425813161365522142217553174581476343310425985761210869272301156841551215
000000000000000000000114451310111112010957619142026102543361719293982323

1

1

32

11111111

137612256237763834108275951148228135615744258131619109410643264656201378249831296851253272115932321012

1

2

0000000000000000000
2228402124301361621412111

2228402124301361621412111

2033845739262447312833283796011387252
000000010000000000000000

314231
000000

314231
000000

314231
000000

000000
314231

314231

00000000000000000000000
203384573926212311283328379571387252

203384573926212311283328379571387252
00000000000000000000000

00000000000000000000000
203384573926212311283328379571387252

203384573926212311283328379571387252
00000000006320101004201

2033845739141921131327978561344131

122234131112

356225152113711375302316211222728129593232519645112
000000000000000000000000000000

356225152113711375302316211222728129593232519645112
000000000000000000000000000000

356225152113711375302316211222728129593232519645112
000000000000000000000000000000

000000000000000000000000000000
356225152113711375302316211222728129593232519645112

000000000000000000000000000000
356225152113711375302316211222728129593232519645112

356225152113711375302316211222728129593232519645112

000000000000000000000000000000000000000
45331091342711458601215710355135131650167109615736212731818621424616687

1291312211122124221
00000000000000000

00000000000000000
1291312211122124221

00
11

11
00

11

00000000000000000
1291311211121124221

1291311211121124221
00000000000000000

1291311211121124221

4533109134259145851111549353115131650167109515736112611617601022616666
000000000000000000000000000000000000000

4533109134259145851111549353115131650167109515736112611617601022616666
000000000000000000000000000000000000000

000000000011001000000002000012000000000
4533109134259145851111549353115131650167109515736112611617601022616666

00000266110040020230000002000110300
33101636143571111841233310791513354414515361

33101636149111441310107915133344144561

4575342235816433824474111317617814436122412134657613605
000005333002864111215106214401185123432641225

412121

1

7913102112

1

45753414458782161151133428811256

14345966221349344322449241571314197833134164352062311633654132455217851911552231417814
000000000000000000000000000000000000000000000

14345966221349344322449241571314197833134164352062311633654132455217851911552231417814
000000000000000000000000000000000000000000000

0000000000000002000061723031109724015234014332501
14345966221349344322449241571314197833134164352062311633654132455217851911552231417814

000000012221510000010533000000
11541566324122871162933173532128231056152

411
300

11

1

00000000000000000000000000
11541566324122719314217352265201056152

11541566324122719314217352265201056152

3
0

3

00000000000000000000
42623323327122916232

42623323327122916232
40613303205010203101

2111231312

11221411

2844221349344322442757141052135272123125362134615225538432812827311
00000000000001010101000932020000101010200

1712112131511
00000000000

1712112131511

4422134934262244571454292621131022418195132245832277414210
00000000000000000000000000000000000000

4422134934262244571454292621131022418195132245832277414210

0000000100010
28275261121127311

282752611212531

2

1
0

1
0

1

000000000
13133321610

000000000
13133321610

13133321610

33131213141111102135
000000000000000000

000000000000000000
33131213141111102135

33131213141111102135

000000000000000000000000000000000000000000
12235319611643154319355925917732118897162115719146919322458151531178142451968142683201512

00000000000225237296040001201065389122241507220523
12235319611643154319355925917732118897162115719146919322458151531178142451968142683201512

19264832846225393152415711033371532817663
0000000000000000000000000000000

19264832846225393152415711033371532817663
0000000000000000000000000000000

0040257460100201201046000132113815511
19264832846225393152415711033371532817663

2

1

1

44

1

11

11

262132

11

19111111034221

1

1

252211121131

1

1

2111

112212

854315432361579144253964415151747461223708101092272672
0000000000000000000000000000000000

854315432361579144253964415151747461223708101092272672
0000001000010000003100001000001000

00
13

13

000000
111181

111181

431543236975225185401513143523121250410992261672
000095100500290652502121148382120211

21

111

431543236211331157910211267223146

1

42711

00000000
22121111

22121111

8547071281311113
012940281311113

8534131

00000000000000000000000000000
1222681961163533239215812960211482311117623322134

1222681961163533239215812960211482311117623322134
00000000000000000000000000000

1222681961163533239215812960211482311117623322134
00000000000000000000000000000

1222681961163533239215812960211482311117623322134
