# Supplementary material for: DIVE: Diversity-Informed Valuation of Ecosystem Functioning
Source: Biology (Basel). 2026 Nov 9;15(21):2047. doi: 10.3390/biology15212047 (PMC13762180; doi:10.3390/biology15212047)
Supplement: Supplementary file 1 [file biology-15-02047-s001.zip › Table S1 Alpha Diversity Metrics.pdf]

Table S1. α-diversity metrics applied to the read-derived abundance distribution of annotated functional transcripts. S_{obs} is the number of observed functional annotations within a sample, S_i refers to the number of functional annotations with abundance i, $p_i = n_i/N$ is the relative abundance of the i-th functional annotation, n_i is the read-derived abundance assigned to the (i)-th functional annotation, N is the total number of annotated non-rRNA reads within a sample, $\bar{n}$ is the mean abundance of all functional annotations within a sample, M is the median abundance of all functional annotations within a sample.

N°	Name	Formulae	Reference
Richness: Richness quantifies the number of distinct functional annotations detected within the annotated functional transcript profile of a sample [1].			
1	Observed Richness	S_{obs}	[2]
2	Hill Index	$H_1 = e^{-\sum_{i=1}^{S_{obs}} p_i \, ln(p_i)}$	[3]
3	Chao bias-corrected	$S_P = S_{obs} + \frac{S_1(S_1-1)}{2(S_2+1)} \cdot \frac{(N-1)}{N}$	[4]
4	ACE	$S_P = S_{abund} + \frac{S_{rare}}{C_{ace}} + \frac{S_1}{C_{ace}}\gamma^2$ $C_{ace} = 1 - \frac{S_1}{N_{rare}}$ $\gamma^2 = max\left(\frac{S_{rare}}{C_{ace}}\left(\sum_{i=1}^{10} i(i-1)\,S_i\right)/N_{rare}(N_{rare}-1) - 1,0\right)$ S_{rare} is the number of rare species, S_{abund} is the number of abundant species with a default threshold of abundance of 10 reads for rare species	[4]
Diversity: diversity metrics jointly characterize the number of detected functional annotations and the distribution of their read-derived abundances within the annotated functional transcript profile [1].			
5	Shannon	$D_{Shannon} = -\sum_{i=1}^{S_{obs}} p_i \, ln(p_i)$	[2]
6	Inverse Simpson	$D_{Simpson} = \frac{1}{\sum_{i=1}^{S_{obs}} p_i^2}$	[2]
7	Gini-Simpson	$D_{Gini-Simpson} = 1 - \sum_{i=1}^{S_{obs}} p_i^2$	[1]
8	Coverage	Number of species needed to cover a given fraction of the ecosystem (50 percent by default)	[1]
9	Fisher(α)	$S_{obs} = \alpha \, log_e\left(1 + \frac{N}{\alpha}\right)$ α is Fisher's index of diversity	[5]
Evenness: evenness quantifies how uniformly read-derived abundance is distributed among functional annotations within a sample [1].			
10	Camargo	$E_{Camargo} = 1 - \left[\sum_{i=1}^{S_{obs}} \sum_{j=i+1}^{S_{obs}} \left(\frac{\lvert p_i - p_j \rvert}{S_{obs}}\right)\right]$	[6]
11	Simpson evenness	$E_{Simpson} = \frac{1}{S_{obs} \cdot \sum_{i=1}^{S_{obs}} p_i^2}$	[2]
12	Shannon evenness	$E_{Shannon} = J' = \frac{-\sum_{i=1}^{S_{obs}} p_i \ln(p_i)}{\ln(S_{obs})}$	[2]
13	Smith Wilson Evenness	$E_{var} = 1 - \frac{2}{\pi}\tan^{-1}\left\{\frac{\sum_{i=1}^{S_{obs}}\left(\ln(n_i) - \sum_{j=1}^{S_{obs}}\frac{\ln(n_j)}{S_{obs}}\right)^2}{S_{obs}}\right\}$	[7]
14	Bulla evenness	$E_{Bulla} = \frac{O - 1/S_{obs}}{1 - 1/S_{obs}}$ $O = \sum_{i=1}^{S_{obs}} \min(p_i, 1/S_{obs})$	[8]

Dominance: dominance quantifies the extent to which the annotated functional transcript profile is concentrated in one or a small number of highly abundant functional annotations [1].			
15	Absolute Dominance	$absolute\ abundance = max(n_i)$	[1]
16	Relative Dominance	$RD = \frac{n_i}{N}$	[1]
17	Simpson's Lambda	$\lambda = \sum_{i=1}^{S_{obs}} p_i^2$	[9]
18	McNaughton's index	$dmn = \frac{n_1 + n_2}{N_{tot}}$	[10]
19	Gini	$G = \frac{\sum_{i=1}^{S_{obs}} \sum_{j=1}^{S_{obs}} \lvert p_i - p_j \rvert}{2 \cdot \sum_{i=1}^{S_{obs}} p_i}$	[11] [12]
20	Core abundance	$core\ abundance = \sum_{i=1}^{S_{obs}} p_{i,core}$ core species defined as those occurring in at least half of the samples	[1]
21	Berger-Parker index	$dbp = \frac{n_{max}}{N}$	[13]
Rarity: rarity characterizes the contribution or occurrence of low-abundance functional annotations within the annotated functional transcript profile [2].			
22	log modulo skewness	$R_{LogSkew} = \left[\sum (\log(n_i) - \mu)^3 / S_{obs}\right] / \left[\sum (\log(n_i) - \mu)^2 / S_{obs}\right]^{3/2} S_{obs}/(S_{obs} - 2) \sqrt{[S_{obs} - 1/S_{obs}]}$ μ is the mean of $\log(n_i)$	[2]

References:

[1] T. E. Borman, F.G.M.; Shetty, S.A.; Lahti, L. "Microbiome Analysis (mia): Estimate Alpha Diversity Indices (addAlpha Function)." https://microbiome.github.io/mia/reference/addAlpha.html (accessed 6 November 2026).

[2] A. E. Magurran and B. J. McGill, *Biological Diversity* Oxford, UK: Oxford University Press, 2011. Available at: https://global.oup.com/academic/product/biological-diversity-9780199580675?cc=nl&lang=en&utm_source=chatgpt.com.

[3] M. O. Hill, "Diversity and Evenness: A Unifying Notation and Its Consequences," *Ecological Society of America,* vol. 54, no. 2, pp. 427-432, 1973. [Online]. Available: https://www.jstor.org/stable/1934352.

[4] B. O'Hara and J. Oksanen. "specpool: Extrapolated Species Richness in a Species Pool." https://rdrr.io/cran/vegan/man/specpool.html (accessed 31-1-2024.

[5] R. A. Fisher, A. Steven Corbet, and C. B. Williams, "The Relation Between the Number of Species and the Number of Individuals in a Random Sample of an Animal Population," *Journal of Animal Ecology,* vol. 12, pp. 42-58, 1943, doi: https://doi.org/10.2307/1411.

[6] J. A. Camargo, "New diversity index for assessing structural alterations in aquatic communities," *Bulletin of Environmental Contamination and Toxicology,* vol. 48, pp. 428-434, 1992.

[7] B. Smith and J. B. Wilson, "A Consumer's Guide to Evenness Indices," *Oikos,* vol. 76, pp. 70-82, 1996.

[8] L. Bulla, "An Index of Evenness and Its Associated Diversity Measure," *Oikos,* vol. 70, no. 1, pp. 167-171, 1994. [Online]. Available: https://www.jstor.org/stable/3545713.

[9] E. H. Simpson, "Measurement of Diversity," *Nature,* vol. 163, no. 688, 1949, doi: https://doi.org/10.1038/163688a0.

[10] S. J. McNaughton and L. L. Wolf, "Dominance and the Niche in Ecological Systems," *Science,* vol. 167, no. 3915, pp. 131-139, 1970, doi: 10.1126/science.167.3915.131.

[11] C. Gini, "Measurement of Inequality of Incomes," *Oxford University Press,* vol. 31, no. 121, pp. 124-126, 1921, doi: https://doi.org/10.2307/2223319.

[12] R. B. Bendel, S. S. Higgins, J. E. Teberg, and D. A. Pyke, "Comparison of skewness coefficient, coefficient of variation, and Gini coefficient as inequality measures within populations," *Oecologia* no. 78, pp. 394-400, 1989. [Online]. Available: https://www.jstor.org/stable/4218880.

[13] W. H. Berger and F. L. Parker, "Diversity of Planktonic Foraminifera in Deep-Sea Sediments," *American Association for the Advancement of Science,* vol. 168, no. 3937, pp. 1345-1347, 1970, doi: DOI: 10.1126/science.168.3937.1345.
